# Myorhythmia: A Quantitative Study of Synchrony and Rhythmicity Between the Head and Upper Limbs

**DOI:** 10.5334/tohm.986

**Published:** 2025-04-01

**Authors:** Mahmoud Elkhooly, Ahmad Elkouzi, Rodger J. Elble

**Affiliations:** 1 Department of Neurology, Southern Illinois University School of Medicine, Springfield, Illinois 62794–9645, US

**Keywords:** myorhythmia, tremor, multiple sclerosis, accelerometry, brain stem, cerebellum

## Abstract

**Background:**

Myorhythmia is a <4 Hz oscillatory movement disorder that has been variably described as synchronous or asynchronous between body parts and as jerky or rhythmic in appearance, but there is no published report of quantitative motion analysis.

**Methods:**

A 51-year-old woman developed disabling myorhythmia in the head and upper limbs (right>left) approximately three months after a relapse of multiple sclerosis in her brainstem and cerebellum. Head and bilateral hand motion was recorded at rest and during posture with triaxial accelerometers and gyroscopic transducers. Recordings were analyzed with spectral power and coherence analyses. Frequency variability was quantified as half-power spectral bandwidth and interquartile range of cycle-to-cycle frequency change. Waveform deviation from sinusoidality was quantified as total harmonic distortion.

**Results:**

The 2.5–3.2 Hz head and hand oscillations exhibited narrow frequency bandwidths (≤0.21 Hz) and interquartile frequency changes (≤0.38 Hz). Amplitude fluctuated greatly, but head and hand oscillations were intermittently synchronous (coherence 0.8–1.0). Waveform was not perfectly sinusoidal and varied with the transducer.

**Conclusions:**

This is the first quantitative demonstration of very high rhythmicity and nearly perfect coherence of myorhythmia between different body parts, consistent with the classification of myorhythmia as a form of tremor. Limitations of the quantitative methods are discussed.

## Introduction

Myorhythmia is a low-frequency (<4 Hz) involuntary oscillatory movement that usually occurs at rest and during voluntary muscle contraction [1]. Nearly any body part controlled by skeletal muscle can be involved, including the limbs, torso, eyes, throat, face, and head [2]. Oculopalatal tremor arguably falls within the definition of myorhythmia, and extremity myorhythmia often coexists with oculopalatal tremor at the same or different frequencies of oscillation [2,3,4,5,6].

Myorhythmia has been described as synchronous and asynchronous among body parts [2], but we could find no published quantitative demonstration of synchrony or asynchrony (PubMed literature search on 10 Feb 2025). Here we use the definition of synchrony employed by physical scientists: two oscillations having the same fundamental frequency and a constant phase relationship, which need not be zero [7].

The classification of myorhythmia as a form of tremor has been questioned because myorhythmia is often perceived as being jerky and arrhythmic [8]. However, the aspects of myorhythmia (and all other tremors) that create the human perceptions of jerky and arrhythmic are unclear. We found no published electrophysiologic studies of myorhythmia in which rhythmicity was assessed quantitatively with motion transducers or electromyography (EMG), but several case reports with EMG recordings revealed rhythmic EMG activity [2,3,5,9,10,11,12], which was confirmed with Fourier spectral analysis in one report [13]. In some cases, the rhythmic bursts of EMG appeared synchronous among affected body parts, but this was not confirmed quantitatively [14,15].

The principal aim of this report is to rigorously demonstrate synchrony of myorhythmia between the head and upper limbs of a patient with multiple sclerosis affecting the brainstem and cerebellum. The rhythmicity of myorhythmia was quantified and compared with reported values in other forms of tremor. The kinematic properties of tremor that may produce the clinical perception of jerkiness are discussed.

## Case description

A 51-year-old woman with a 23-year history of multiple sclerosis presented to our clinic in November 2023 with disabling ataxia and involuntary oscillatory movement of the head and upper limbs (Video). A relapse in multiple sclerosis had occurred in August 2023, causing severe loss of balance and inability to walk. A low-frequency oscillation of the head, upper limbs (R > L), and torso developed approximately three months after the relapse and was reportedly still increasing in severity. The oscillation subsided during drowsiness and sleep, but her husband was unsure if it stopped completely.

**Video segment:** Video of the patient’s myorhythmia while recumbent on an exam table and while seated in a wheelchair.

No oculopalatal tremor was present. The head and extremity oscillations occurred at rest but increased during voluntary postures and movement. The oscillations were continuous in the head and right upper limb, but they occurred intermittently in the left upper limb. There was no benefit from propranolol 120 mg, primidone 50 mg, or topiramate 50 mg. Clonazepam produced extreme drowsiness.

MRI in February 2024 revealed non-enhancing demyelinating lesions in the medulla > pons > midbrain and in the inferior, middle and superior cerebellar peduncles and deep cerebellar white matter bilaterally. Nearly all of this demyelination was not visible in an October 2022 MRI (Supplement Figure 1a and b).

## Methods

Motion of the hands and head was recorded with Movella (Xsens) DOT inertial measurement units that contain a triaxial accelerometer, gyroscopic transducer, and magnetometer with a 60 sample/s recording rate (www.movella.com). The inertial measurement units were taped to the forehead (x axis directed axially toward the vertex, y axis laterally toward the right ear, and z axis perpendicular to the forehead) and to the dorsal right and left metacarpi with x axis directed radially toward the fingers, y axis laterally to the left, and z axis perpendicular to dorsum of the hand (Supplement Figure 2). Recordings of approximately 60 s were performed during two rest conditions: patient lying supine on an exam table while relaxing quietly and while relaxing and counting backwards from 100. A 30-s recording was performed while she extended her upper limbs roughly perpendicular to the exam table. Kinetic tremor was not studied.

The head and hand oscillations were primarily rotational, so the gyroscopic recordings (angular velocity: deg/s) were the primary recordings in this tremor analysis. Gravity-free accelerometric recordings (translational acceleration: m/s^2^) were analyzed with the same methodology, and the results are reported in the online supplement. Gravity-free acceleration was derived from the proprietary fusion algorithm in Xsens DOT (www.movella.com/resources/white-papers/white-papers/dot).

**Figure 1:**
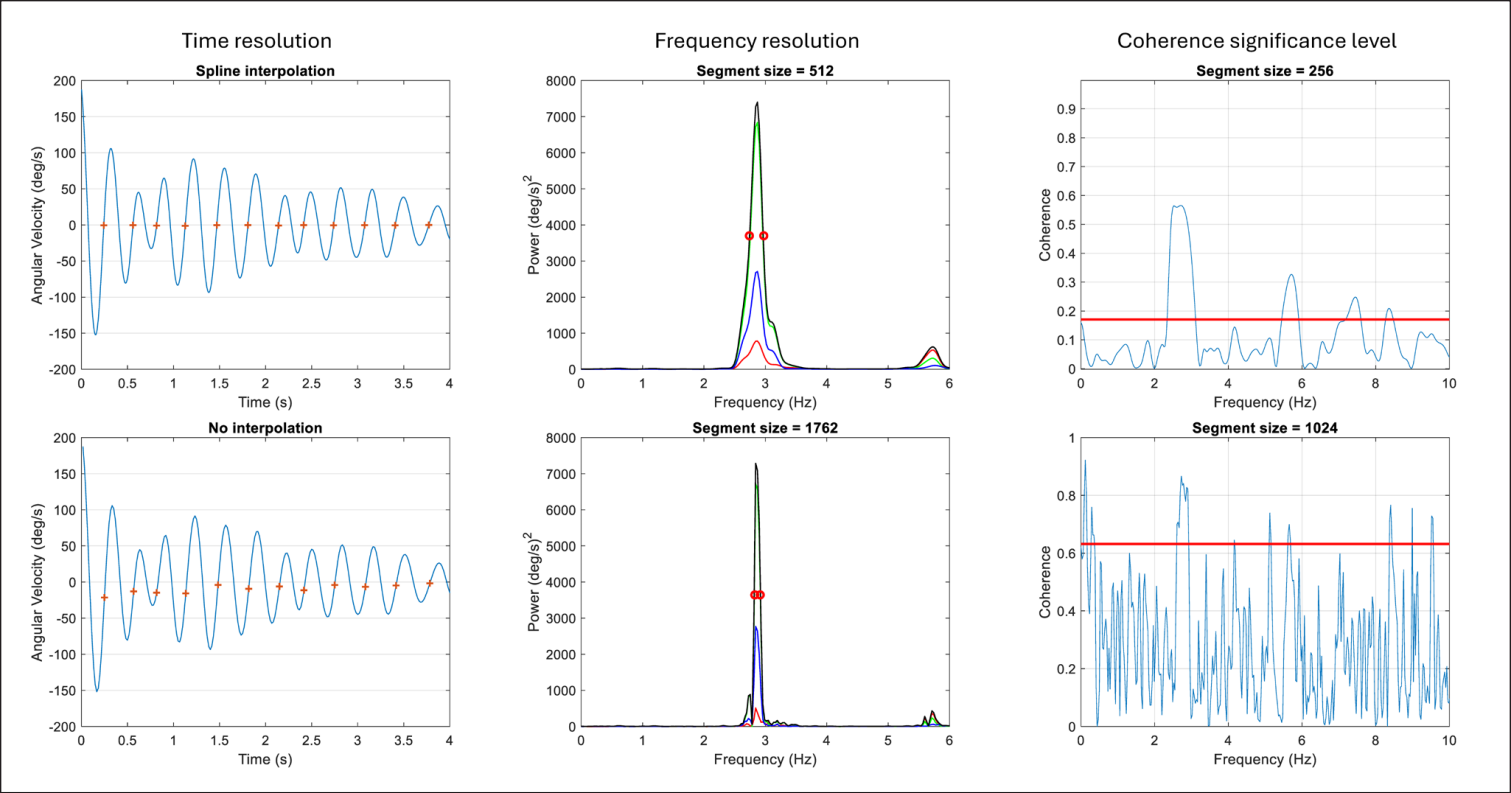
**Effect of analysis parameters on time resolution, frequency resolution, and coherence estimates**. The 60 Hz sampling frequency of our IMUs provided a time resolution of 1/60 = 0.017 s. The first principal component of x-y-z acceleration and angular velocity recordings was interpolated to 1000 points per second using a MATLAB spline function to more accurately locate zero axis crossings (red +) without distorting the waveform (left column). The frequency resolution of a Welch Fourier spectrum is determined by the size of the segments in which the time series is divided. Smaller segments produce smoother (less noisy) spectra but with wider spectral peaks (middle column). Thus, the upper and lower limits of the half-power frequency band (red o) moved closer together when the segment size was increased from 512 to 1762, but the spectra computed with smaller segments were less noisy. Similarly, the statistical significance of coherence estimates (right column) depends strongly on the number of segments (i.e., length of segments). The 95% significance threshold (horizontal red line) moves lower when the time series is divided into a greater number of smaller segments, and the overall values of coherence and number of spurious peaks decrease. Color scheme of the power spectral plots in middle column: x – red, y – green, z – blue, and resultant – black.

Cycle-to-cycle frequency variability was computed using the method of di Biase and coworkers [16]. The first principal component of the three axes of recording was computed with the MATLAB function pca(). The first principal component was bandpass filtered at the peak tremor frequency ±2 Hz to remove extraneous low-frequency activity (e.g., due to wavering posture) and higher tremor harmonics [16]. The filtered first principal component was then interpolated with a 1000-point spline function to increase the accuracy of computing the cycle-to-cycle zero-axis crossings (Figure 1). The interquartile range of cycle-to-cycle frequency change is the Tremor Stability Index of di Biase and coworkers [16]. The coefficient of variation of cycle-to-cycle peak-to-peak amplitudes of the interpolated filtered first principal component was computed as a measure of amplitude variability.

The Fourier power spectra (autospectra) of each x-y-z acceleration and angular velocity recording (time series) was computed with the Welch method, using the MATLAB pwelch() function [17,18]. Each time series of N samples was divided into K segments of length L samples (K = N/L). The Fourier power spectra of the segments were computed and then averaged to produce the Welch spectrum. The spectral frequency resolution is the sampling frequency fs divided by L (e.g., 60/512 = 0.117 Hz). Note that the maximum possible frequency resolution is fs/N, when the time series is analyzed as a single segment. However, tremors fluctuate randomly, and averaging is needed to reduce the variance of the spectral estimates [17,18]. Handling the time series as a single segment precludes any averaging. Thus, there is a tradeoff between frequency resolution and the statistical reliability of detecting signal (e.g., tremor) among extraneous sources of time series fluctuation (i.e., noise) at the same or different frequencies. The spectral power at each frequency is a chi-squared random variable with 2K degrees of freedom. We regarded a spectral peak as statistically significant when it exceeded the mean spectral power over all frequencies plus its upper 95% confidence limit [19]. Our IMU recordings contained virtually no extraneous noise, so the variability in tremor was interpreted as an emergent property of tremorogenesis.

The Welch method includes other steps to enhance signal detection [17,18]. The time series segments are multiplied by a weighting function to reduce artifactual frequencies (leakage) produced by the discontinuities of segmentation. We used the Hann window for this purpose [19]. The segments are also commonly overlapped by a specified fraction of the segment length to increase the spectral smoothing and the statistical degrees of freedom [17,18,19]. The effective number of segments and degrees of freedom are increased by a factor of 1.33 for the 50% overlap used in this study [17]. In addition, segments are often extended with zeros (zero padding) to increase the number of points in the power spectrum. This mathematical trick decreases the frequency intervals at which spectral power is computed but has no effect on the bandwidth of a spectral peak [18]. We routinely zero padded our segments to 2048, resulting in power estimates at 1024 frequency increments between 0 and one-half the sampling frequency = 30 Hz (i.e., 30/1024 = 0.029 Hz).

A power spectrum (autospectrum) provides the distribution of the power (i.e., variance) of a time series over frequency. If the time series is a sine wave with amplitude A, the power spectrum will consist of a spike at the oscillation frequency with no harmonic peaks at integer multiples of this frequency. The amplitude of this spectral peak will be the time series variance = A^2^/2.

Power spectra of non-sinusoidal oscillations, such as a sawtooth wave, square wave and triangular wave, have a primary spectral peak at the fundamental frequency and harmonic peaks at integer multiples of the fundamental frequency. The total harmonic distortion is the square root of the summed power of all higher harmonics divided by the spectral power at the fundamental tremor frequency [20]. Total harmonic distortion is 0 for a perfectly sinusoidal oscillation. We computed harmonic distortion for angular velocity (deg/s), acceleration (m/s^2^), rotation (deg), and displacement (cm). This was accomplished by first computing the power spectra of angular velocity and acceleration and then converting the fundamental and harmonic spectral peaks to rotation and displacement by dividing angular velocity by its frequency (radians/s) and acceleration by its squared frequency [21].

We estimated tremor rhythmicity (i.e., frequency variability) in the frequency domain by computing the half-power spectral bandwidth [22]. The half-power bandwidth index is the half-power bandwidth divided by the peak frequency. The half-power bandwidth is the width of a spectral peak where spectral power is half the peak or maximum value (Figure 1). Frequency variability increases the half-power bandwidth. However, half-power bandwidth is also limited computationally by the frequency resolution of the power spectrum: less frequency resolution results in wider bandwidth estimates. Therefore, we systematically increased the segment size from 256 to the total length of the time series to find the largest segment size (i.e., greatest frequency resolution) that produced a sufficiently smooth spectral peak (Figure 1). We found that a Welch spectrum with segment size L = N/2 and 50% segment overlap maximized frequency resolution while reducing spectral noise to a level consistent with accurate spectral peak identification and half-power bandwidth computation. We found that a segment size of 512 samples and 50% segment overlap was optimal for computing spectral amplitudes and harmonic distortion and for visualizing the variation in tremor amplitude over time using the MATLAB spectrogram() function.

Coherence is the squared linear correlation between two time series at each frequency in the range of 0 to one-half the sampling frequency (i.e., 0–30 Hz) [23,24]. Coherence approaches the maximum value of 1 when two oscillations have a constant phase relationship (i.e., synchrony) and no extraneous noise. Coherence was computed between each x, y and z axis pair of IMUs mounted on the head and two hands. Coherence was also computed between the IMU axes with greatest power (tremor) for comparisons between recording conditions. Average coherence versus frequency was estimated using the MATLAB function mscohere(). However, this measure of coherence assumes statistically stationary time series, and we discovered that our time series were not statistically stationary due to wide variation in tremor amplitude and direction (Supplement Figure 8). Variation in coherence over time and frequency was therefore assessed with Fourier time-frequency coherence spectrograms, using MATLAB function tfcohf() [25,26]. The results with tfcohf() were confirmed with MATLAB wcoherence(), which uses a continuous Morlet wavelet transform instead of the fast Fourier transform (Supplement Figure 4) [23,24,27].

Coherence estimates are statistically unreliable and biased toward a value of 1 without adequate averaging (smoothing) of the auto and cross-spectral estimates that are used to compute coherence (Figure 1) [28]. Therefore, we used a small segment size of 256 and overlapped the segments by 50% when computing the coherence spectrum with mscohere(). The 95% confidence limit for the threshold of statistically significant coherence is 1 – 0.05^1/(K–1)^, where K is the effective number of segments [23,24,28]. This resulted in 95% coherence thresholds of 0.17 and 0.14 for the two rest conditions and 0.31 for the posture condition.

There is no formula for the 95% confidence threshold for wavelet and Fourier coherence spectrograms [29,30]. We were most interested in determining if there was a significant percentage of each recording in which the coherence was nearly perfect (i.e., 0.8–1.0). We regarded 2–4 Hz as the frequency band of interest, and we specified a coherence of 0.8 as our significance threshold because our null hypothesis was that the head and upper limb oscillations were not synchronous. We randomly shuffled our time series to destroy any time varying relationships between them. We then adjusted the gaussian smoothing window in tfcohf() to consistently achieve a spurious coherence >0.8 less than 5% of the time. These smoothing parameters were then used to compute the percentage of time that coherence was greater than 0.8 in the 2–4 Hz frequency band.

A MATLAB live script file used for all computations is provided in the online supplement. The IMU recordings are provided in comma delimited files that can be analyzed with the MATLAB live script. The MATLAB live script requires the MATLAB Signal Processing, Wavelet, and Statistics and Machine Learning toolboxes (www.mathworks.com).

## Results

The myorhythmia in our patient was primarily rotational motion about the neck and joints of the upper limbs. Gyroscopic transducers measure angular rotation, whereas accelerometers measure translational acceleration [21].Therefore, the results of the gyroscopic recordings are presented here, and the accelerometer data are presented in the online Supplementary Materials. Substantive differences in gyroscopic and accelerometer data are noted in the following.

Tremor frequency ranged from 2.5 to 3.2 Hz, depending on the body part and task (Table 1). Left-hand oscillation had a much lower amplitude than the right and was only intermittently present at rest and at rest while counting backwards (Supplement Figure 6). Tremor was present constantly during active posture and movement (Video).

**Table 1:** Tremor rhythmicity metrics for angular velocity recordings.


RECORDING CONDITION	BODY PART	FREQUENCY (Hz)	MEAN PEAK-TO-PEAK AMPLITUDE (DEGREES)	TREMOR STABILITY INDEX (Hz)	HALF-POWER BANDWIDTH (Hz)	HALF-POWER BANDWIDTH INDEX (Hz)	HARMONIC DISTORTION (ANGULAR VELOCITY)	HARMONIC DISTORTION (ROTATION)

Rest	Head	2.90	2.01	0.09	0.09	0.03	0.12	0.06

	R hand	2.87	14.16	0.11	0.09	0.03	0.29	0.15

	L hand	3.05	6.51	0.15	0.15	0.05	0.17	0.09

Rest counting backwards	Head	2.81	2.02	0.23	0.06	0.02	0.22	0.11

	R hand	2.78	22.56	0.10	0.06	0.02	0.36	0.15

	L hand	3.19	13.37	0.38	0.09	0.03	0.26	0.12

Posture	Head	2.58	13.27	0.15	0.21	0.08	0.11	0.06

	R hand	2.52	60.63	0.13	0.09	0.04	0.32	0.14

	L hand	2.55	27.46	0.36	0.21	0.08	0.35	0.17


**Figure 2:**
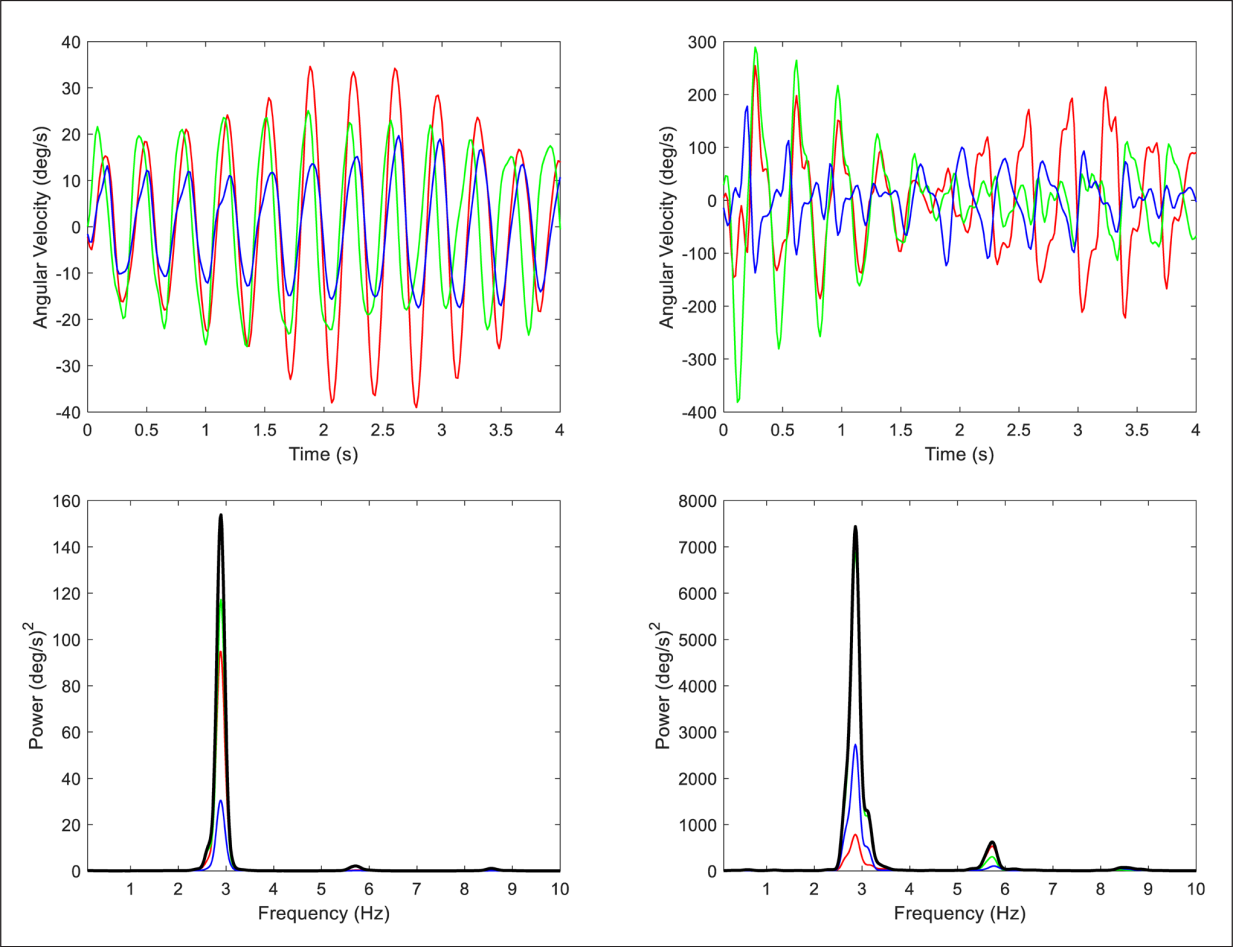
**Gyroscopic recordings and power spectra for the head and right hand**. Gyroscopic recordings (first 4 seconds) and power spectra are shown for the head (left column) and right hand (right column). The patient was asked to relax while lying supine on an exam table. The power spectra contain a large peak at the fundamental frequency (2.89 Hz) and small harmonic peaks at 5.78 Hz and 8.67 Hz. The harmonic peaks in the head spectrum are smaller because the angular velocity waveform is more sinusoidal. The x-y-z axis data are shown in red, green and blue, and the resultant spectral power is shown in black.

The head and hands oscillated very rhythmically. Half-power spectral bandwidth was ≤0.21 Hz, and the Tremor Stability Index was ≤0.38 Hz (Table 1), similar to the values for accelerometry (Supplement Table 1). All power spectra contained harmonics at integer multiples of the fundamental tremor frequency, consistent with the existence of non-sinusoidal waveforms (Figure 2 and Supplement Figure 3). The harmonic distortion in acceleration recordings (mean, SD: 0.46, 0.25) was significantly greater than in gyroscopic recordings (mean, SD: 0.24, 0.10; t = –3.172, df = 8, p = 0.013). However, the difference between harmonic distortion in displacement (mean, SD: 0.09, 0.04) versus rotation (mean, SD: 0.12, 0.04) was only marginally significant and of opposite sign (t = 2.309, df = 8, p = 0.0497) (Table 1 and Supplement Table 1).

**Figure 3:**
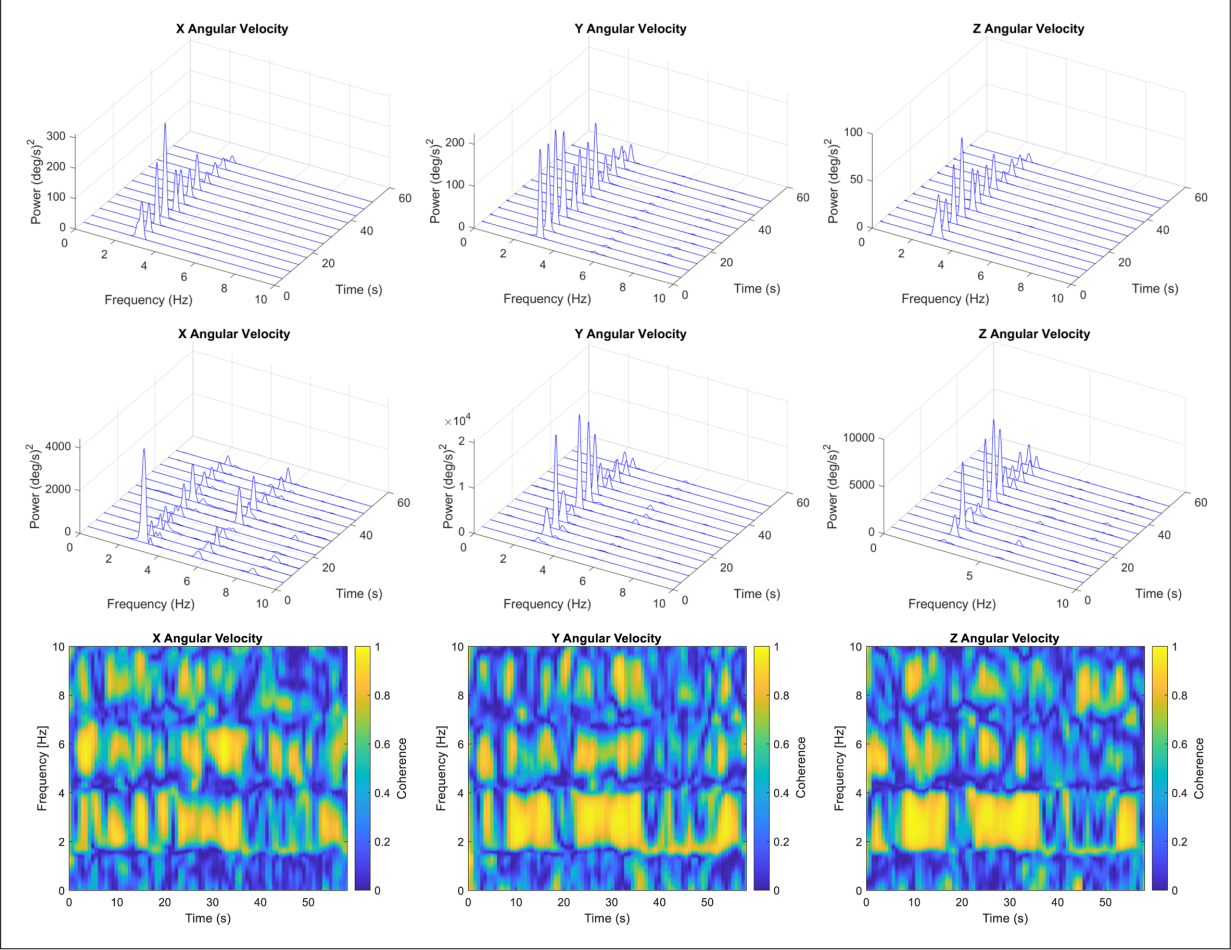
**Time-frequency power spectra and coherence plots of gyroscopic recordings**. Time-frequency waterfall plots of x-y-z spectral power are shown for the head (top row) and right hand (middle row). The patient was asked to relax while lying supine on an exam table. Time-frequency color maps of head vs right hand coherence are shown in the bottom row. Note the considerable variation in peak spectral power over time. Coherence of 0.8–1.0 occurred intermittently at the fundamental frequency (2.89 Hz) and less so at the harmonic frequencies.

The clinical impression of large amplitude fluctuations in head and upper limb oscillation was confirmed by time-frequency spectral analysis (Figure 3) and by visual inspection of the recordings (Supplement Figure 8). The coefficient of variation of cycle-to-cycle peak-to-peak amplitudes ranged from 0.25 to 1.72 and was greatest in the left hand during the two rest conditions (Supplement Table 2).

The head and two hands oscillated at the same frequency at times and at slightly different frequencies at other times. This was particularly true for the left hand during rest and rest while counting backwards (Table 1). Head versus right hand coherence was >0.8 for more than 50% of time in all three recording conditions (Figure 3; Supplement Table 4a). The percentage of time with coherence >0.8 was less between the left hand and the other two body parts in all three recording conditions, particularly the rest while counting condition (Supplement Table 4a).

## Discussion

Tremor is a rhythmic oscillatory involuntary movement [1]. Oscillatory movement is revealed by a statistically significant peak in the Fourier power spectrum of recorded motion [22,31]. The myorhythmia in our patient produced a distinct spectral peak in the range of 2.5–3.2 Hz. The estimated half-power bandwidth ranged from 0.06 to 0.21, and the true half-power bandwidth may be even lower because our estimates were limited by the frequency resolution of our power spectra. Regardless, the half-power bandwidth index was ≤0.08, which means the range of frequencies within the spectral peak was ≤8% of the peak frequency. Similarly, the interquartile range of cycle-to-cycle change in frequency (Tremor Stability Index) was ≤0.23 Hz for the head, ≤0.13 Hz for the right hand, and ≤0.38 Hz for the left hand. These values of frequency variability are comparable to those of the most rhythmic examples of essential tremor and Parkinson tremor [16,32].

The myorhythmia in our patient arguably had a jerky appearance (Video), despite the lack of rhythm variability. We investigated three possible sources of perceived jerkiness: non-sinusoidal waveform, intermittency of oscillation, and fluctuation in tremor amplitude and direction. Other accompanying involuntary movements such as dystonia and myoclonus could produce jerkiness but were not observed in our patient.

When an oscillation is not perfectly sinusoidal, its power spectrum will contain spectral peaks at integer multiples of the fundamental frequency. These higher harmonics are characteristic of nonlinear systems [33]. Nonlinearity is a universal property of the nervous system, so deviation from sinusoidality will exist in most tremor recordings [31]. In this study, deviation from a sinusoidal waveform was quantified by the total harmonic distortion, and the values in our patient ranged from 0.11 to 0.36 for gyroscopic recordings and 0.19 to 0.90 for accelerometry. Accelerometers measure translational acceleration, and they may introduce artifactual harmonic distortion when they are subjected to rotational motion [34]. Also, acceleration is proportional to hand displacement times the squared frequency, and angular velocity is proportional to hand rotation times frequency [21]. Therefore, harmonics will be amplified in acceleration and angular velocity recordings, compared to displacement and rotation, and amplification will be greatest in acceleration (Supplement Figure 7). These factors must be considered when comparing harmonic distortion using different transducers. Artificial sine wave, triangular wave, square wave, and sawtooth wave oscillations have total harmonic distortion values of 0.0, 0.121, 0.483, and 0.803, respectively [20]. Harmonic distortion in hand and head rotation varied between 0.06–0.17. Therefore, the myorhythmia in our patient was clearly not sinusoidal, but the ability of a clinician to perceive this degree of harmonic distortion at frequencies >2 Hz seems doubtful and has not been studied.

Time-frequency spectral analysis revealed large fluctuations in amplitude (spectral power) and direction (Figure 3). These fluctuations were also evident in the unprocessed recordings (Supplement Figure 8). Low-frequency (<1 Hz) amplitude fluctuations are easily perceived by clinical inspection and probably contribute to the impression of jerkiness.

Myorhythmia is usually associated with damage to the Guillain-Mollaret triangle and homologous mesodiencephalic triangles [2,35]. Myorhythmia in this context is probably pathophysiologically different from the myorhythmic movements associated with dystonia, following posterolateral thalamic stroke [9,36]. The slow repetitive movements in dystonia may or may not be rhythmic enough to be considered a form of tremor [37]. The demyelination of our patient’s brainstem and cerebellum involved all three arms of the Guillain-Mollaret triangle.

A highly rhythmic oscillation produces a sharp fundamental spectral peak, meaning most of its energy is concentrated around a specific frequency. Less rhythmic oscillations have spectral power that is spread across a wider range of frequencies [31]. It is unlikely that any tremor is perfectly rhythmic because of inevitable noise and incomplete entrainment in the underlying neural networks. Terms such as “pseudorhythmic” and “semirhythmic” are used by clinicians to describe repetitive movements that appear to lack sufficient rhythmicity to warrant the classification of tremor, but these terms lack operational definitions, and agreement among clinicians is poor [33].

In 1997, an international conference of tremor researchers recommended that oculopalatal myoclonus be classified as a tremor [38]. The ocular and palatal tremors produce a prominent spectral peak in the frequency range of 1–3 Hz [39], so oculopalatal tremor is arguably a form of myorhythmia, as currently defined [1,4]. The imperfect rhythmicity and non-sinusoidal waveform of palatal tremor can be appreciated by clinical inspection due to the low frequency of oscillation. One kinematic study revealed a half-power bandwidth of 0.69 Hz and a half-power bandwidth index of 0.37 [31], values that are much larger than those found in the present study. The half-power bandwidth index is more useful than the raw bandwidth when comparing different tremor types because the index is a dimensionless fraction of the peak tremor frequency. For example, primary orthostatic tremor is believed to be the most rhythmic form of tremor [22], and one published example revealed a half-power bandwidth of 0.4 Hz but an index of only 0.027 [31].

Myorhythmia has been variably described as being asynchronous or synchronous among different body parts, but this had not been determined quantitatively [2,40]. We demonstrated intermittently strong (>0.8) coherence among the head and hands of our patient, consistent with the presence of synchrony. Synchronous oscillation among affected body parts is a well-known characteristic of primary orthostatic tremor [41]. It is also a stated characteristic of oculopalatal tremor [42,43], but there is no published report of coherence analysis. Synchronous bilateral tremor in multiple body parts suggests a very strong entrainment of multiple oscillators or a single source of oscillation with widespread bilateral anatomical connections, such as the cerebellar fastigial nucleus [44,45,46]. Additional clinicopathological correlation with quantitative electrophysiology is crucial to a better understanding of these phenomena.

We did not record EMG, and this is a limitation of our study. The use of motion sensors to examine coherence among ipsilateral and contralateral body parts could be confounded by the mechanical transmission of tremor from one body part to another, and EMG would have provided a control for possible mechanical transmission. Tremor in her left hand was only intermittently visible during the two rest conditions, despite ongoing tremor in the head and right hand (Supplemental Figure 8). Therefore, tremor in one location was not simply passively transmitted to the other locations. Tremor was severe and constant during the postural task, yet the coherence among body parts was comparable to that in the quiet rest condition (Supplemental Table 4a). Therefore, passive mechanical transmission of tremor made no apparent contribution to our coherence estimates, and we used a conservative coherence threshold of 0.8 for testing our hypothesis of synchrony between body parts.

EMG recordings have limitations. EMG recordings of tremor are far more noisy than IMU recordings, and adequate muscle sampling is difficult. Our IMU recordings had no measurable extraneous noise, which allowed us to use brief recordings. Lower signal to noise ratios require longer recordings and greater spectral averaging to detect tremor and coherence, as in EEG and EMG tremor studies [22,24].

Published EMGs of myorhythmia contained burst durations that were commensurate with the tremor frequency but varied significantly from cycle to cycle [3,5,10,12,13]. A relationship between tremor frequency and EMG burst duration was reported previously and is expected [47,48]. Higher frequency tremors, by necessity, have shorter EMG bursts. For example, you cannot have a 10 Hz tremor (tremor period = 100 msec) with 200–300 msec bursts of EMG. Similarly, the EMG bursts in 13–18 Hz orthostatic tremor are necessarily very brief. Ditto for high-frequency rhythmic cortical myoclonus or cortical tremor [48]. The amplitude modulation of EMG in all tremor disorders is not sinusoidal, but the impact of this on tremor kinematics is very small due to the low-pass filtering (smoothing) properties of muscle contraction and musculoskeletal mechanics [22,49].

In conclusion, our patient’s myorhythmia was exceptionally rhythmic and is justifiably classified as a tremor. Intermittently strong 0.8–1.0 coherence between body parts was also evident, proving that myorhythmia can be intermittently synchronous between different body parts. The methods of analysis in this study have computational details that must be carefully considered to achieve valid reliable results. Psychophysical studies are needed to define the kinematic characteristics of tremor that produce the human perception of jerkiness, but large low-frequency fluctuations in tremor amplitude seem more likely to produce the impression of jerkiness than rhythm variability or waveform distortion at the frequency of tremor.

## Additional Files

The additional files for this article can be found as follows:

10.5334/tohm.986.s1 Supplementary File 1. Supplementary figures and tables.

10.5334/tohm.986.s2 Supplementary File 2. MATLAB live script with code for all analyses in Microsoft Word.

10.5334/tohm.986.s3 Supplementary File 3. Zip folder containing compressed files of transducer recordings and MATLAB live script with code for all analyses.
