# Supplementary material for: Myorhythmia: A Quantitative Study of Synchrony and Rhythmicity Between the Head and Upper Limbs
Source: Tremor Other Hyperkinet Mov (N Y). 2025 Apr 1;15:9. doi: 10.5334/tohm.986 (PMC11967460; doi:10.5334/tohm.986)
Supplement: Supplementary File 1. — Supplementary figures and tables. [file tohm-15-1-986-s1.pdf]

Supplemental Materials

Figure 1a: T2 flair MRI performed in February 2024

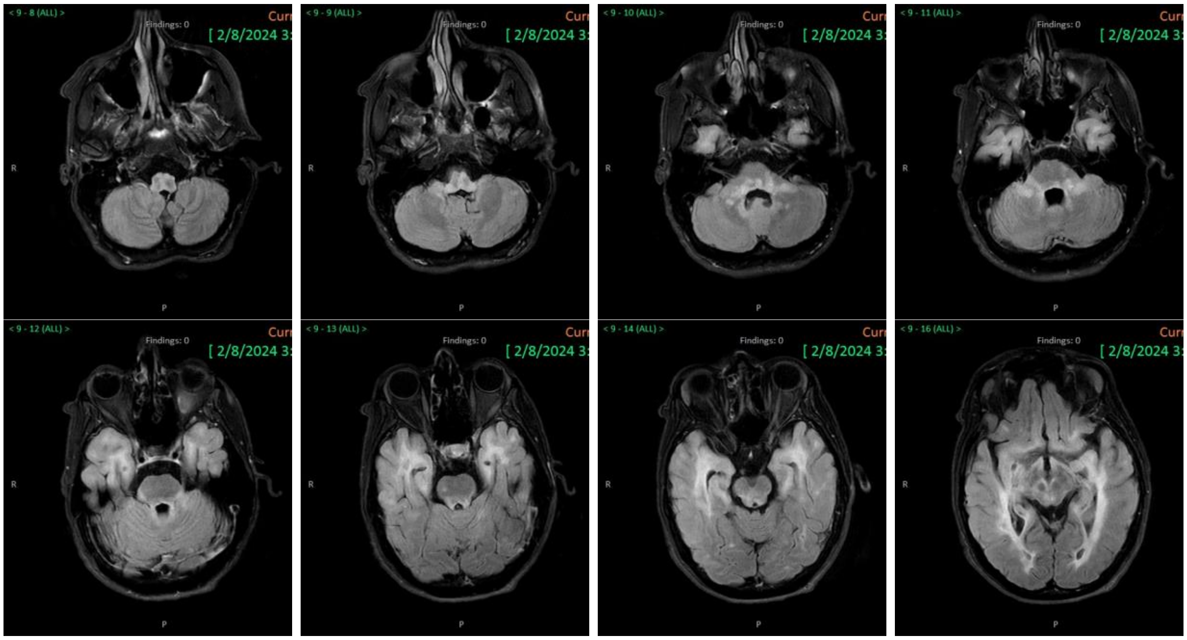

Figure 1b: T2 flair MRI performed in October 2022

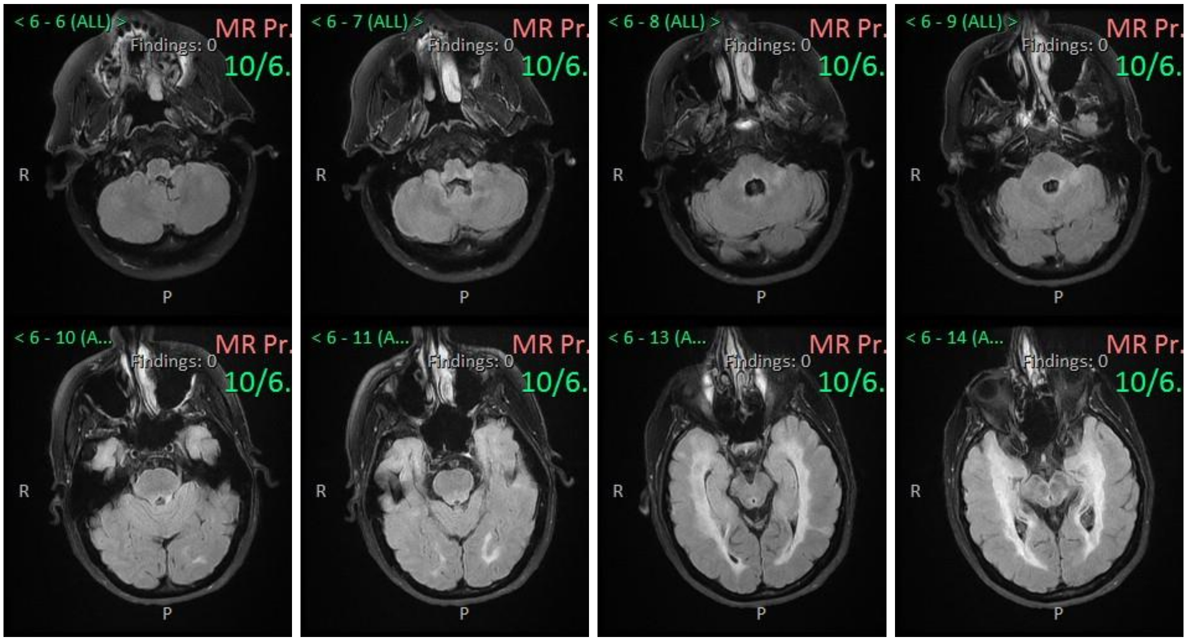

The February 2024 scan revealed much more extensive demyelination in the medulla, pons, midbrain, all cerebellar peduncles, and the deep cerebellar white matter.

Figure 2: Locations and orientations of the Xsens DOT IMUs

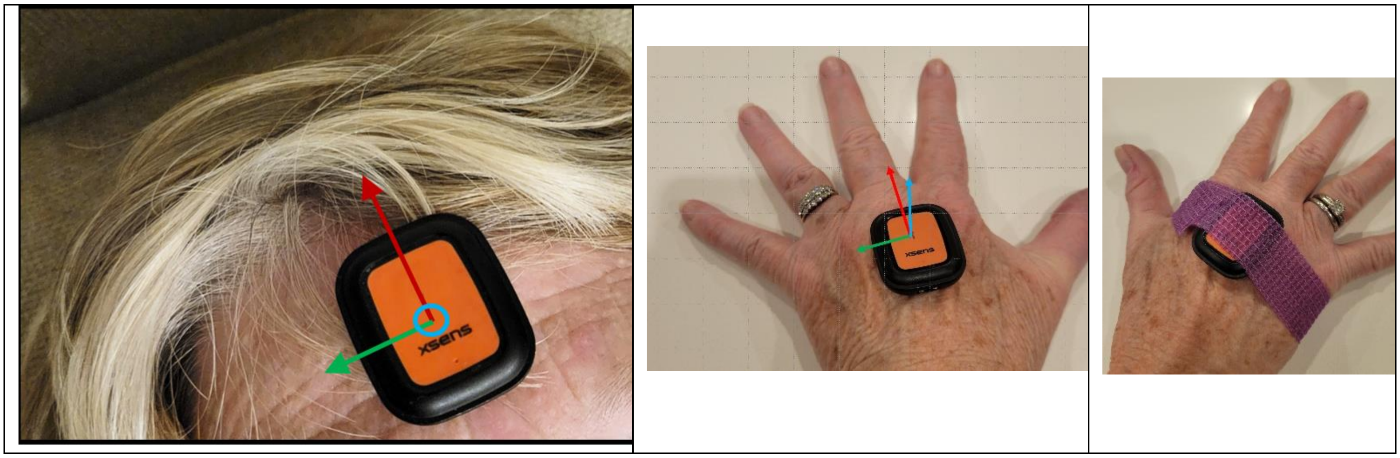

These photos reveal the location and orientation of the IMU on the forehead and two hands. The x-y-z axes are colors red, green and blue. The z axis is perpendicular to the IMU. At each location, the IMU was secured with elastic self-adhesive tape wrapped around the hands and head.

Figure 3: Accelerometry recordings and power spectra for the head and right hand

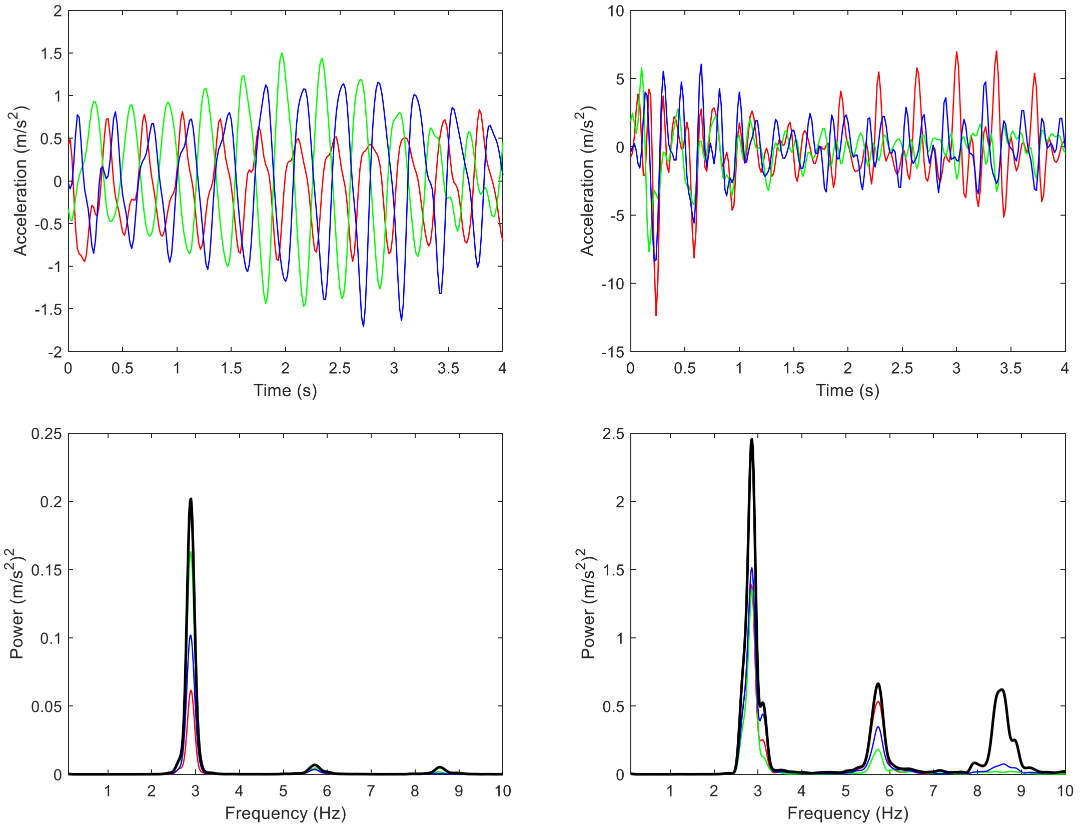

Accelerometry recordings (first 4 seconds) and power spectra are shown for head (left column) and right hand (right column). The patient was asked to relax while supine on an exam table. The x, y and z axis data are shown in red, green and blue. The waveforms of the recordings contain harmonic oscillations at 5.78 and 8.67 Hz, more so in the hand. The spectral peaks at the fundamental frequency (2.89 Hz) have very narrow half-power bandwidths (Welch segment size = 512 samples; frequency resolution = 60/512 = 0.117 Hz) . The resultant x-y-z spectral power is shown in black.

Figure 4: Time-frequency coherence spectrograms with and without data randomization.

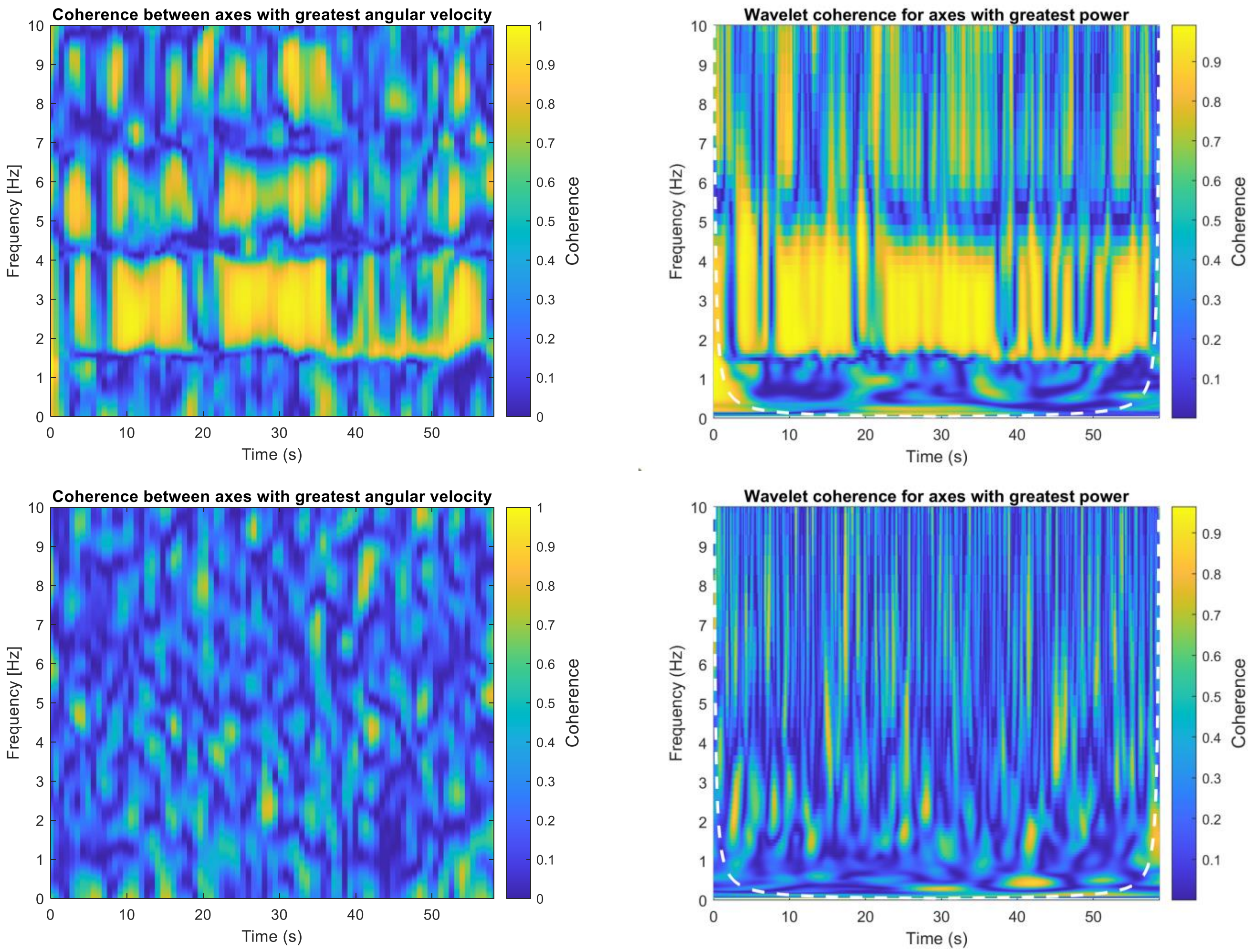

Fourier (left column) and wavelet (right column) time-frequency coherence spectrograms are shown for the head versus right hand angular velocity. The patient was quietly at rest on an examination table. Coherence spectrograms of randomly shuffled time series are shown in the bottom row. Note that the spectrograms of shuffled data still contain foci of spurious coherence. The percentage of total time that coherence in the 2-4 Hz band exceeded 0.8 was 51% using the Fourier method and 67% using the wavelet method. After random shuffling, the time fractions were 0% and 3%, respectively. The Fourier method appears to do a better job of resolving harmonics, but this was not studied systematically.

Figure 5: Time-frequency power spectra and coherence plots of accelerometry recordings

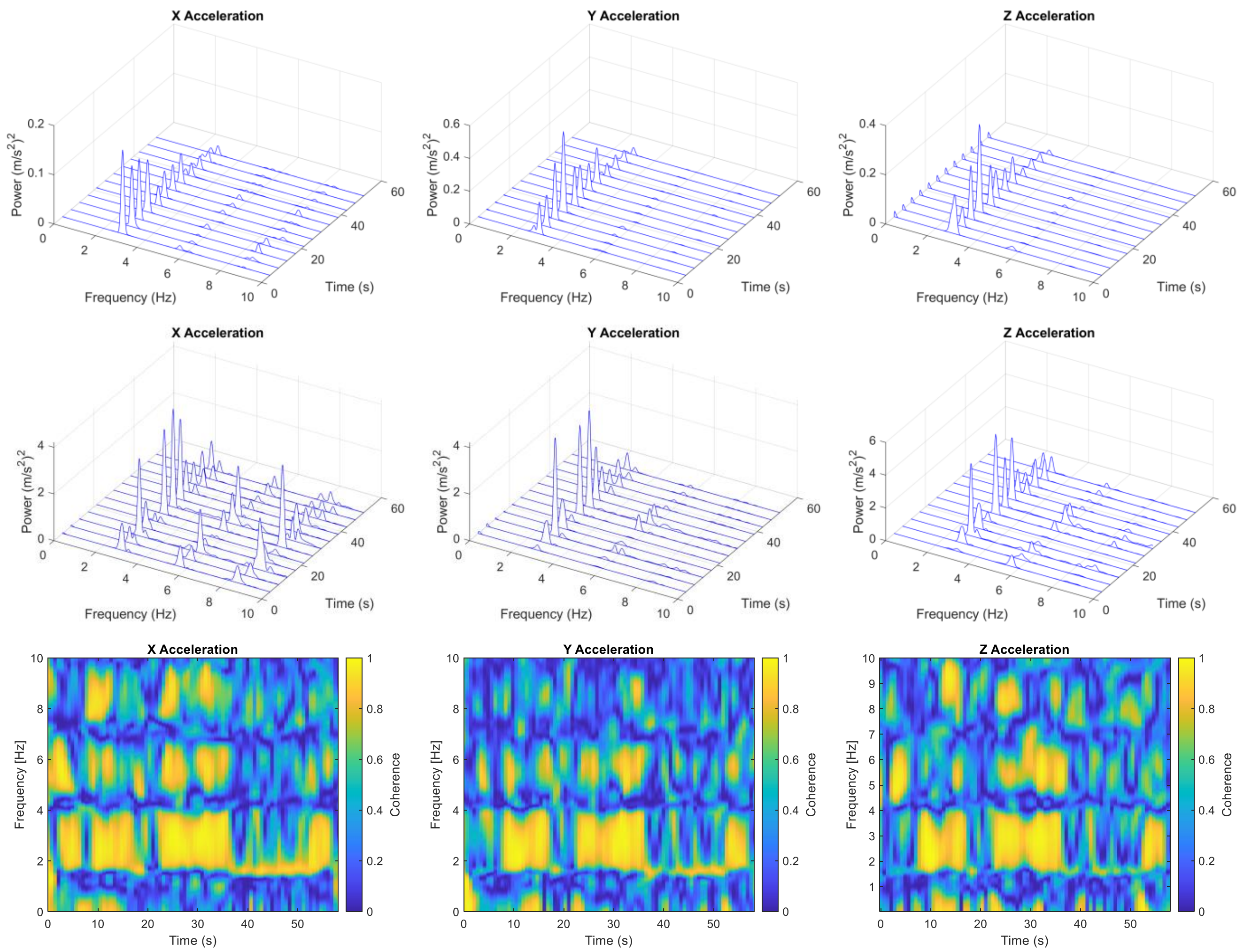

Time-frequency waterfall plots of x-y-z spectral power are shown for the head (top row) and right hand (middle row). The patient was relaxing quietly while supine on an exam table. Time-frequency Fourier spectrograms of head vs right hand coherence are shown in the bottom row. Coherence of 0.8-1.0 occurred intermittently at the fundamental frequency (2.89 Hz) and less so at the harmonic frequencies (5.78 Hz and 8.67 Hz).

Figure 6: Gyroscopic recordings of the left hand

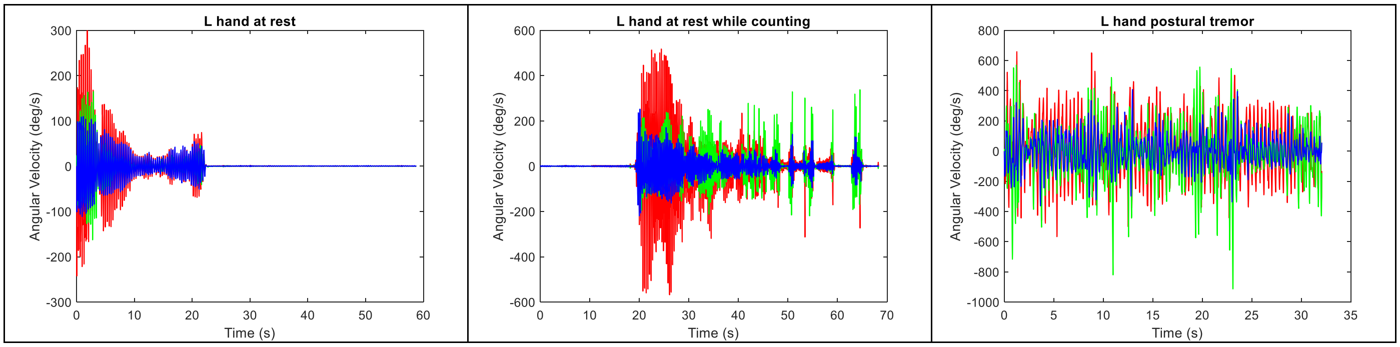

The x-y-z (red-green-blue) angular velocity recordings were recorded from the left hand at rest, rest while counting, and in posture. The tremor abruptly stopped after 22 s at rest. Tremor was absent during the first 18 s of the rest while counting recording and was only intermittently present after 45 s. Continuous tremor was recorded in the postural task.

Figure 7a: Rotation time series of head (left column) and right hand (right column).

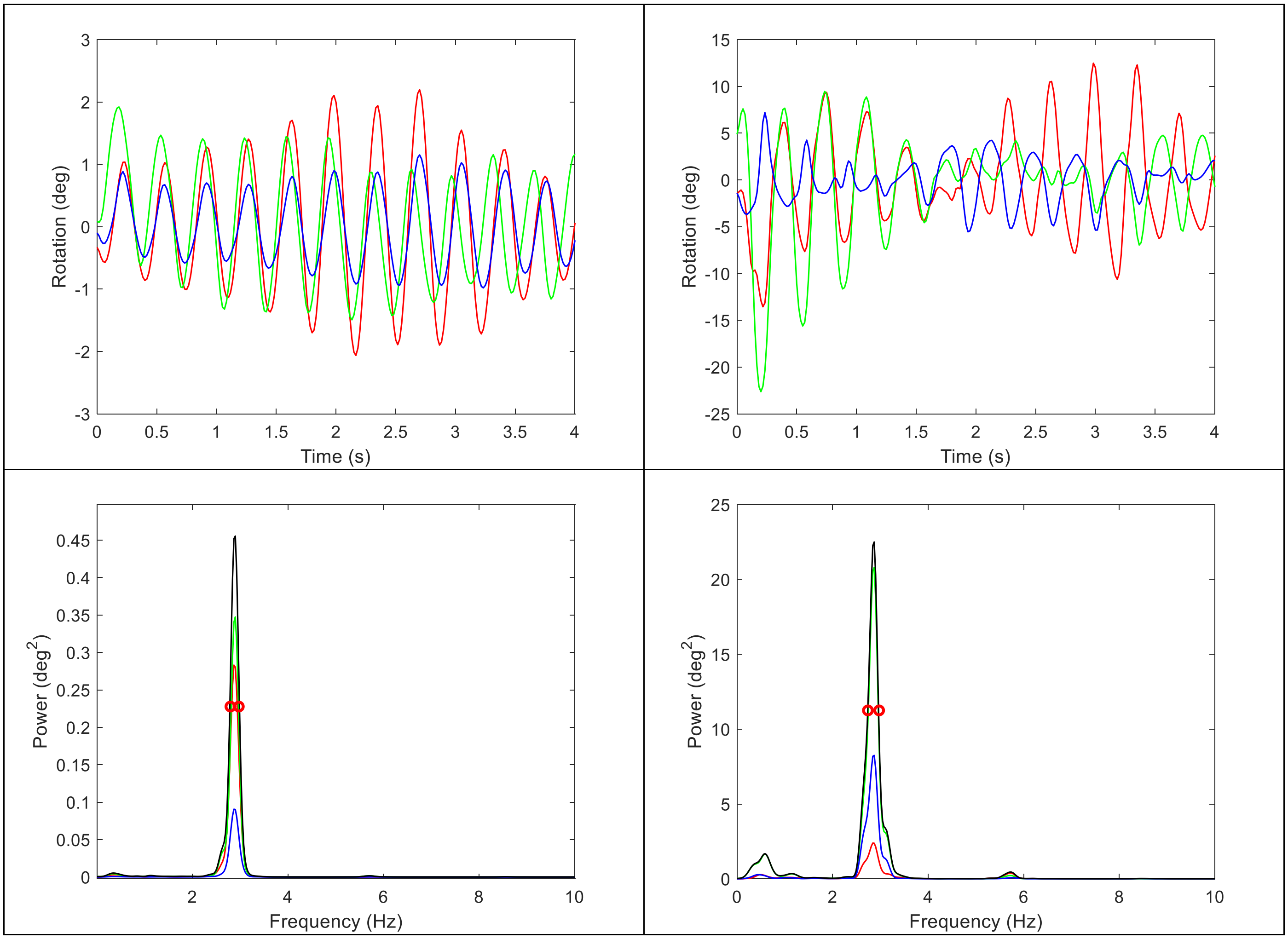

Figure 7b: Displacement time series of head (left column) and right hand (right column).

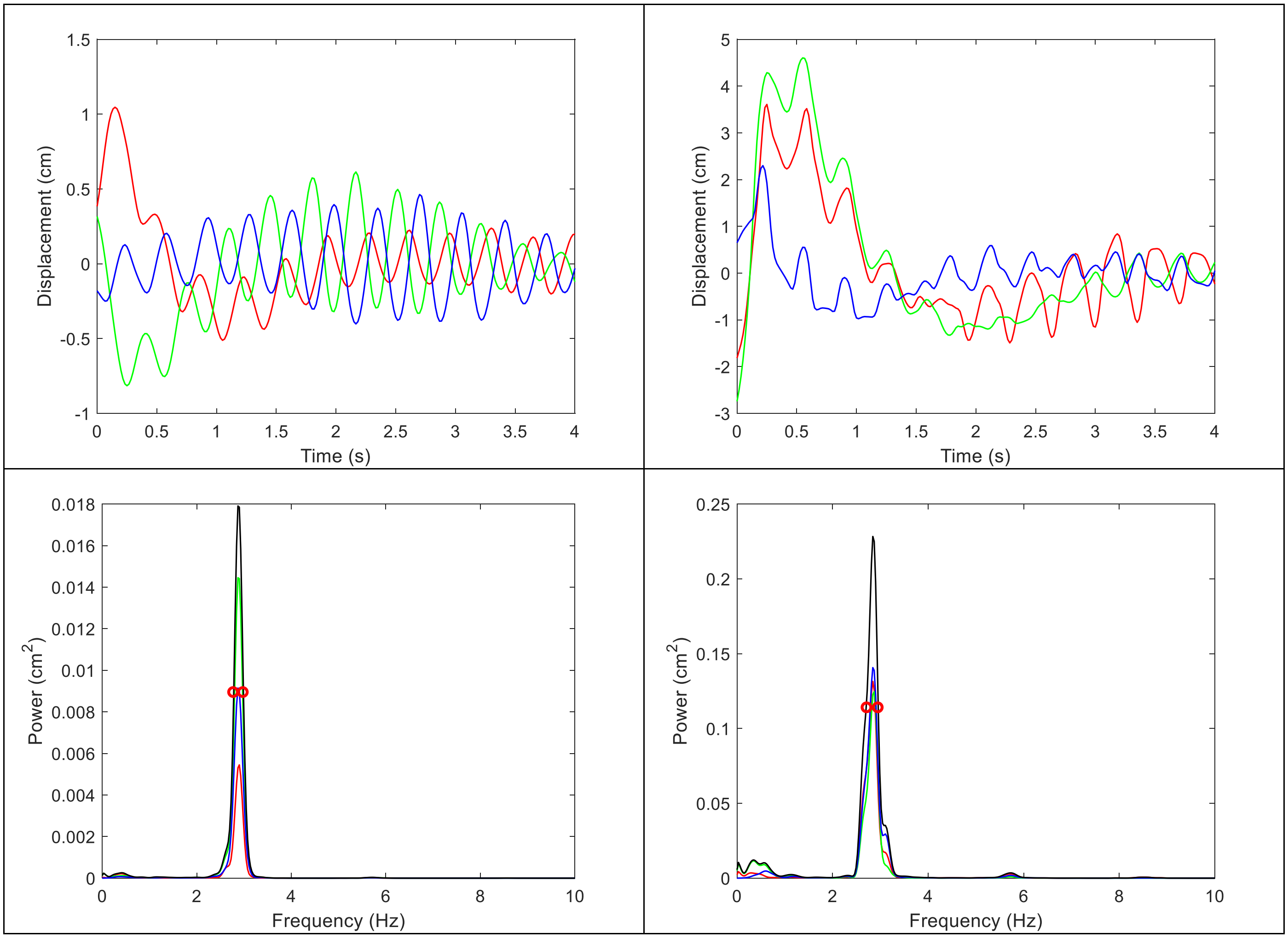

The x-y-z (red-green-blue) rotation and displacement time series were mathematically derived from angular velocity and acceleration recordings using numerical integration (MATLAB cumtrapz()). Patient was at rest on an examination table. Compare the plots in 7b with those in Supplement Figure 3. Note that the displacement time series are much more sinusoidal than the acceleration recordings, and the higher harmonics at 5.8 Hz and 8.7 Hz are barely visible in the displacement power spectra. Compare Figure 7a with Figure 2 in the manuscript to see how harmonic distortion is less in rotation compared to angular velocity. Welch segment size = 512 samples.

Figure 8: Angular velocity recordings

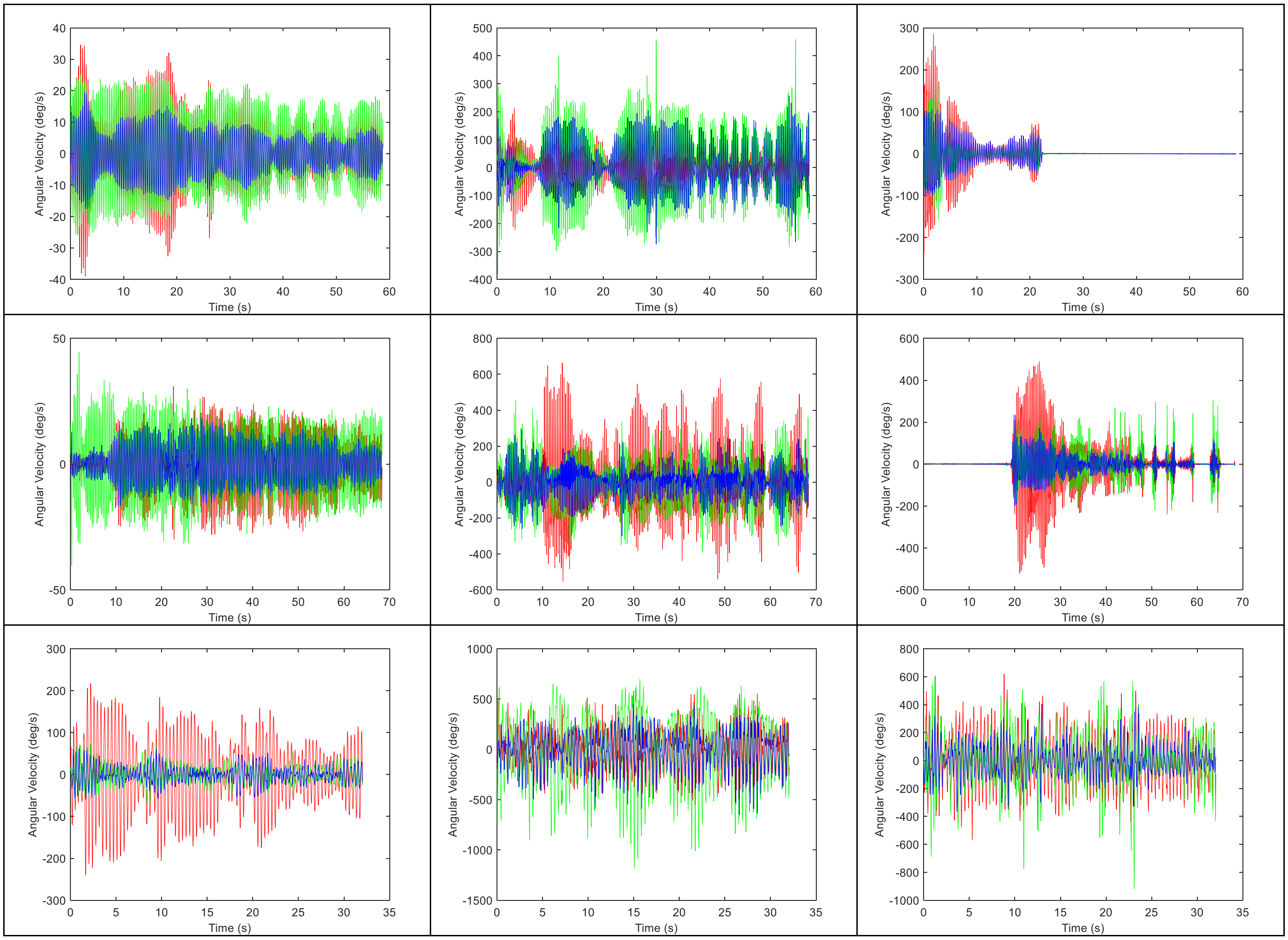

The x-y-z (red-green-blue) angular velocity recordings from the head (left column), right hand (middle column) and left hand (right column) are shown for the three recording conditions of rest (top row), rest while counting backwards (middle row) and posture (bottom row). Marked fluctuations in amplitude and direction are present in all recordings, and tremor in the left hand was present only intermittently during the two rest conditions.

Table 1: Tremor rhythmicity metrics for accelerometry recordings

Recording condition	Body part	Frequency (Hz)	Mean peak-to-peak displace ment (cm)	Tremor Stability Index (Hz)	Half-power bandwidth (Hz)	Half-power bandwidth index	Harmonic distortion (accelera-tion)	Harmonic distortion (displace-ment)
Rest	Head	2.90	0.40	0.09	0.09	0.03	0.25	0.05
	R hand	2.84	1.45	0.14	0.09	0.03	0.76	0.14
	L hand	2.87	0.83	0.10	0.15	0.05	0.37	0.08
Rest counting backwards	Head	2.81	0.46	0.23	0.06	0.02	0.37	0.08
	R hand	2.78	2.57	0.18	0.09	0.03	0.90	0.16
	L hand	3.16	1.15	0.33	0.09	0.03	0.65	0.11
Posture	Head	2.58	4.67	0.11	0.21	0.08	0.19	0.05
	R hand	2.52	17.52	0.06	0.09	0.04	0.31	0.06
	L hand	2.58	8.37	0.15	0.21	0.08	0.34	0.08

Table 2: Cycle-to-cycle peak-to-peak amplitude variation of the first principal component of triaxial gyroscope and accelerometer recordings

Gyroscopic data

Recording condition	Body part	Inter-quartile range (deg/s)	Mean amplitude (deg/s)	SD	CV
Rest	Head	19.3	45.2	14.0	0.31
	R hand	268.4	315.6	168.7	0.55
	L hand	70.9	53.5	91.8	1.72
Rest counting backwards	Head	13.3	44.4	11.1	0.25
	R hand	382.7	369.7	234.1	0.63
	L hand	240.5	170.2	213.8	1.26
Posture	Head	162.6	219.2	96.9	0.44
	R hand	435.6	987.2	289.0	0.29
	L hand	272.7	555.1	233.2	0.42

Accelerometer data

Recording condition	Body part	Inter-quartile range (m/s^2)	Mean amplitude (m/s^2)	SD	CV
Rest	Head	0.80	1.63	0.62	0.38
	R hand	5.82	6.47	3.59	0.56
	L hand	1.56	1.10	1.94	1.77
Rest counting backwards	Head	0.65	1.60	0.52	0.33
	R hand	7.90	7.85	4.61	0.59
	L hand	4.51	2.84	3.20	1.13
Posture	Head	10.39	13.81	5.89	0.43
	R hand	6.47	45.58	5.10	0.11
	L hand	12.18	24.36	8.92	0.37

Inter-quartile range: cycle-to-cycle peak-to-peak amplitudes

SD: standard deviation of the peak-to-peak amplitudes of each cycle of oscillation

CV: coefficient of variation (SD/mean)

Table 3a: Angular velocity coherence at the tremor frequency

		Fourier coherence at the peak frequency of tremor							
Task	Body parts	x-x	y-y	z-z	Axis1-Axis2	Axis1	Axis2	95%CL	99%CL
rest	Head vs R hand	0.48	0.57	0.36	0.57	y	y	0.17	0.25
	Head vs L hand	0.34	0.11	0.34	0.06	y	x	0.17	0.25
	R hand vs L hand	0.30	0.07	0.08	0.05	y	x	0.17	0.25
rest counting	Head vs R hand	0.73	0.35	0.21	0.45	y	x	0.14	0.21
	Head vs L hand	0.11	0.15	0.43	0.16	y	x	0.14	0.21
	R hand vs L hand	0.15	0.16	0.13	0.15	x	x	0.14	0.21
posture	Head vs R hand	0.66	0.68	0.30	0.50	x	y	0.31	0.44
	Head vs L hand	0.84	0.36	0.66	0.84	x	x	0.31	0.44
	R hand vs L hand	0.65	0.28	0.60	0.59	y	x	0.31	0.44

Table 3b: Acceleration coherence at the tremor frequency

		Fourier coherence at the peak frequency of tremor							
Task	Body parts	x-x	y-y	z-z	Axis1-Axis2	Axis1	Axis2	95%CL	99%CL
rest	Head vs R hand	0.68	0.50	0.38	0.43	y	z	0.17	0.25
	Head vs L hand	0.11	0.28	0.31	0.28	y	y	0.17	0.25
	R hand vs L hand	0.05	0.08	0.17	0.06	z	y	0.17	0.25
rest counting	Head vs R hand	0.38	0.16	0.24	0.61	z	x	0.14	0.21
	Head vs L hand	0.30	0.29	0.16	0.14	z	y	0.14	0.21
	R hand vs L hand	0.11	0.18	0.19	0.23	x	y	0.14	0.21
posture	Head vs R hand	0.30	0.70	0.28	0.70	y	y	0.31	0.44
	Head vs L hand	0.70	0.84	0.40	0.84	y	y	0.31	0.44
	R hand vs L hand	0.24	0.66	0.38	0.66	y	y	0.31	0.44

Angular velocity coherence between the corresponding axes of IMU1 and IMU2 is shown in Table 3b for each combination of body parts and for each task. The results for acceleration recordings are shown in Table 3b. Axis1 and Axis2 are the axes of IMU1 and IMU2 with greatest tremor power. The coherence spectrum was computed with MATLAB mscohere(), which provided the frequency distribution of what is effectively the average coherence at each frequency. 95%CL and 99%CL are the 95% and 99% thresholds for statistically significant coherence. Analysis with mscohere() assumes that the time series are statistically stationary, and the time series in this study were not stationary. Non-stationarity has the effect of lowering the coherence estimates, which are reported here for information only. Our hypothesis of intermittent synchrony was tested with time-frequency coherence analyses, summarized in Table 4.

Table 4a: Percentages of total time with angular velocity coherence > 0.8

		Fourier coherence spectrogram				Wavelet coherence spectrogram				Axes with greatest tremor	
Task	Body parts	x-x	y-y	z-z	Axis1-Axis2	x-x	y-y	z-z	Axis1-Axis2	Axis1	Axis2
rest	Head vs R hand	40	51	52	51	63	67	67	67	y	y
	Head vs L hand	39	30	49	42	53	56	56	57	y	x
	R hand vs L hand	37	35	62	36	49	46	46	50	y	x
rest counting	Head vs R hand	54	55	44	53	63	71	71	64	y	x
	Head vs L hand	14	12	22	19	15	19	19	23	y	x
	R hand vs L hand	10	9	10	10	14	19	19	14	x	x
posture	Head vs R hand	54	57	20	67	54	64	64	77	x	y
	Head vs L hand	67	16	15	67	64	17	17	64	x	x
	R hand vs L hand	39	26	39	67	42	27	27	71	y	x

Table 4b: Percentages of total time with acceleration coherence > 0.8

		Fourier coherence spectrogram				Wavelet coherence spectrogram				Axes with greatest tremor	
Task	Body parts	x-x	y-y	z-z	Axis1-Axis2	x-x	y-y	z-z	Axis1-Axis2	Axis1	Axis2
rest	Head vs R hand	56	49	46	51	71	62	59	67	y	y
	Head vs L hand	68	51	30	51	75	73	36	73	y	y
	R hand vs L hand	48	73	18	62	51	78	28	66	z	y
rest counting	Head vs R hand	50	31	41	57	67	51	54	68	z	x
	Head vs L hand	9	17	20	18	18	29	27	23	z	y
	R hand vs L hand	10	9	10	10	12	25	26	22	x	y
posture	Head vs R hand	28	85	63	85	41	86	74	86	y	y
	Head vs L hand	70	76	24	76	78	77	37	77	y	y
	R hand vs L hand	28	78	20	78	29	85	34	85	y	y

For each combination of body parts and recording condition, the percentages of time in which IMU1 vs IMU2 coherence in the 2-4 Hz band was >0.8 were calculated for each pair of x-y-z axes and for the IMU1 and IMU2 axes with greatest tremor. The Fourier coherence spectrograms were computed with MATLAB tfcohf(), using parameters that resulted in less than 5% of time with coherence >0.8 when the time series were shuffled randomly in time. The results for angular velocity are shown in Table 4a and for acceleration in Table 4b.
