# Supplementary material for: Myorhythmia: A Quantitative Study of Synchrony and Rhythmicity Between the Head and Upper Limbs
Source: Tremor Other Hyperkinet Mov (N Y). 2025 Apr 1;15:9. doi: 10.5334/tohm.986 (PMC11967460; doi:10.5334/tohm.986)
Supplement: Supplementary File 2. — MATLAB live script with code for all analyses in Microsoft Word. [file tohm-15-1-986-s2.pdf]

Analyze two Xsens DOT transducers

Author: Rodger Elble **Date:** 13Feb2025

Objective: Analyze two simultaneously-recorded Xsens DOT inertial measurement units (IMU).

Notes to Users:

1. This live script was written using MATLAB 2024b and its Signal Processing, Wavelet, and Statistics and Machine Learning toolboxes (www.mathworks.com). Time-frequency coherence was computed with tfcohf(), which can be downloaded at www.mathworks.com/matlabcentral/fileexchange/38537-time-frequency-coherency
2. This live script was designed for the analysis of specific data from a woman with myorhythmia affecting her head and upper limbs. Some parameters of the analyses may need to be adjusted for other data.
3. The user is given the option of trimming time (data) from the beginning and end of recordings. The default is 0s (no trim) from the beginning or end.
4. The user is also given the option of randomly shuffling the accelerometer and gyroscope recordings to observe the effect of randomizing the samples in time (a type of Monte Carlo analysis). The default is 0 (no shuffling). Answer 1 (yes) if randomization is desired, and then specify the block size for shuffling. A block size of 1 results in complete data shuffling that destroys the original time structure, converting each time series into "white noise". A block size equivalent to one tremor cycle (60 samples/sec / 3 cycles/sec = 20 samples per tremor cycle) or two tremor cycles (40 samples) will retain some oscillatory structure in the shuffled time series, so the power spectra of the time series will contain a noisy spectral peak at the tremor frequency. However, any relationship between the timing of oscillations in the two time series is lost, and any remaining coherence is statistically spurious.
5. The Xsens DOT sampling frequency is 60 samples per second. The time resolution is therefore 1/60 = 0.0167s. When computing cycle-to-cycle frequency variability, spline interpolation is used to increase the time resolution to 0.001s (interpolation frequency = 1000 samples/s). Interpolation is needed to compute precise axis crossings.
6. The power spectra (autospectra) are computed to determine the frequency and amplitude of the fundamental peak and its higher harmonics. The power spectrum is used to compute harmonic distortion (a measure of nonsinusoidality of waveform) and half-peak power bandwidth of the fundamental peak, a measure of frequency variability. For these measures, the greatest frequency resolution compatible with an accurate estimate of fundamental peak and harmonic spectral power is desired because we want our estimate of half-power bandwidth to be a measure of frequency variability, not limited by frequency resolution. The maximum frequency resolution is 1 / (duration of time series recording). In other words, maximum frequency resolution is achieved by analyzing the entire recording as one segment, but the spectral amplitudes will be very noisy. The postural task in this study was recorded for approximately 30s, compared to 60s for the two recordings at rest (rest2 with and Rest1 without counting backwards from 100). Therefore, the maximum frequency resolution of the postural task is half that of the two recordings at rest.
7. The power spectra are computed with the Welch method using the MATLAB pwelch() function. This method divides the time series into *n* segments of size *L* samples = number of samples in time series / *L*. The segments are zero padded to a number specified by *nfft = 2048*, and the fast Fourier transform of each segment is computed. The *n* spectra are then averaged to obtain statistically reliable spectral estimates. The segment size used in pwelch is specified on line 106.
8. The frequency resolution of the power spectra is determined by the length *L* of the segments used by the Welch Fourier power spectrum and coherency spectrum routines. For example, when *L* = 512 samples, the duration of *L* is 512 / 60 = 8.533 s, resulting in a frequency resolution of 1 / 8.533 = 0.117 Hz. This frequency resolution will limit the resolution of half-peak power bandwidth, making the bandwidth finite even when there is no noise or variability in tremor frequency. Increasing *L* increases the frequency resolution of the power spectrum, but it reduces the number of segments that can be averaged, resulting in greater noise (uncertainty) in each power spectral amplitude estimate.

9. The Fourier coherence spectra are computed with the main objective of detecting statistically reliable coherence between two time series. Frequency resolution is usually less important in coherence analysis. Therefore, we used segments that were 256 samples long and overlapped the segments by 50% when computing coherence with mscohere(). The Fourier time-frequency coherence spectrogram was computed with tfcohf() using segment size = 1/16 the length of the time series and 83% (5/6) overlap.
10. The average coherence is computed with mscoher(). The "average coherence" computed from the tfcohf() spectrograms is not mathematically legitimate and is for comparison only.
11. The tfcohf() uses a gaussian kernel to smooth the spectrogram. The values used in this program were optimal for the data being analyzed.
12. Wavelet coherence spectrograms were computed to verify the results of tfcohf().
13. Plots of x-y-z data are color-coded red-green-blue.

Load the Xsens DOT *.csv data files into arrays named data1 and data2.

```
**PARAMETERS OF ANALYSES IN THE TIME AND FREQUENCY DOMAINS**
Sampling frequency: 60 samples/s
Sampling interval (s): 0.0167 seconds
Total number of samples in each time series: 3525 samples
Duration of each time series: 58.75 seconds
Maximum possible frequency resolution: 0.0170 Hz
Welch power spectral segment size: 512 samples
Welch power spectral frequency resolution: 0.1172 Hz
Welch coherence segment size: 256 samples
Welch coherence frequency resolution: 0.2344 Hz
```

X-Y-Z ACCELERATION TIME SERIES STATISTICS FOR IMU #1

Now plot the entire recording and first 4 seconds of x,y,z acceleration for IMU #1.

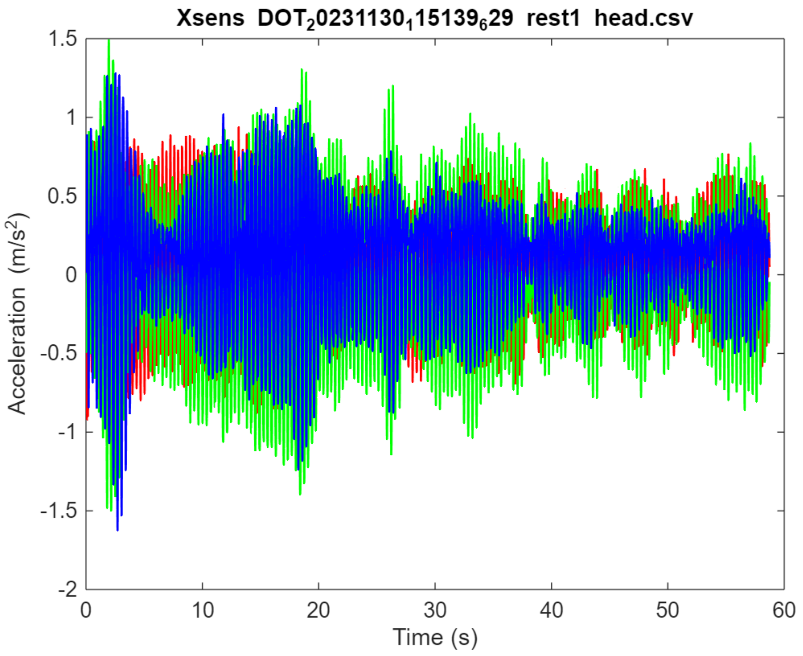

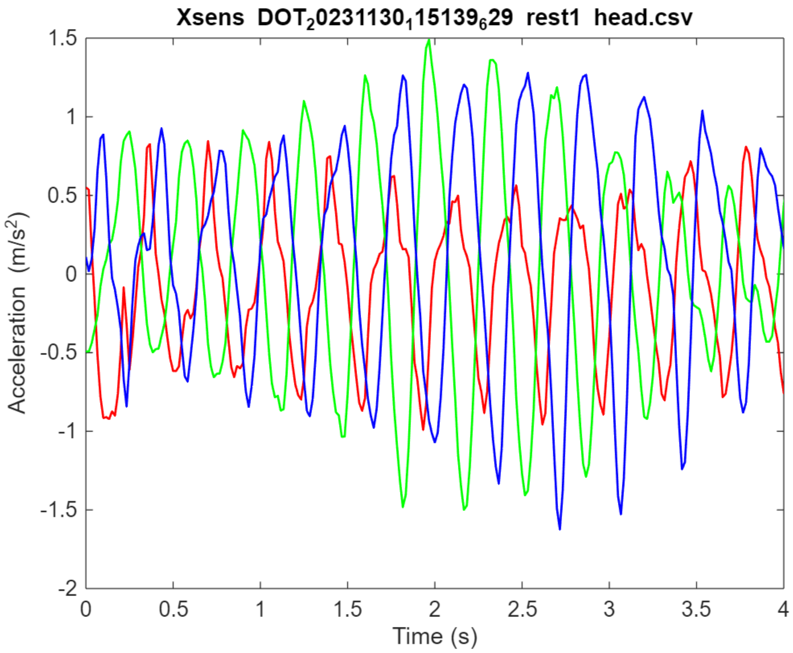
Xsens DOT20231130115139629 rest1 head.csv
Acceleration (m/s2)
Time (s)
1.5
1
0.5
0
-0.5
-1
-1.5
-2
0
0.5
1
1.5
2
2.5
3
3.5
4

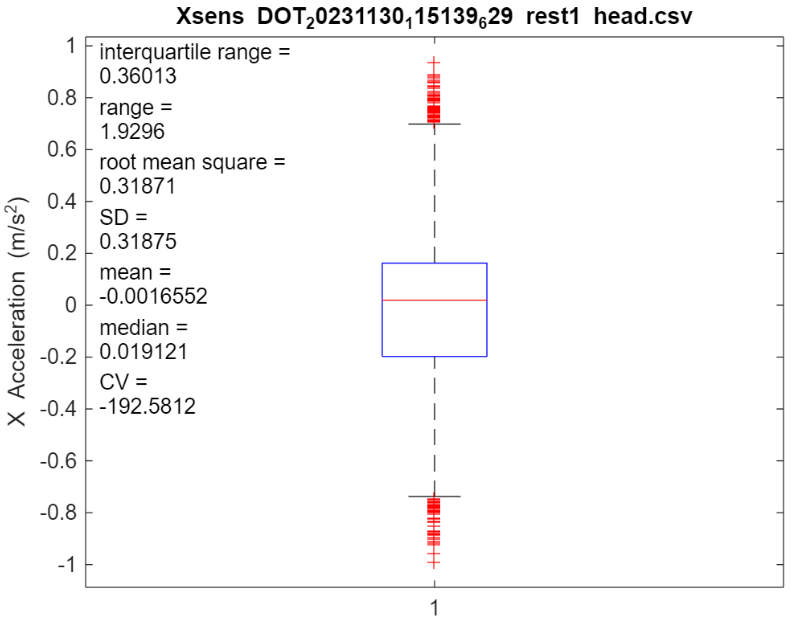
Xsens DOT20231130115139629 rest1 head.csv
X Acceleration (m/s2)
interquartile range =
0.36013
range =
1.9296
root mean square =
0.31871
SD =
0.31875
mean =
-0.0016552
median =
0.019121
CV =
-192.5812
1
0.8
0.6
0.4
0.2
0
-0.2
-0.4
-0.6
-0.8
-1
1

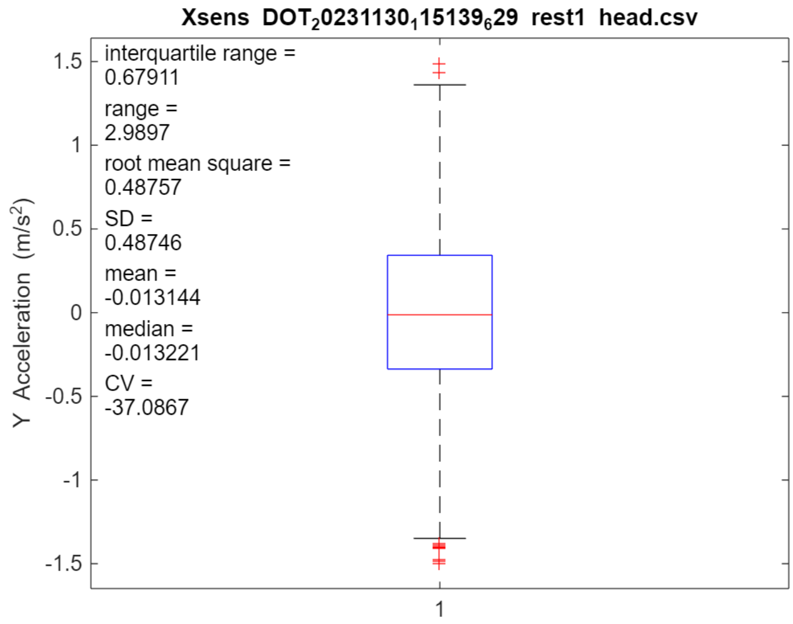

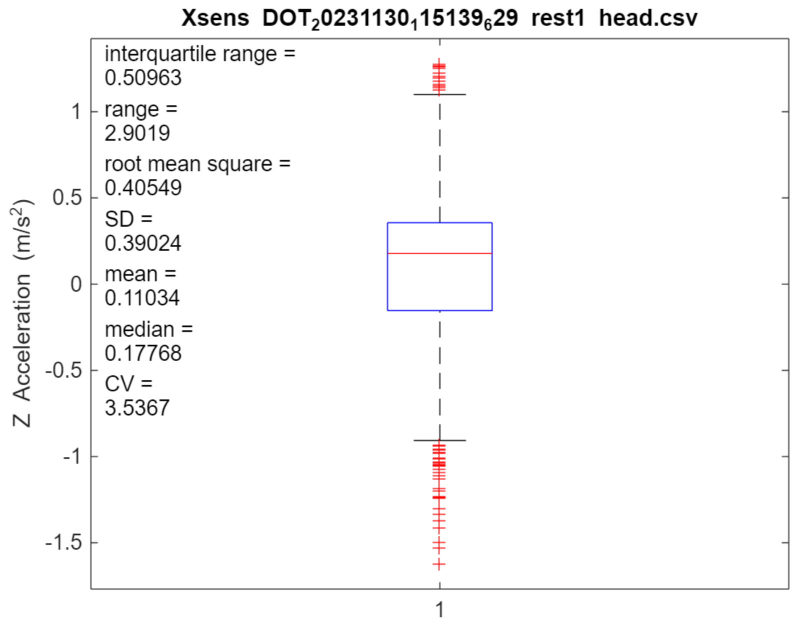

X-Y-Z ANGULAR VELOCITY TIME SERIES STATISTICS FOR IMU #1

Now plot the entire recording and first 4 seconds of x,y,z angular velocity for IMU #1.

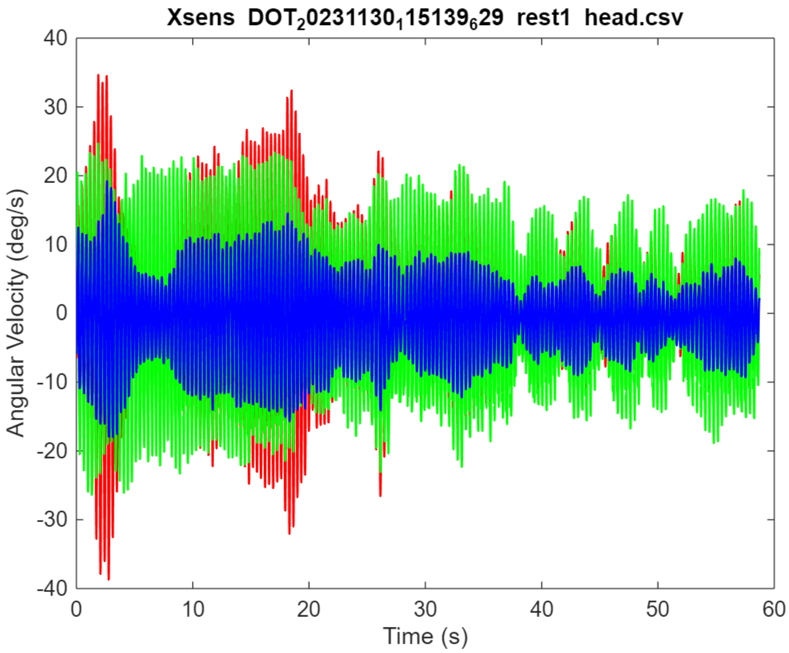
Xsens DOT$_2$0231130$_1$15139$_6$29 rest1 head.csv
Angular Velocity (deg/s)
Time (s)
40
30
20
10
0
-10
-20
-30
-40
0
10
20
30
40
50
60

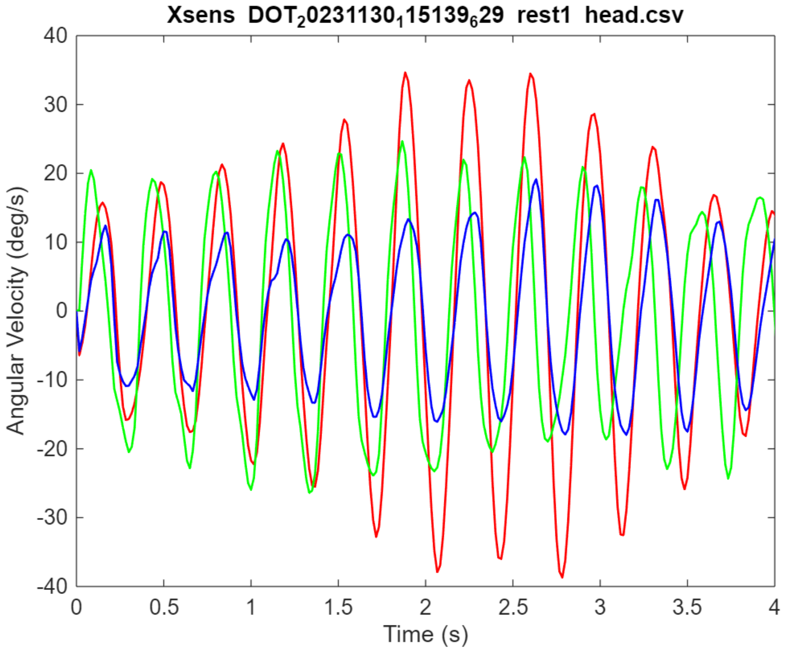
Xsens DOT$_2$0231130$_1$15139$_6$29 rest1 head.csv
Angular Velocity (deg/s)
Time (s)
40
30
20
10
0
-10
-20
-30
-40
0
0.5
1
1.5
2
2.5
3
3.5
4

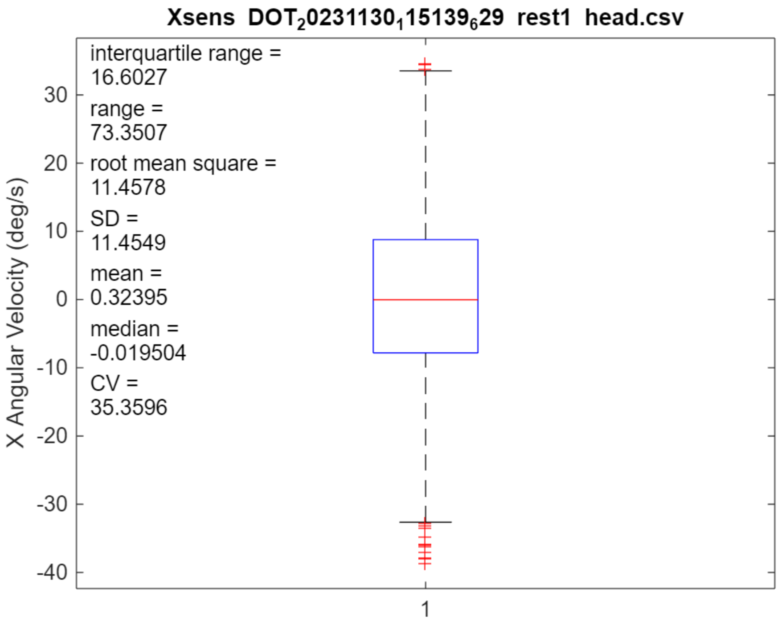
Xsens DOT20231130115139629 rest1 head.csv
interquartile range =
16.6027
range =
73.3507
root mean square =
11.4578
SD =
11.4549
mean =
0.32395
median =
-0.019504
CV =
35.3596
X Angular Velocity (deg/s)
30
20
10
0
-10
-20
-30
-40
1

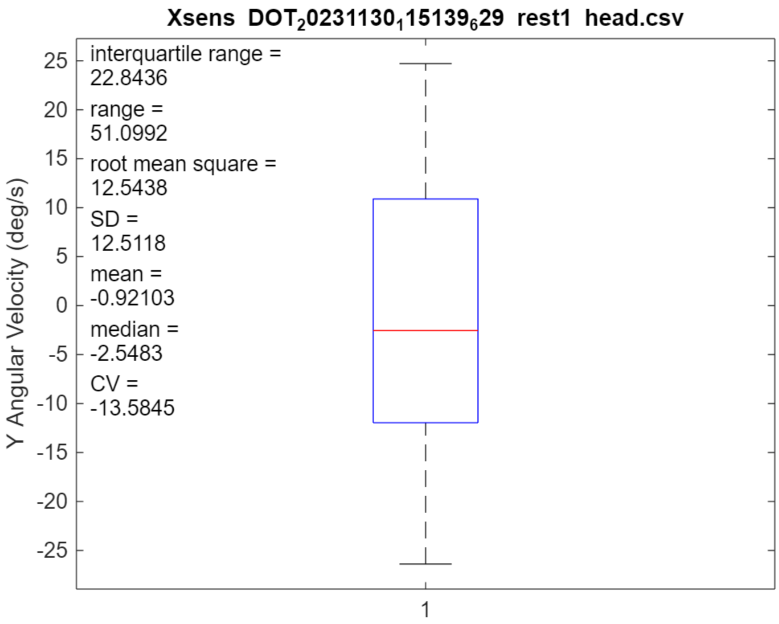
Xsens DOT20231130115139629 rest1 head.csv
interquartile range =
22.8436
range =
51.0992
root mean square =
12.5438
SD =
12.5118
mean =
-0.92103
median =
-2.5483
CV =
-13.5845
Y Angular Velocity (deg/s)
25
20
15
10
5
0
-5
-10
-15
-20
-25
1

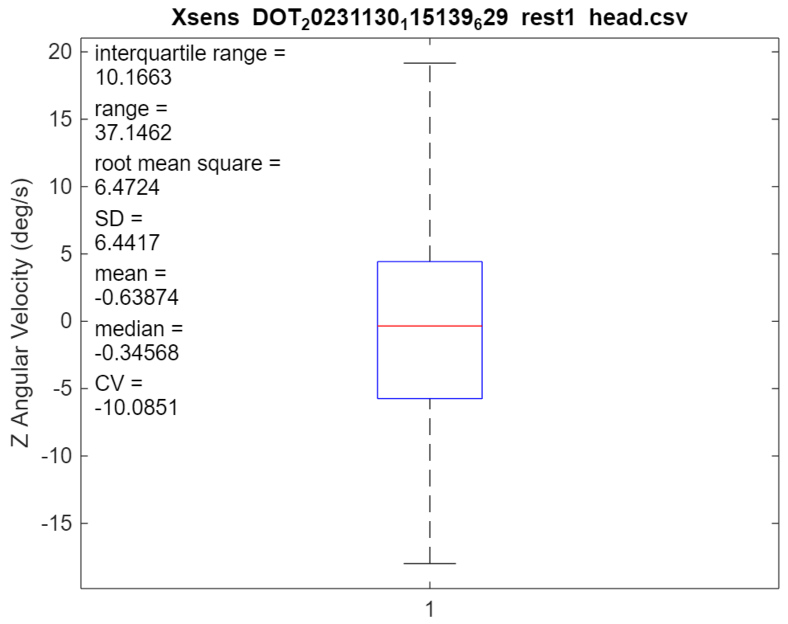

X-Y-Z ACCELERATION TIME SERIES STATISTICS FOR IMU #2

Now plot the entire recording and first 4 seconds of x,y,z acceleration for IMU #2.

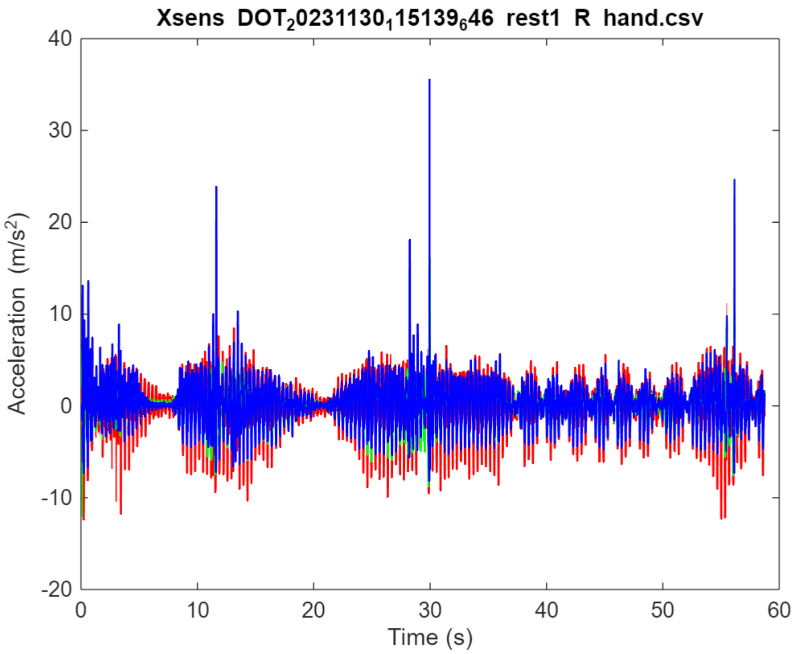

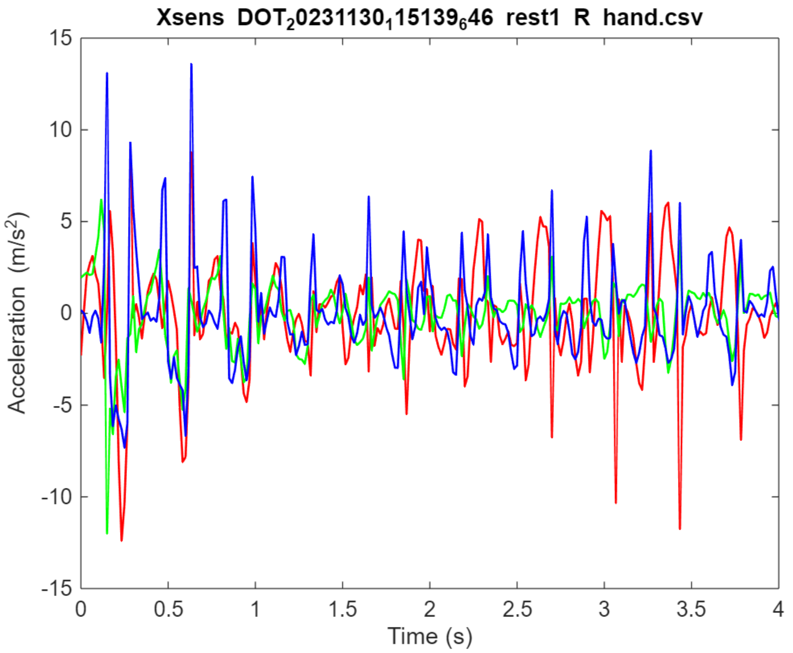
Xsens DOT20231130115139646 rest1 R hand.csv
Acceleration (m/s2)
Time (s)

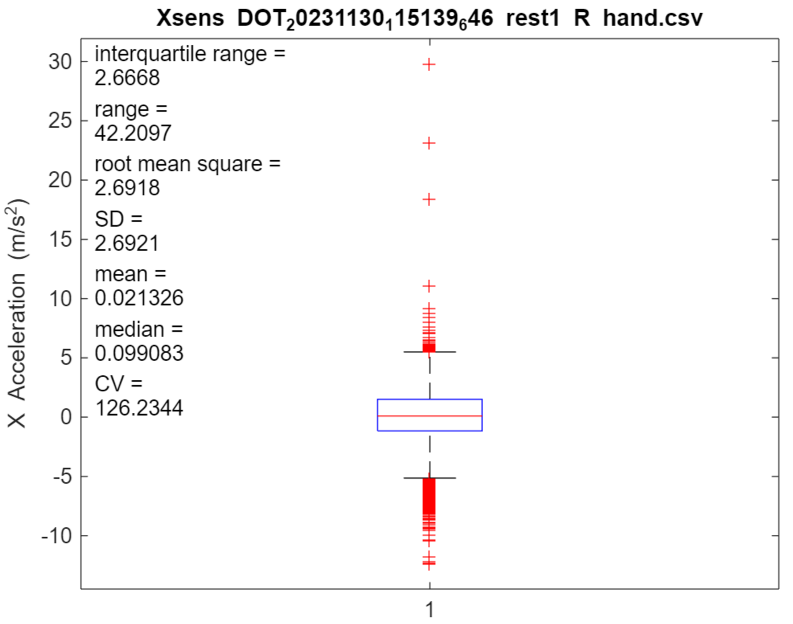
Xsens DOT20231130115139646 rest1 R hand.csv
interquartile range =
2.6668
range =
42.2097
root mean square =
2.6918
SD =
2.6921
mean =
0.021326
median =
0.099083
CV =
126.2344
X Acceleration (m/s2)

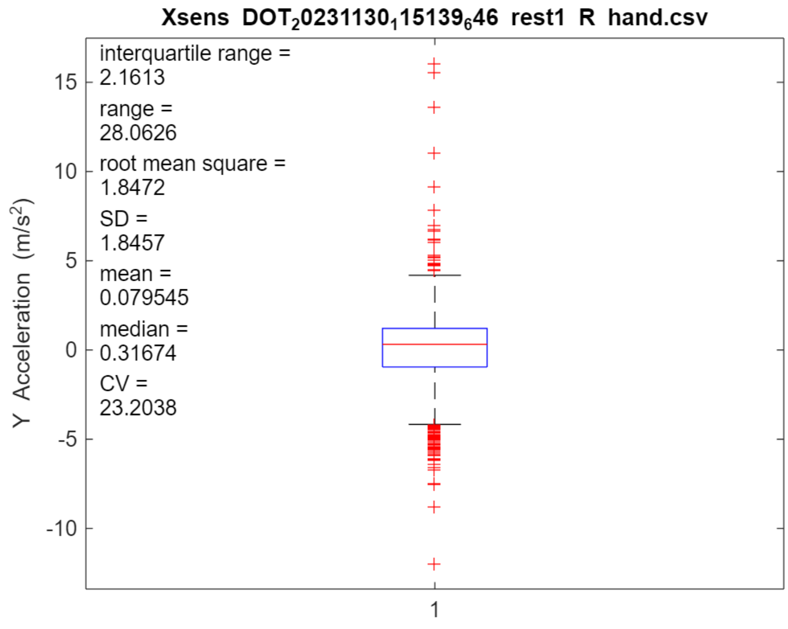

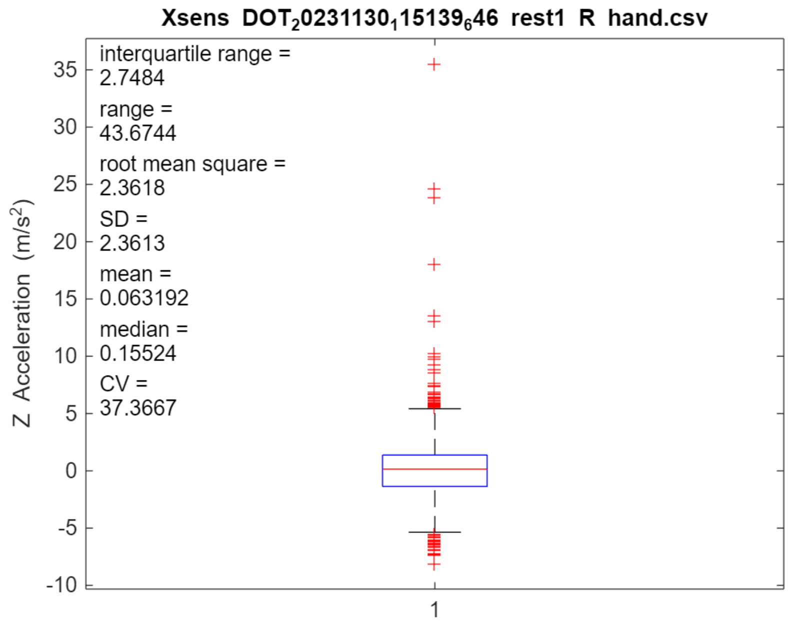

X-Y-Z ANGULAR VELOCITY TIME SERIES STATISTICS FOR IMU #2

Now plot the entire recording and first 4 seconds of x,y,z angular velocity for IMU #2.

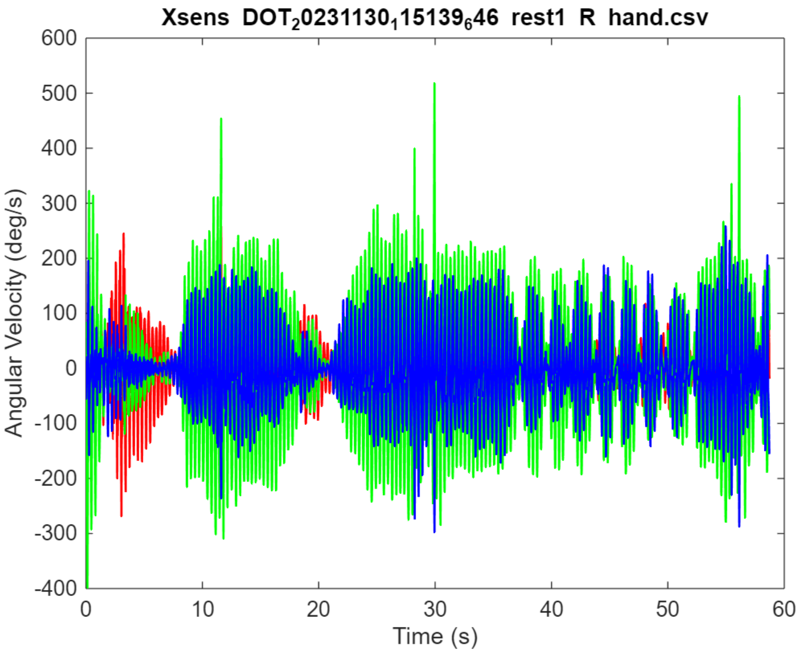
Xsens DOT$_2$0231130$_1$15139$_6$46 rest1 R hand.csv
Angular Velocity (deg/s)
600
500
400
300
200
100
0
-100
-200
-300
-400
0
10
20
30
40
50
60
Time (s)

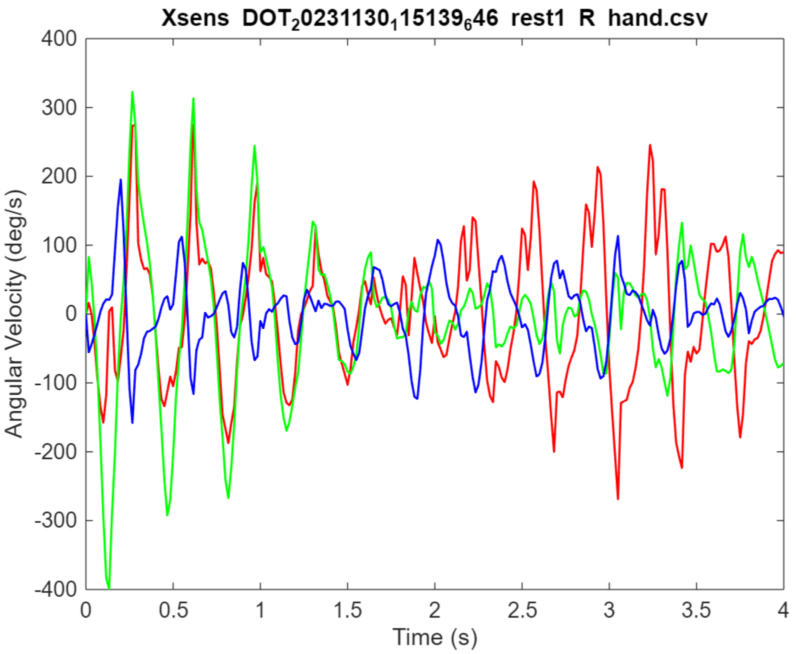
Xsens DOT$_2$0231130$_1$15139$_6$46 rest1 R hand.csv
Angular Velocity (deg/s)
400
300
200
100
0
-100
-200
-300
-400
0
0.5
1
1.5
2
2.5
3
3.5
4
Time (s)

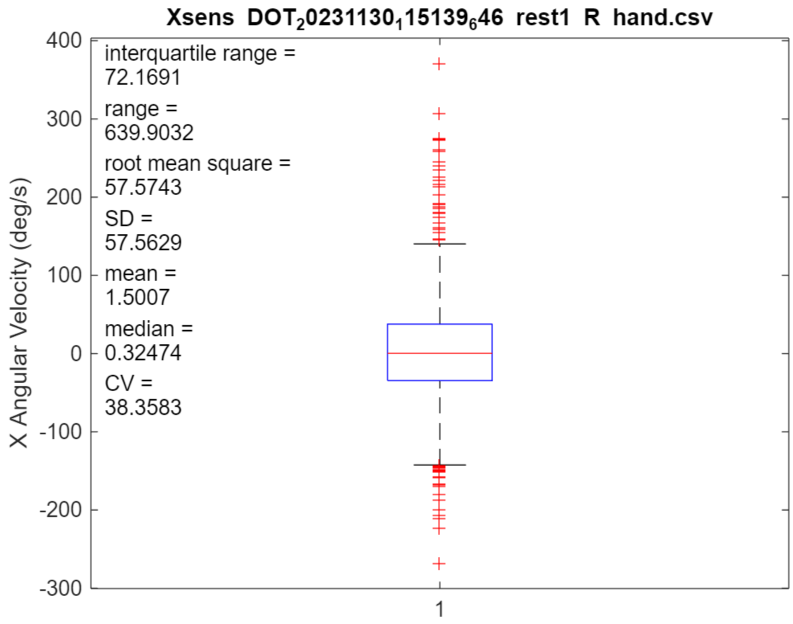
Xsens DOT20231130115139646 rest1 R hand.csv
interquartile range =
72.1691
range =
639.9032
root mean square =
57.5743
SD =
57.5629
mean =
1.5007
median =
0.32474
CV =
38.3583
X Angular Velocity (deg/s)
400
300
200
100
0
-100
-200
-300
1

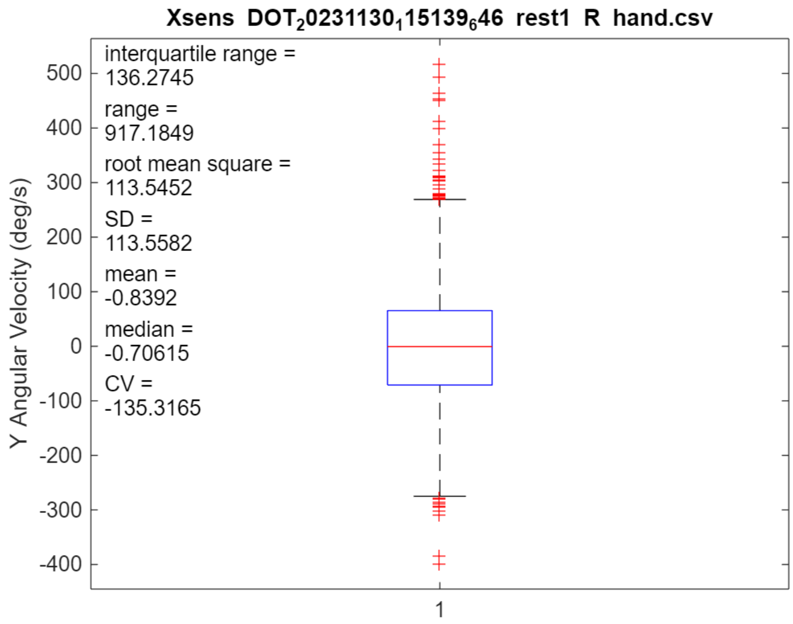
Xsens DOT20231130115139646 rest1 R hand.csv
interquartile range =
136.2745
range =
917.1849
root mean square =
113.5452
SD =
113.5582
mean =
-0.8392
median =
-0.70615
CV =
-135.3165
Y Angular Velocity (deg/s)
500
400
300
200
100
0
-100
-200
-300
-400
1

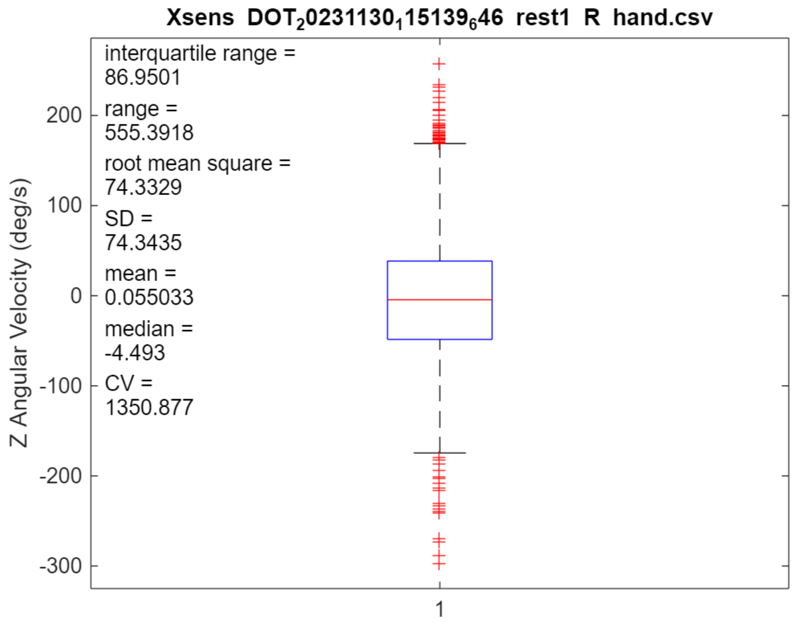

Frequency stability analyses of the two IMUs.

IMU #1 - Acceleration (m/s^2)

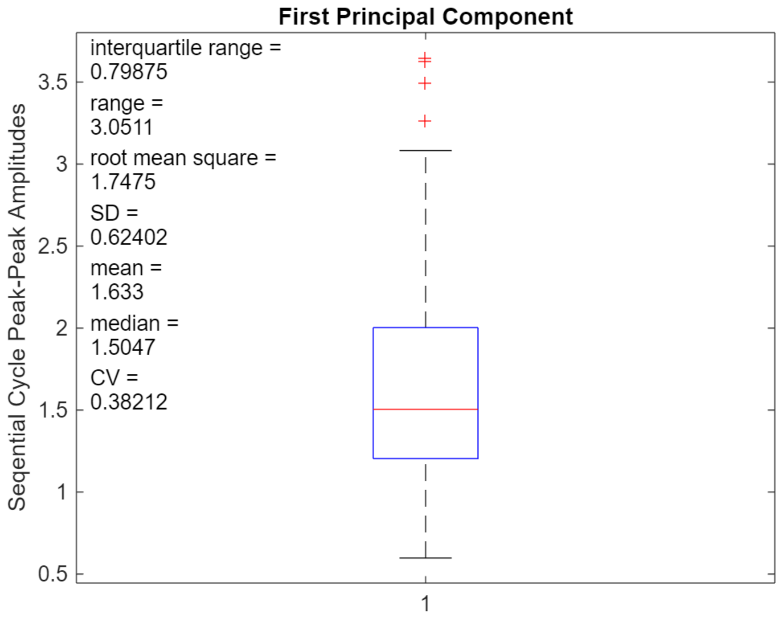

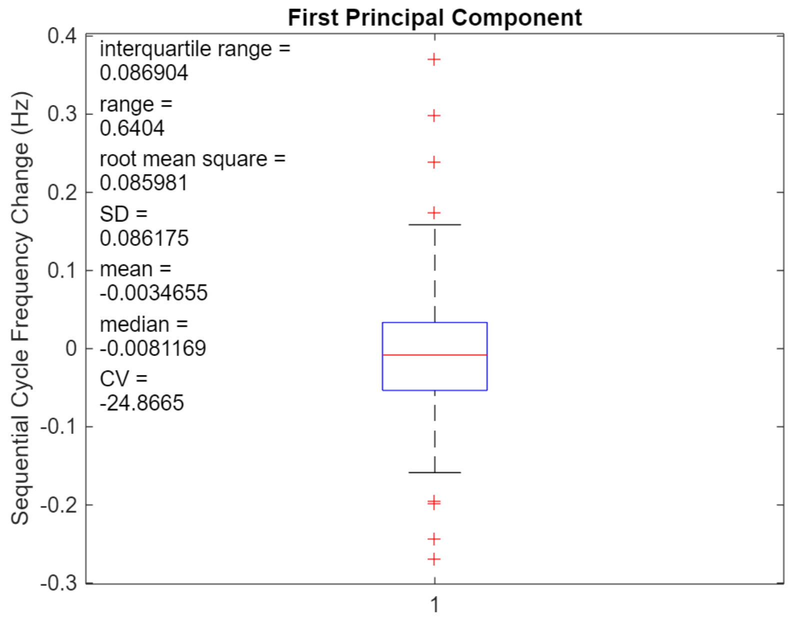
First Principal Component
interquartile range =
0.086904
range =
0.6404
root mean square =
0.085981
SD =
0.086175
mean =
-0.0034655
median =
-0.0081169
CV =
-24.8665
Sequential Cycle Frequency Change (Hz)
0.4
0.3
0.2
0.1
0
-0.1
-0.2
-0.3
1

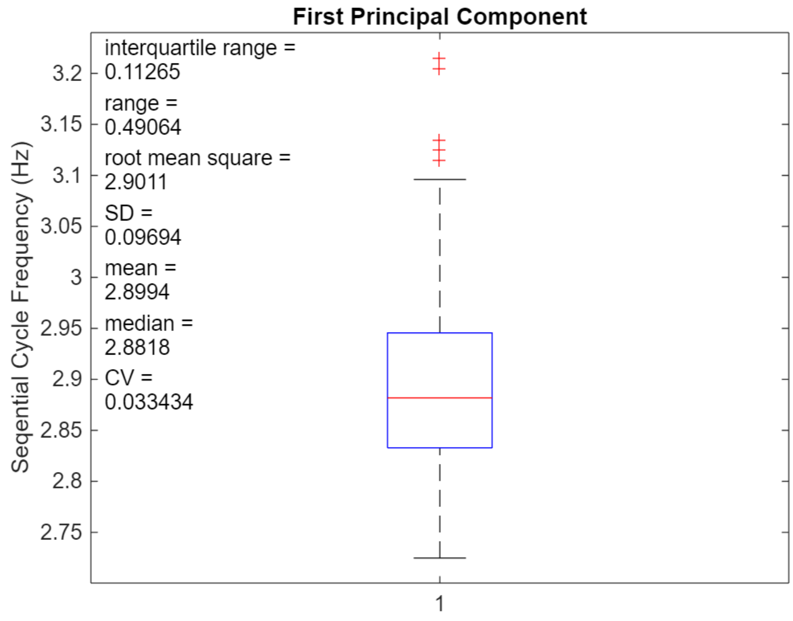
First Principal Component
interquartile range =
0.11265
range =
0.49064
root mean square =
2.9011
SD =
0.09694
mean =
2.8994
median =
2.8818
CV =
0.033434
Seqential Cycle Frequency (Hz)
3.2
3.15
3.1
3.05
3
2.95
2.9
2.85
2.8
2.75
1

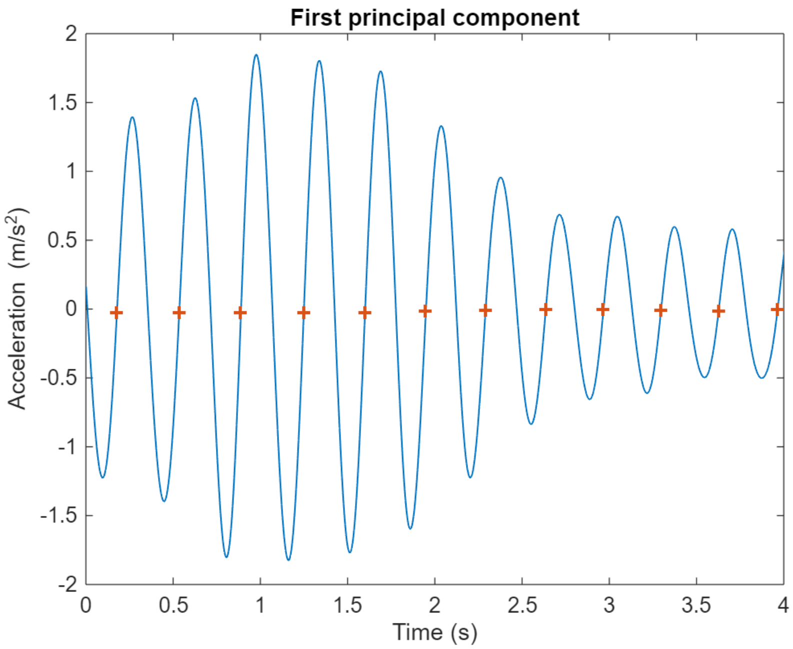

IMU #1 - Angular velocity (deg/s)

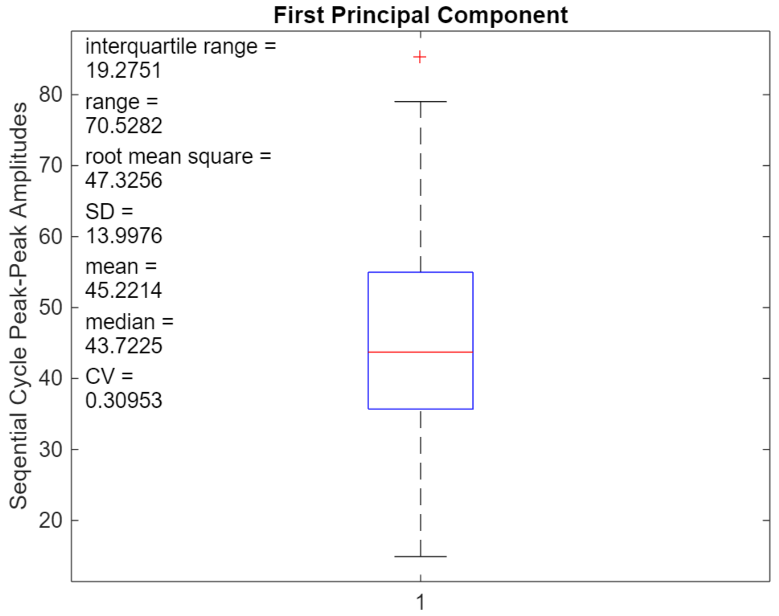

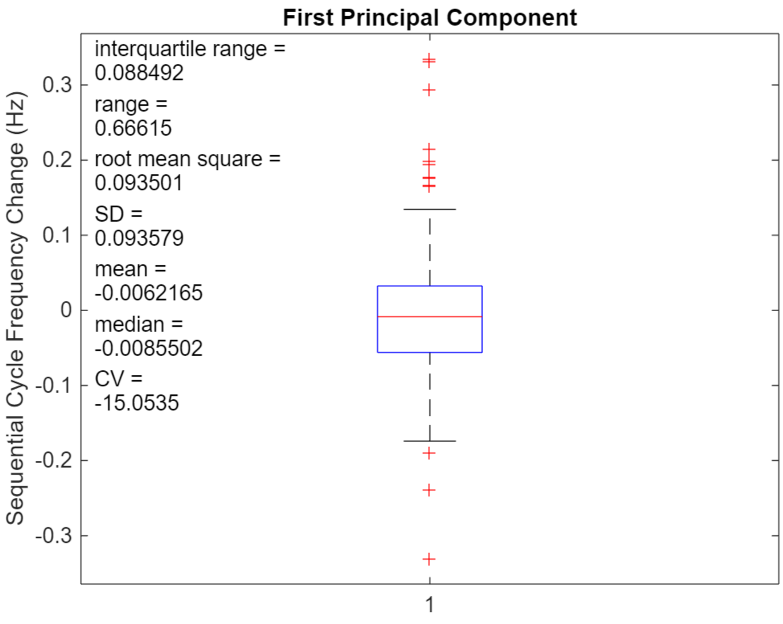
First Principal Component
interquartile range =
0.088492
range =
0.66615
root mean square =
0.093501
SD =
0.093579
mean =
-0.0062165
median =
-0.0085502
CV =
-15.0535
Sequential Cycle Frequency Change (Hz)
0.3
0.2
0.1
0
-0.1
-0.2
-0.3
1

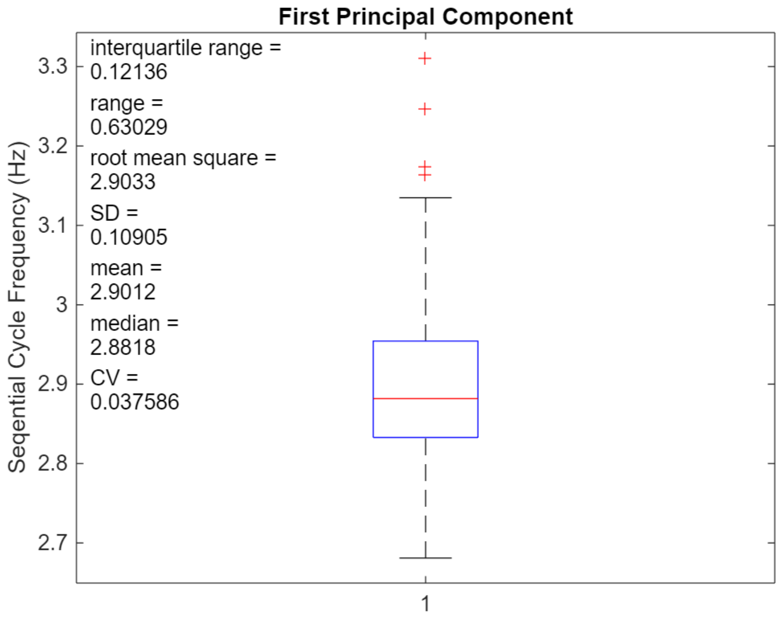
First Principal Component
interquartile range =
0.12136
range =
0.63029
root mean square =
2.9033
SD =
0.10905
mean =
2.9012
median =
2.8818
CV =
0.037586
Seqential Cycle Frequency (Hz)
3.3
3.2
3.1
3
2.9
2.8
2.7
1

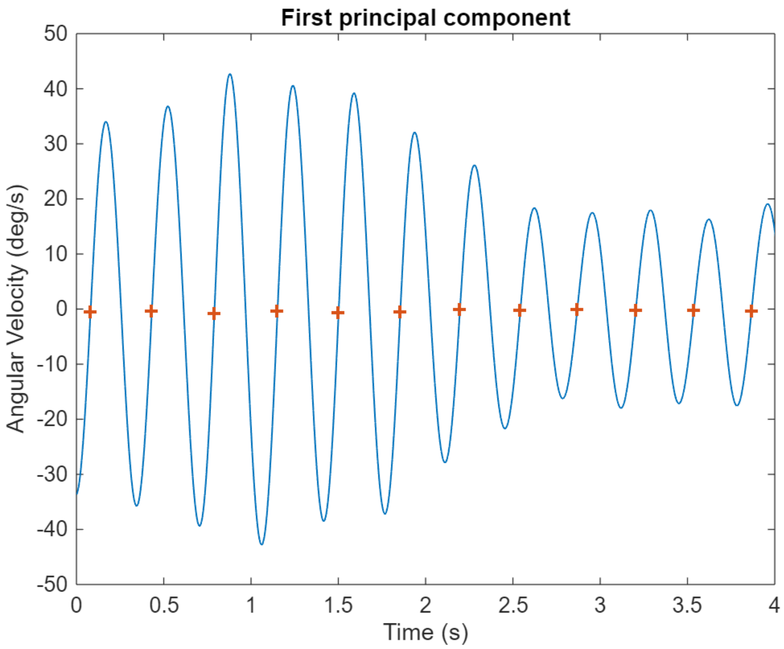

IMU #2 - Acceleration (m/s^2)

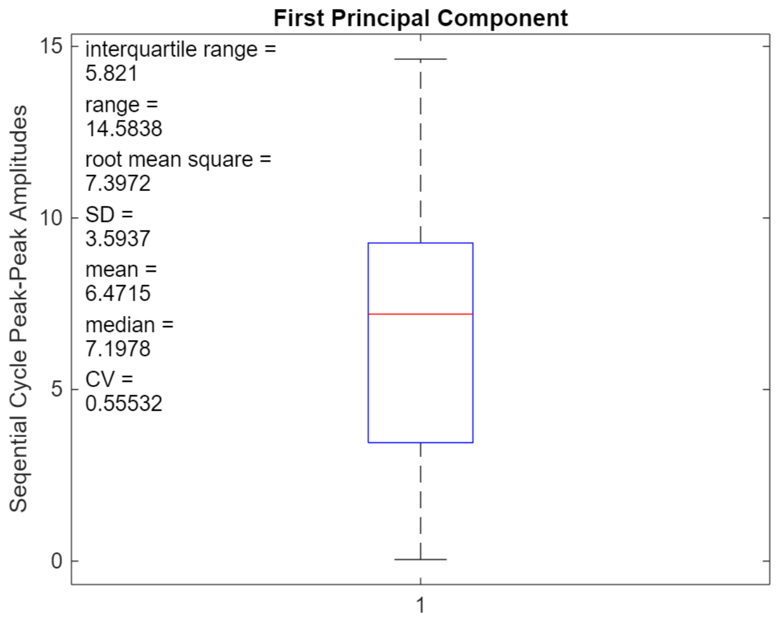

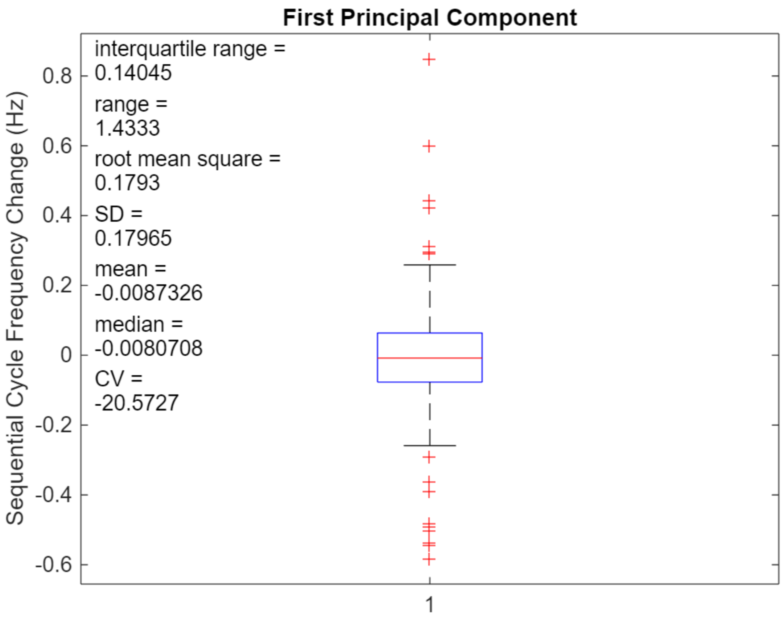
First Principal Component
interquartile range =
0.14045
range =
1.4333
root mean square =
0.1793
SD =
0.17965
mean =
-0.0087326
median =
-0.0080708
CV =
-20.5727
Sequential Cycle Frequency Change (Hz)
0.8
0.6
0.4
0.2
0
-0.2
-0.4
-0.6
1

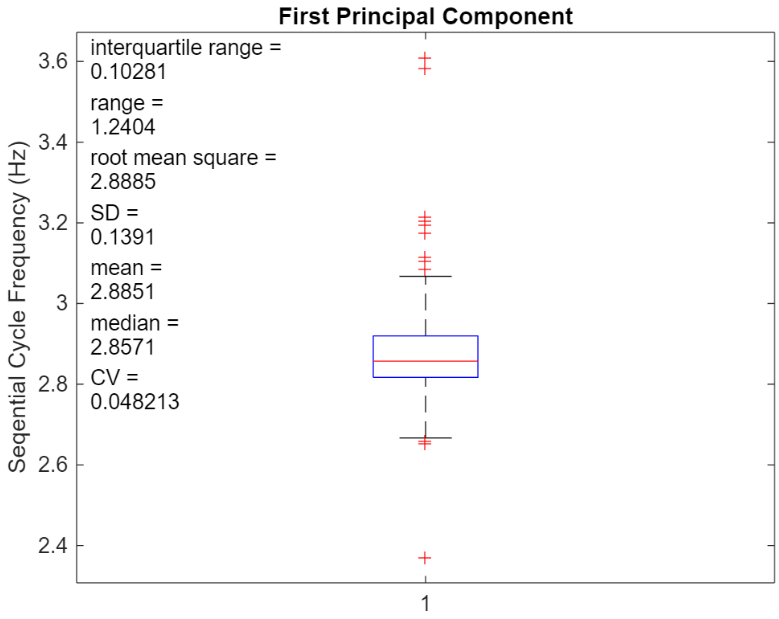
First Principal Component
interquartile range =
0.10281
range =
1.2404
root mean square =
2.8885
SD =
0.1391
mean =
2.8851
median =
2.8571
CV =
0.048213
Seqential Cycle Frequency (Hz)
3.6
3.4
3.2
3
2.8
2.6
2.4
1

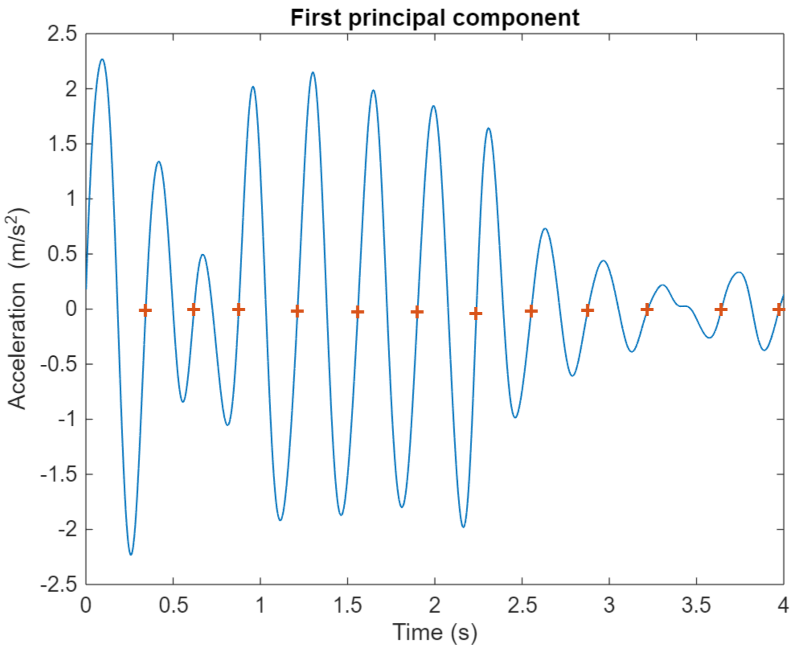

IMU #2 - Angular velocity (deg/s)

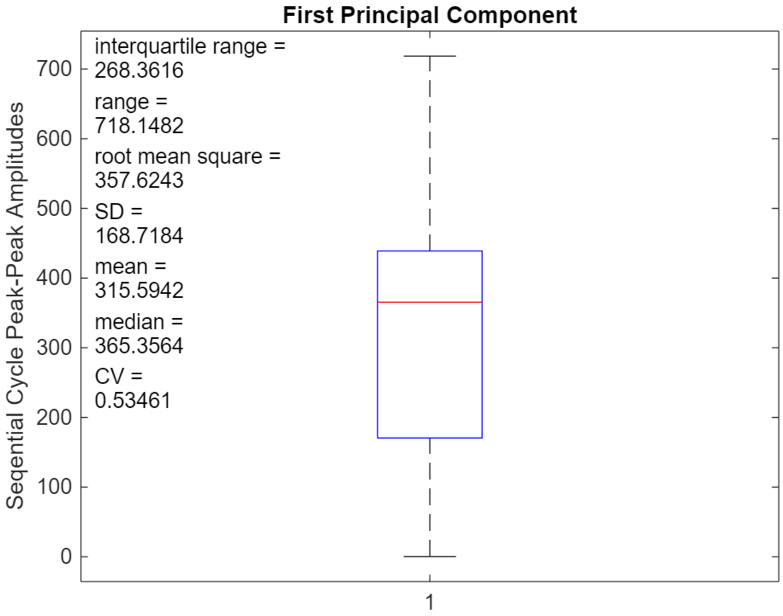

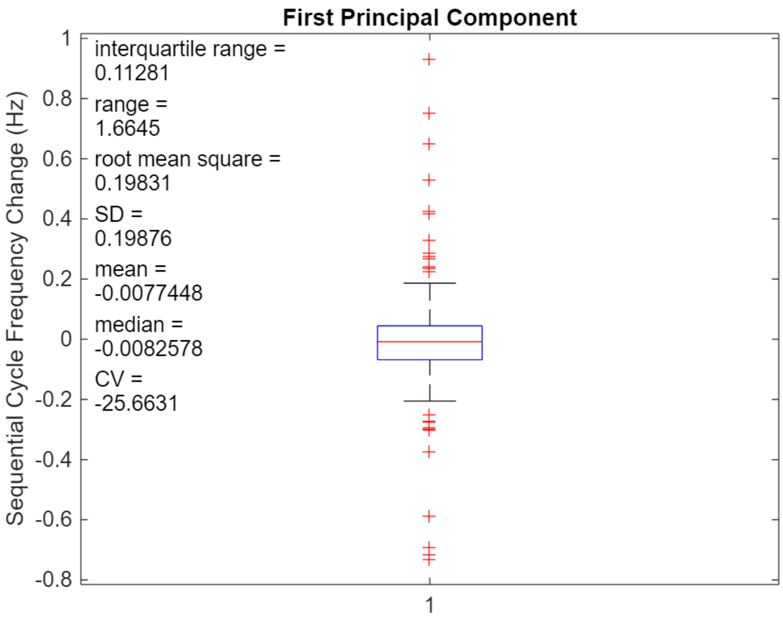
First Principal Component
interquartile range =
0.11281
range =
1.6645
root mean square =
0.19831
SD =
0.19876
mean =
-0.0077448
median =
-0.0082578
CV =
-25.6631
Sequential Cycle Frequency Change (Hz)
1
0.8
0.6
0.4
0.2
0
-0.2
-0.4
-0.6
-0.8
1

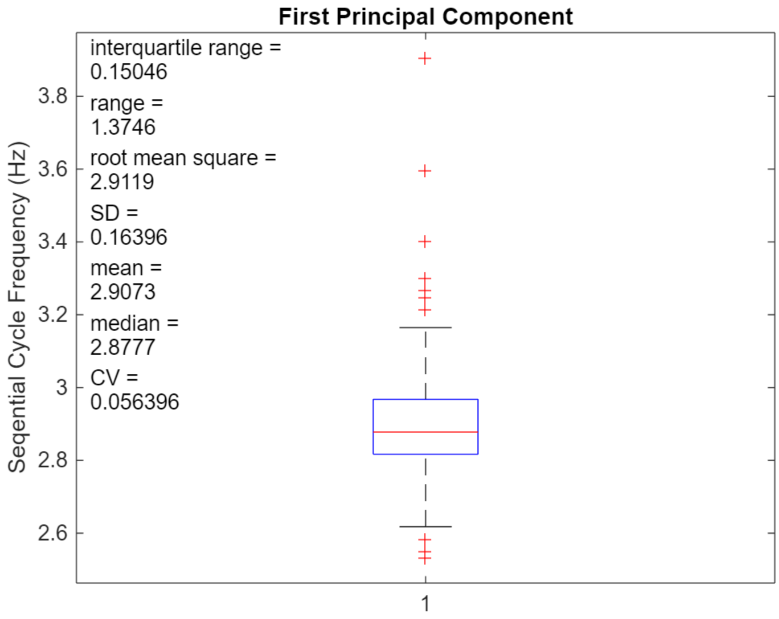
First Principal Component
interquartile range =
0.15046
range =
1.3746
root mean square =
2.9119
SD =
0.16396
mean =
2.9073
median =
2.8777
CV =
0.056396
Seqential Cycle Frequency (Hz)
3.8
3.6
3.4
3.2
3
2.8
2.6
1

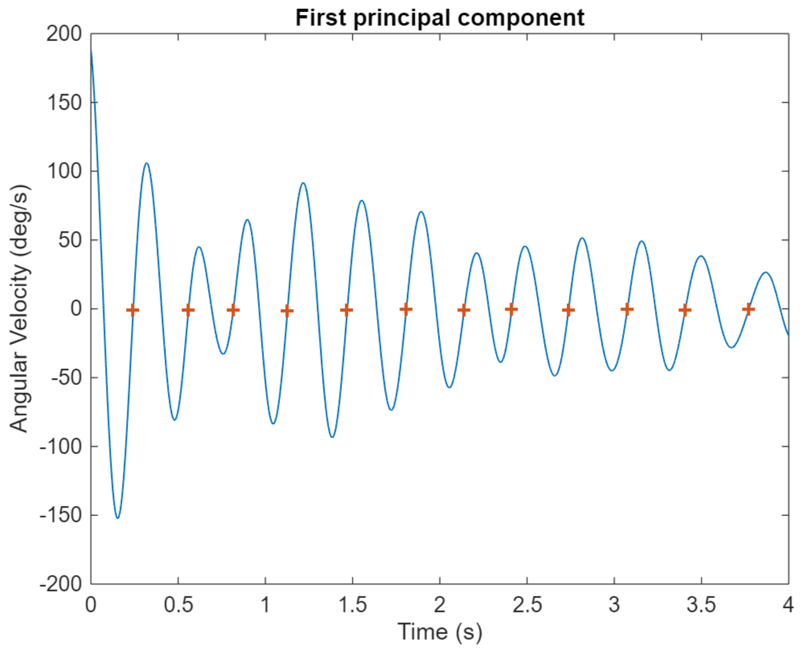

Now, compute the power spectra of acceleration and angular velocity for IMU #1.

```
Effective number of segments used in the Welch power spectrum: 8
Effective degrees of freedom of Welch spectral estimates: 16
Harmonic distortion: 0.25
Frequency of the fundamental peak and higher harmonics:
    2.9004    5.7129    8.5547

Peak power of the fundamental peak and higher harmonics:
    0.2003    0.0069    0.0053

Mean peak-to-peak displacement (cm): 0.40
```

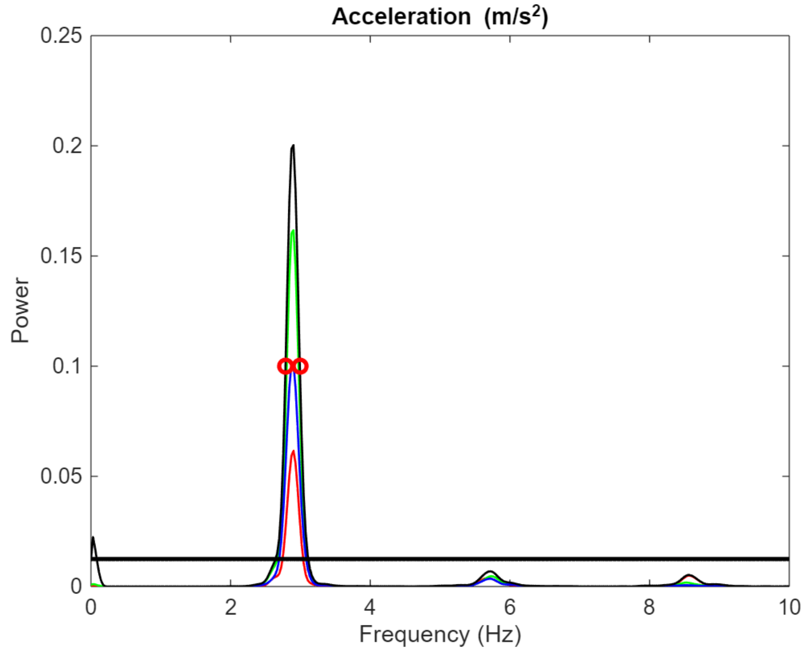


```
Half-power bandwidth of the fundamental peak: 0.21
Axis with greatest acceleration power: 2
Harmonic distortion: 0.12
Frequency of the fundamental peak and higher harmonics:
```

```
    2.9004    5.7129

Peak power of the fundamental peak and higher harmonics:
  152.7103    2.1589

Mean peak-to-peak rotation (deg): 2.01
```

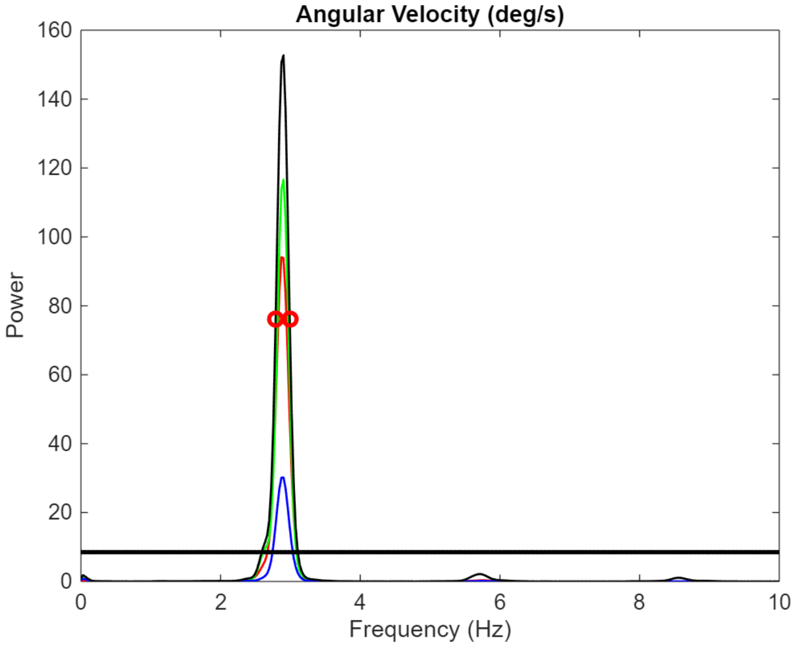


```
Half-power bandwidth of the fundamental peak: 0.21
Axis with greatest angular velocity power: 2
```

Now, compute the power spectra of acceleration and angular velocity for IMU #2.

```
Effective number of segments used in the Welch power spectrum: 8
Effective degrees of freedom of Welch spectral estimates: 16
Harmonic distortion: 0.76
Frequency of the fundamental peak and higher harmonics:
    2.8418    5.7422    8.5547   11.4258   14.1504

Peak power of the fundamental peak and higher harmonics:
    2.4255    0.6641    0.6224    0.0742    0.0344

Mean peak-to-peak displacement (cm): 1.45
```

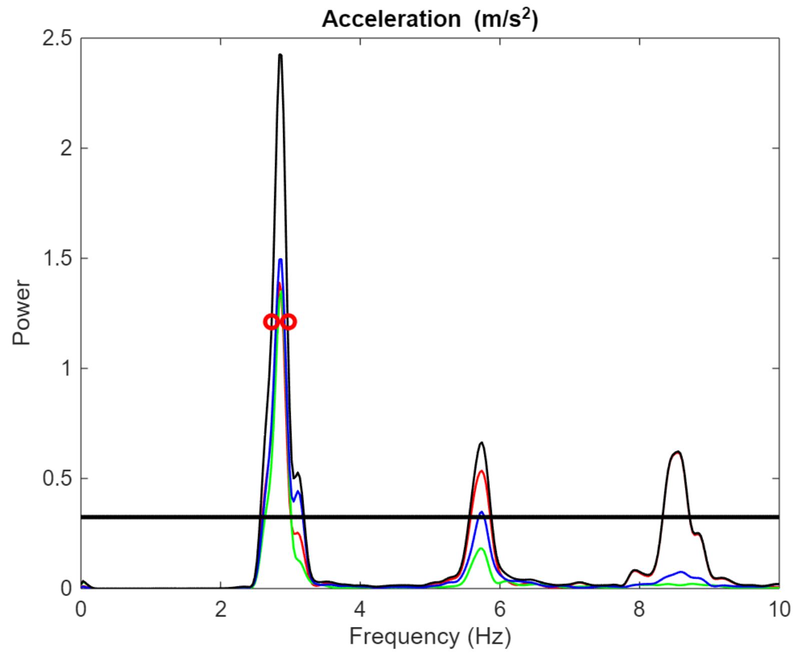


```
Half-power bandwidth of the fundamental peak: 0.23
Axis with greatest acceleration power: 2
Harmonic distortion: 0.29
Frequency of the fundamental peak and higher harmonics:
    2.8711    5.7129

Peak power of the fundamental peak and higher harmonics:
   1.0e+03 *

    7.4006    0.6199

Mean peak-to-peak rotation (deg): 14.16
```

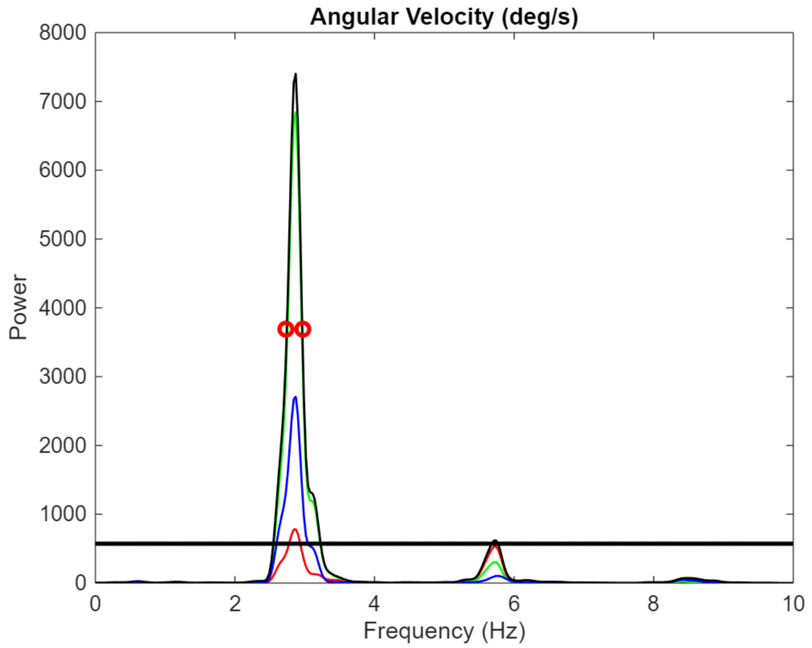


```
Half-power bandwidth of the fundamental peak: 0.23
Axis with greatest angular velocity power: 2
```

Now compute the coherence spectra between the two transducers.

Acceleration coherence

```
Maximum x-x coherence in the 2-4 Hz band : 0.68
Maximum y-y coherence in the 2-4 Hz band : 0.50
Maximum z-z average coherence in the 2-4 Hz band : 0.38
95 percent CL for coherence threshold: 0.17
99 percent CL for coherence threshold: 0.25
Effective number of segments: 17
```

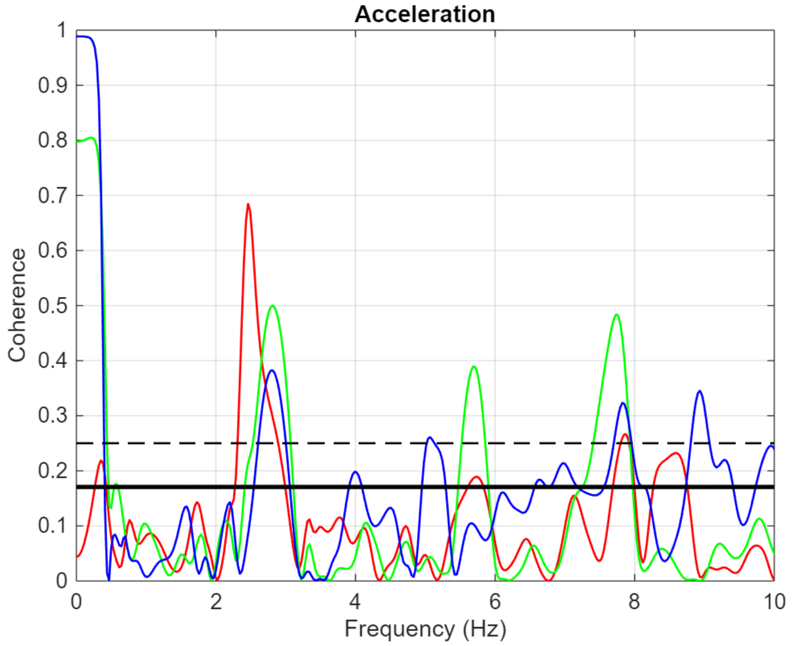

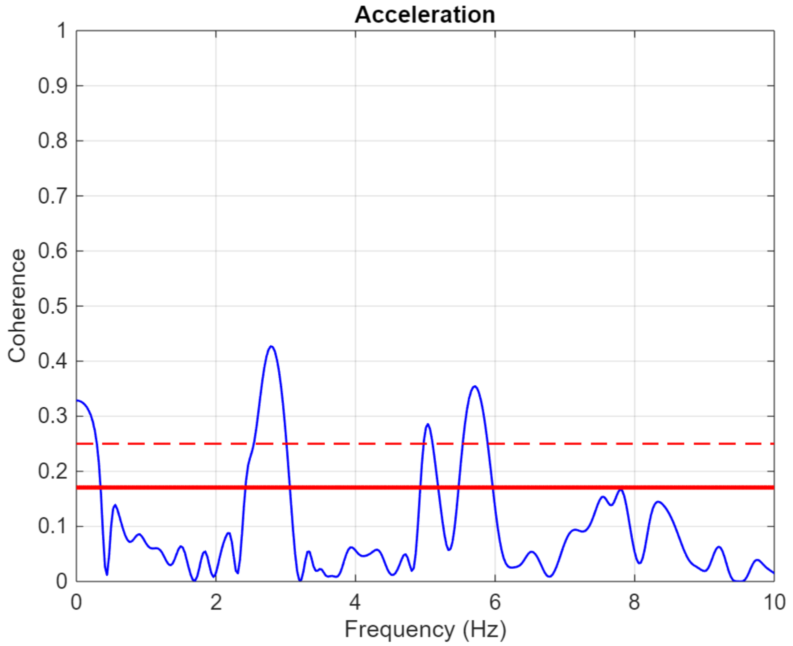


```
Maximum coherence in the 2-4 Hz band : 0.43
IMU #1 and #2 axes with greatest acceleration power: 2 and 3
```

Angular velocity coherence

```
Maximum x-x coherence in the 2-4 Hz band : 0.48
Maximum y-y coherence in the 2-4 Hz band : 0.57
Maximum z-z average coherence in the 2-4 Hz band : 0.36
95 percent CL for coherence: 0.17
99 percent CL for coherence threshold: 0.25
Effective number of segments: 17
```

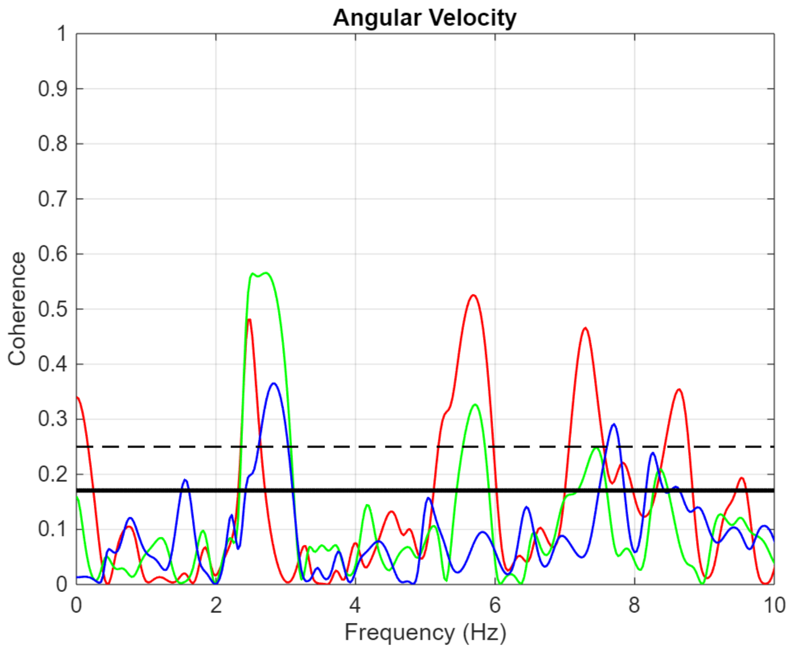

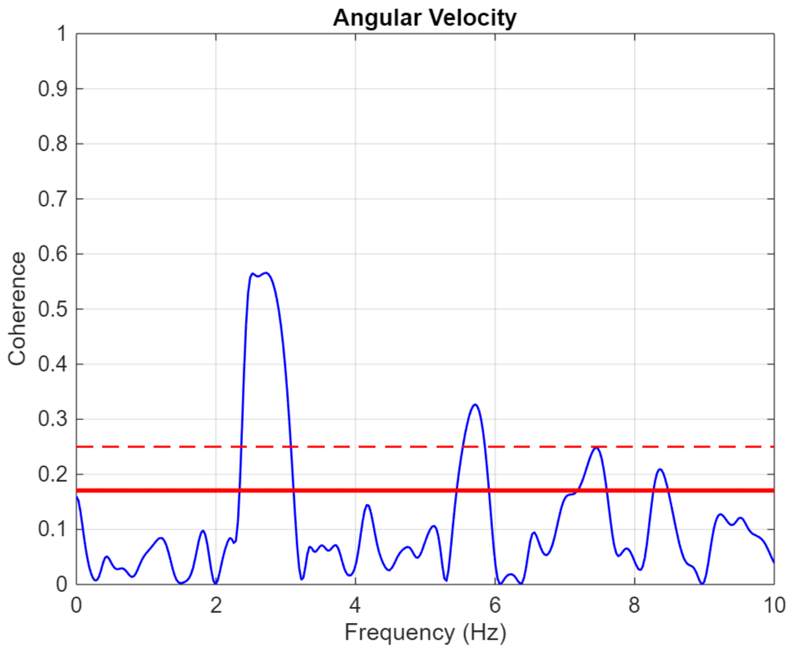


```
Maximum coherence in the 2-4 Hz band : 0.57
IMU #1 and #2 axes with greatest angular velocity power: 2 and 2
```

Now compute the time-frequency autospectra of x,y,z accelerometer #1

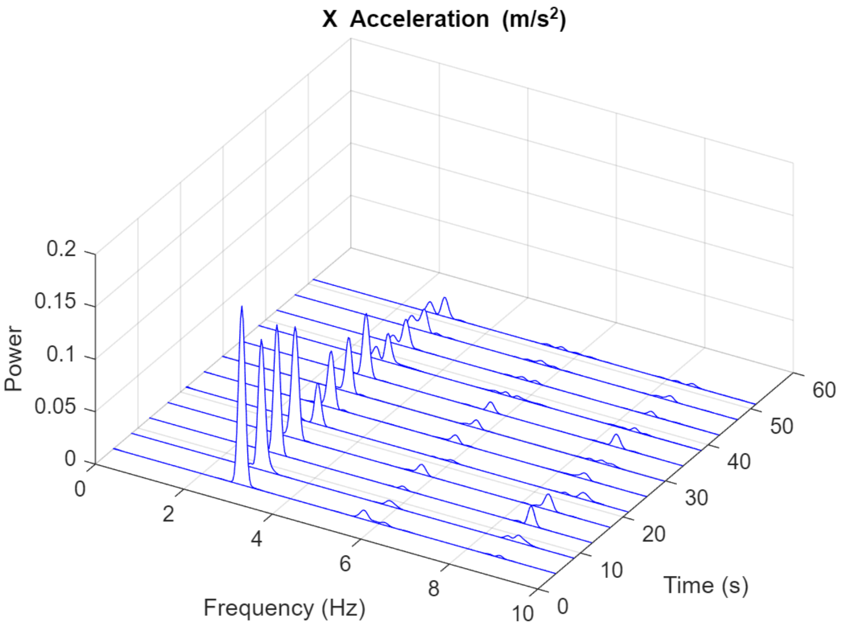
X Acceleration (m/s2)
Power
0.2
0.15
0.1
0.05
0
Frequency (Hz)
0
2
4
6
8
10
Time (s)
0
10
20
30
40
50
60

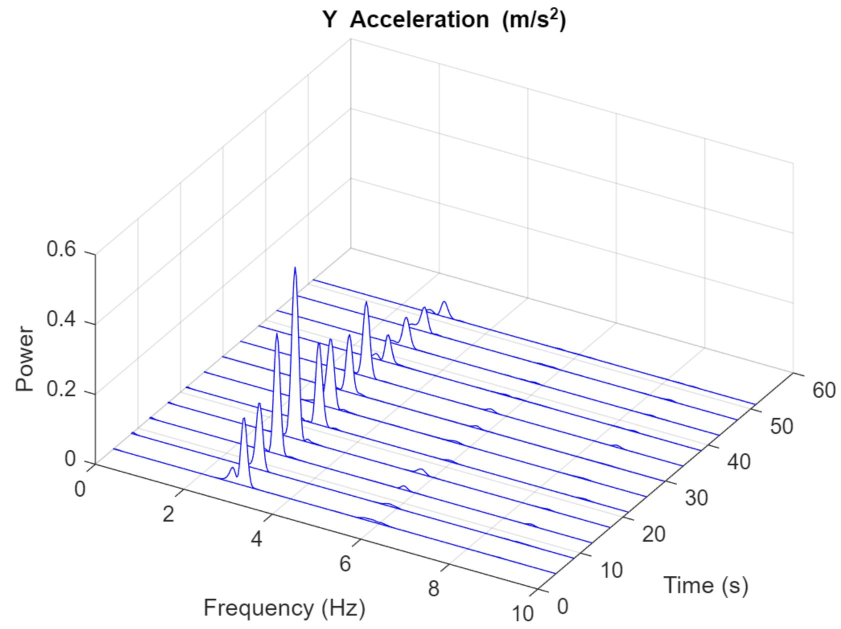
Y Acceleration (m/s2)
Power
0.6
0.4
0.2
0
Frequency (Hz)
0
2
4
6
8
10
Time (s)
0
10
20
30
40
50
60

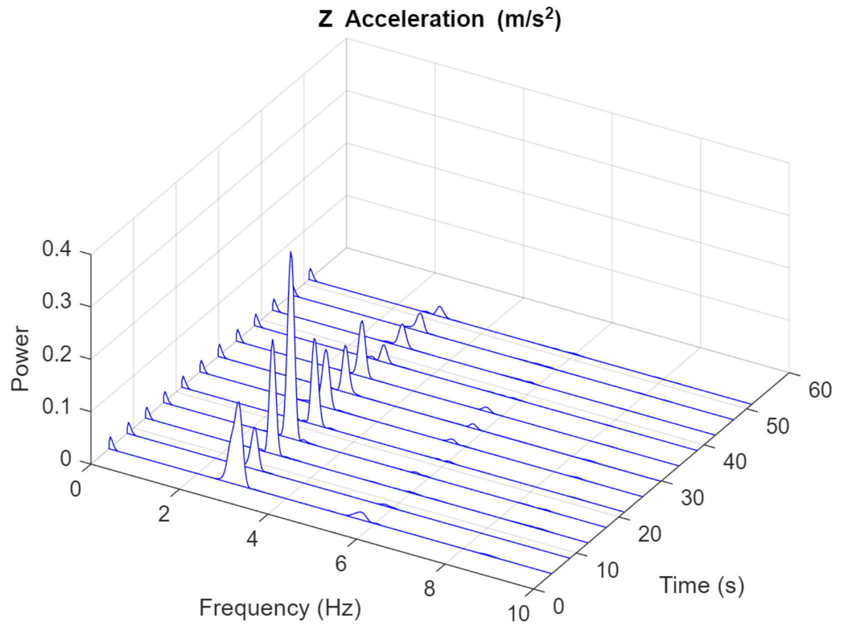

Now compute the time-frequency autospectra of x,y,z accelerometer #2

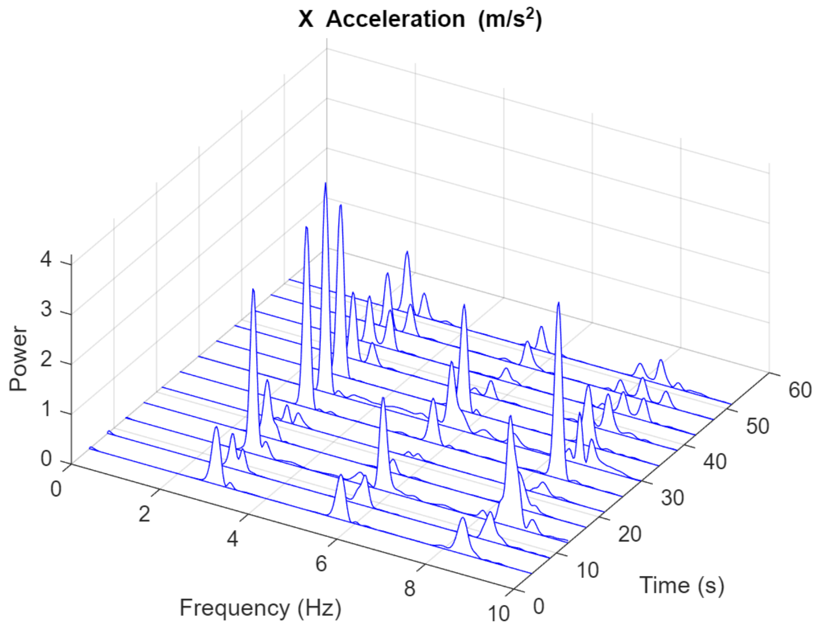

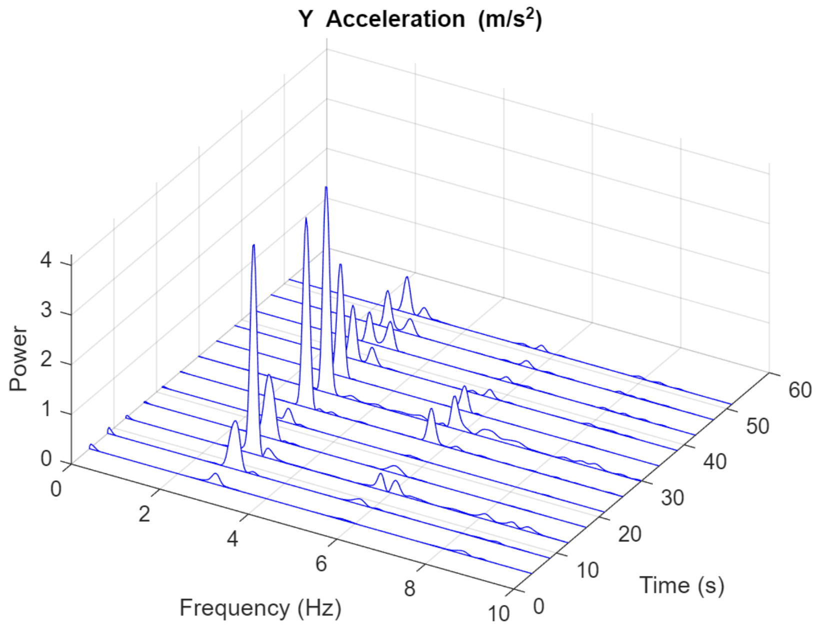

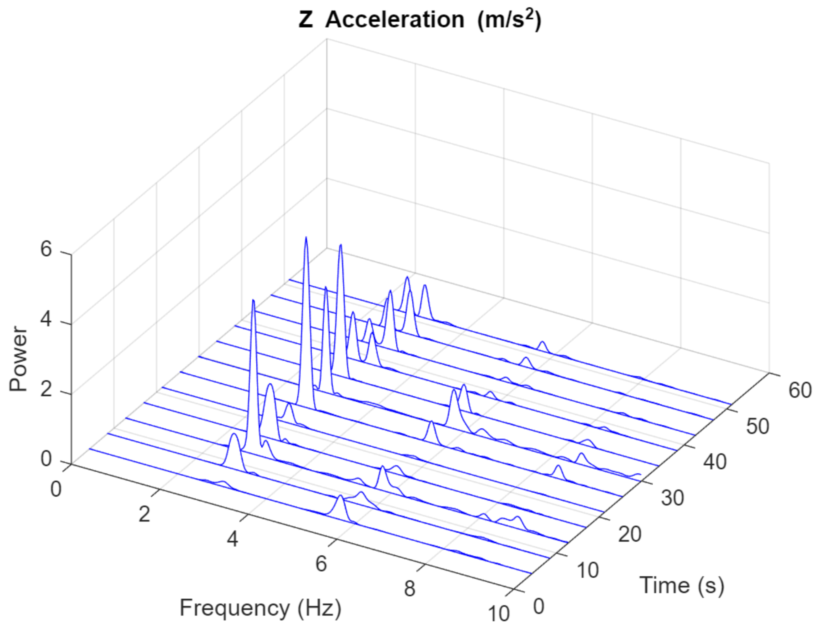

Now compute the time-frequency autospectra of x,y,z gyroscope IMU #1

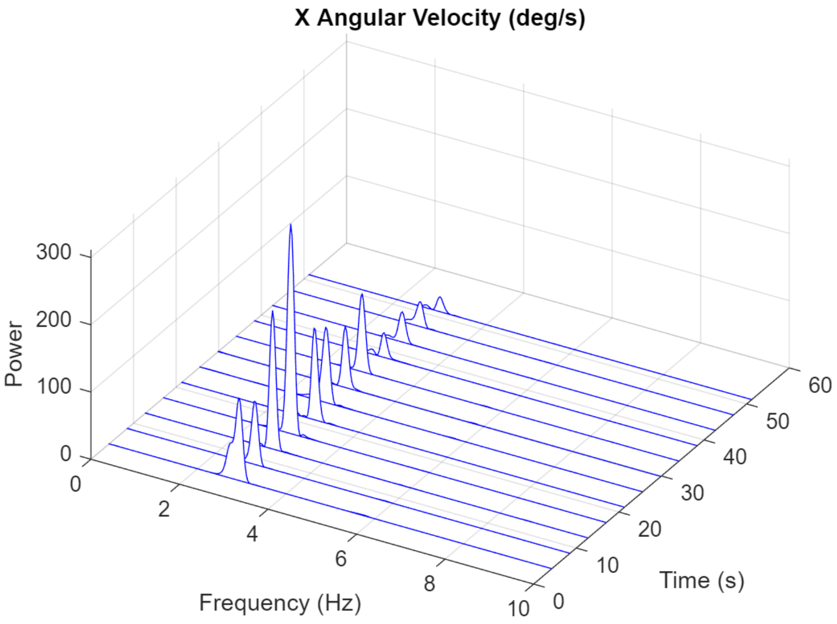
X Angular Velocity (deg/s)
Power
300
200
100
0
0
2
4
6
8
10
Frequency (Hz)
0
10
20
30
40
50
60
Time (s)

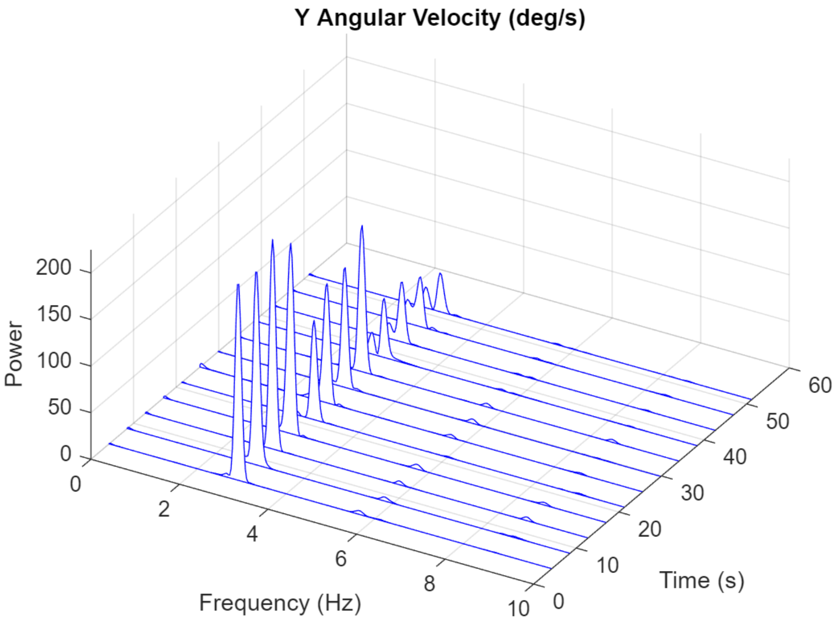
Y Angular Velocity (deg/s)
Power
200
150
100
50
0
0
2
4
6
8
10
Frequency (Hz)
0
10
20
30
40
50
60
Time (s)

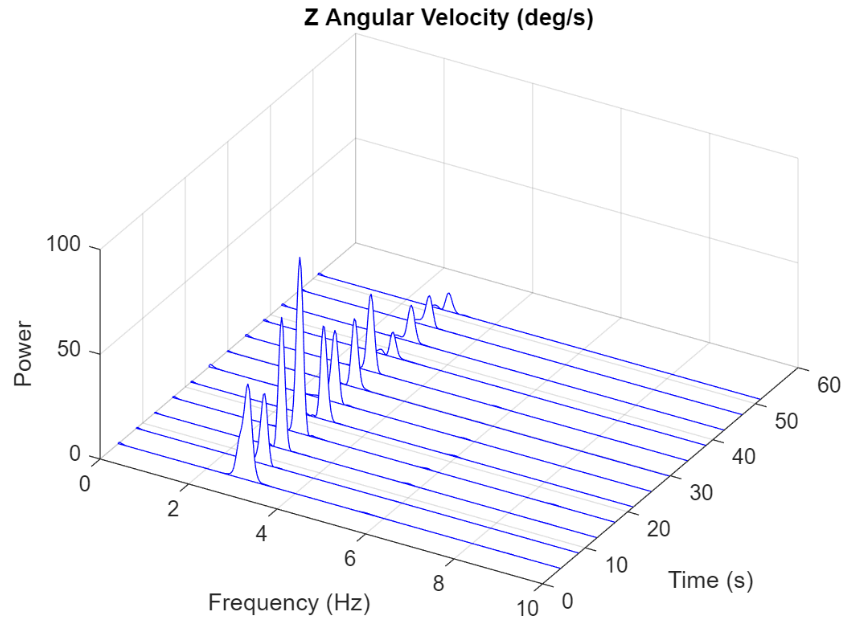

Now compute the time-frequency autospectra of x,y,z gyroscope IMU #2

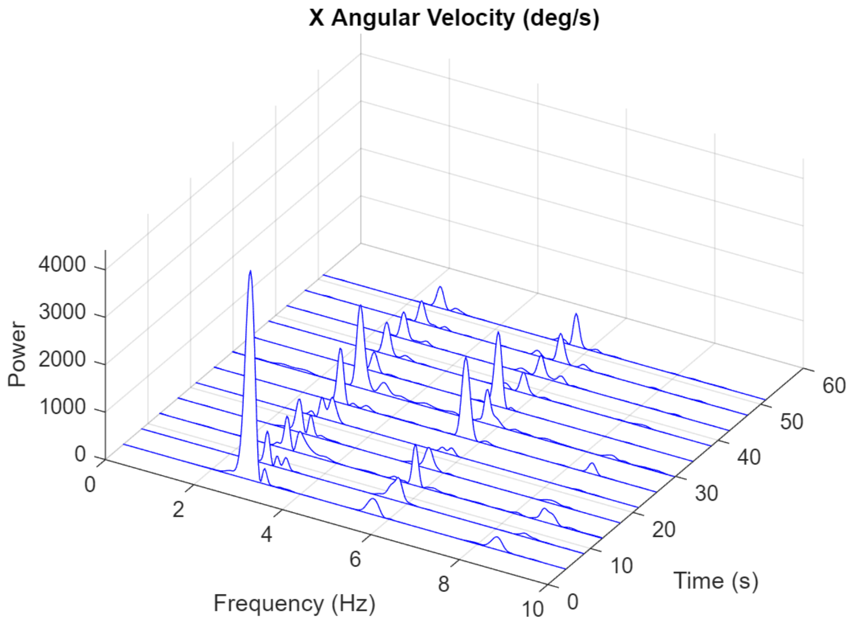

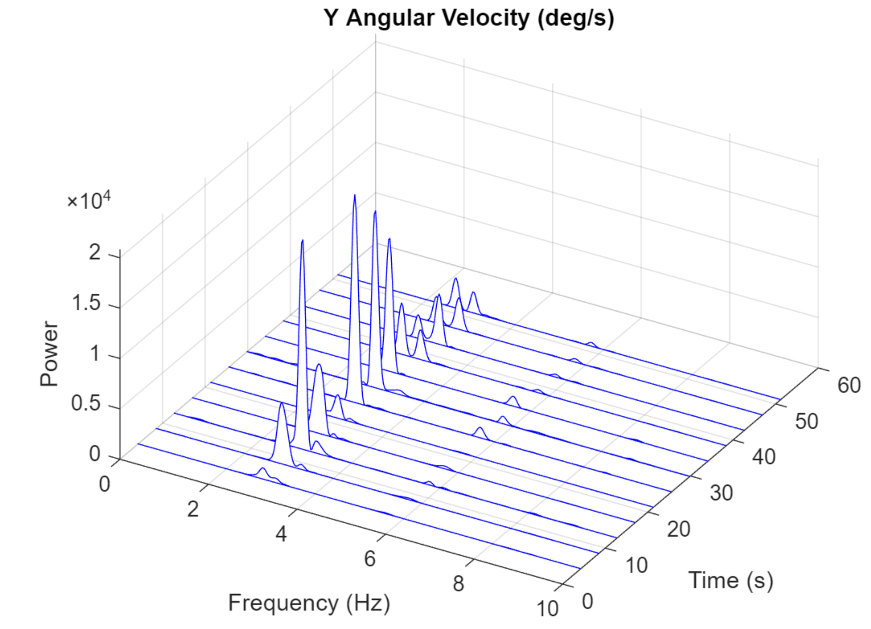

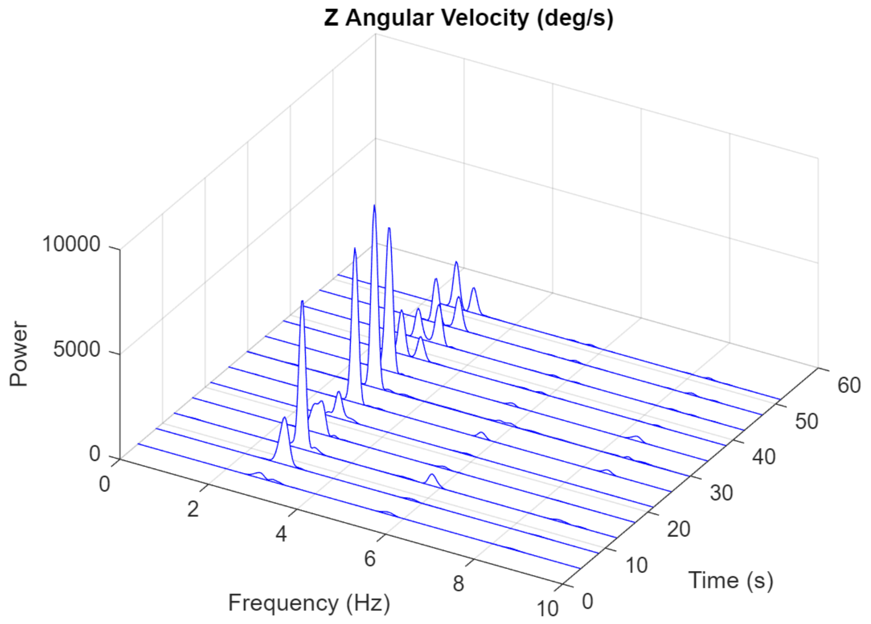

Now compute the time-frequency coherence spectra of x, y, z acceleration

```
Maximum and minimum coherence between 2 and 4 Hz, over all time:
Maximum coherence: 0.98
Minimum coherence: 0.00
Fraction of time with coherence > 0.8 in the 2-4 Hz band: 0.56
```

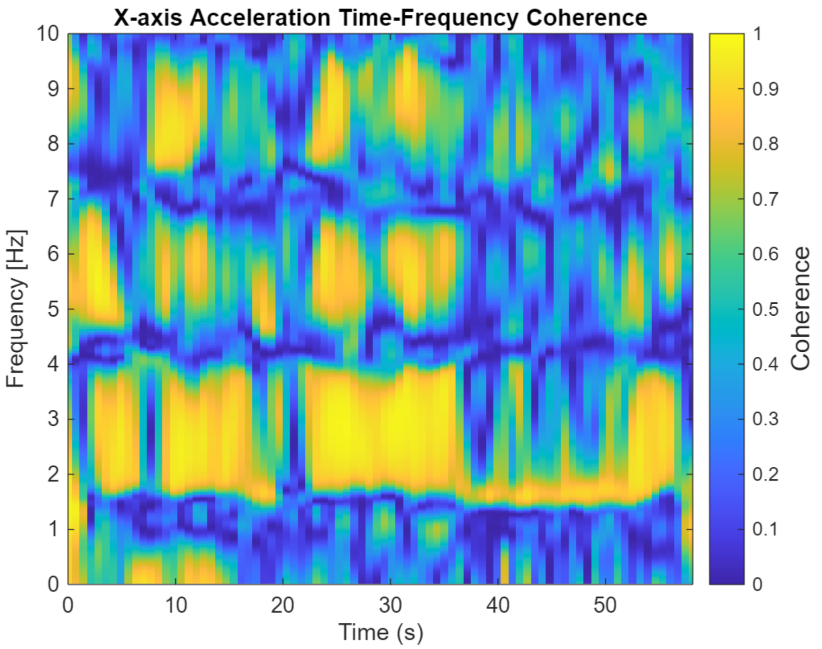


```
Maximum and minimum coherence between 2 and 4 Hz, over all time:
Maximum coherence: 0.98
Minimum coherence: 0.00
Fraction of time with coherence > 0.8 in the 2–4 Hz band: 0.49
```

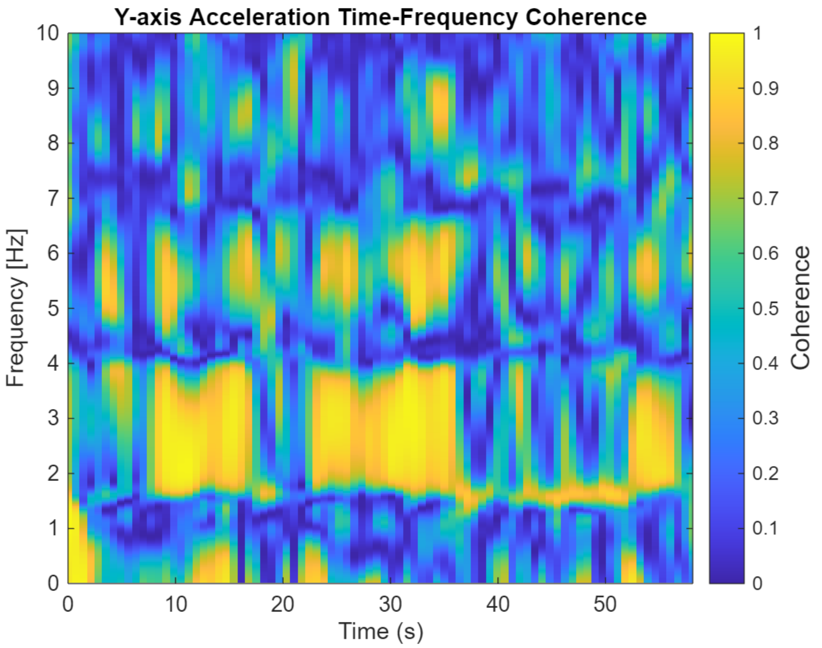


```
Maximum and minimum coherence between 2 and 4 Hz, over all time:
Maximum coherence: 0.99
Minimum coherence: 0.00
Fraction of time with coherence > 0.8 in the 2–4 Hz band: 0.46
```

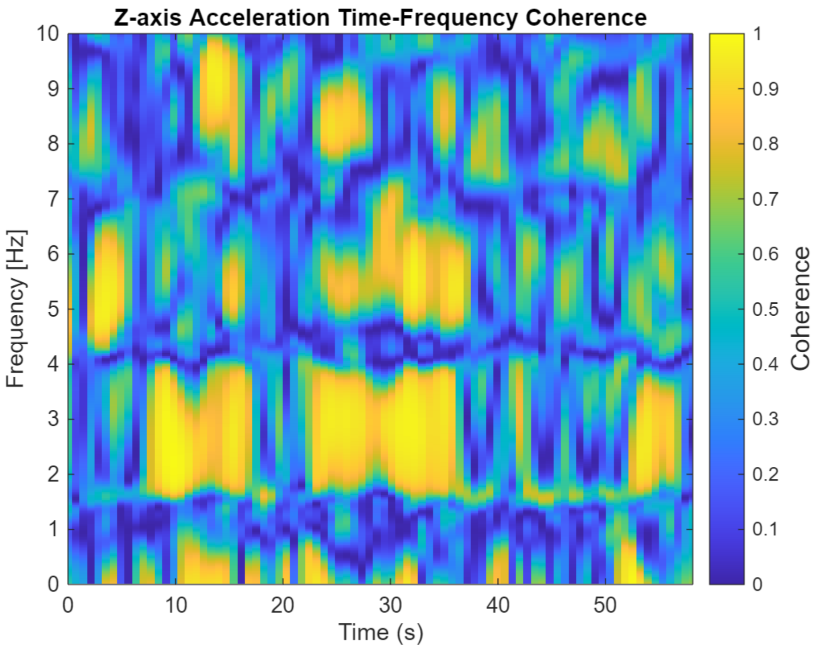


```
Maximum and minimum coherence between 2 and 4 Hz, over all time:
Maximum coherence: 0.99
Minimum coherence: 0.00
Fraction of time with coherence > 0.8 in the 2–4 Hz band: 0.46
```

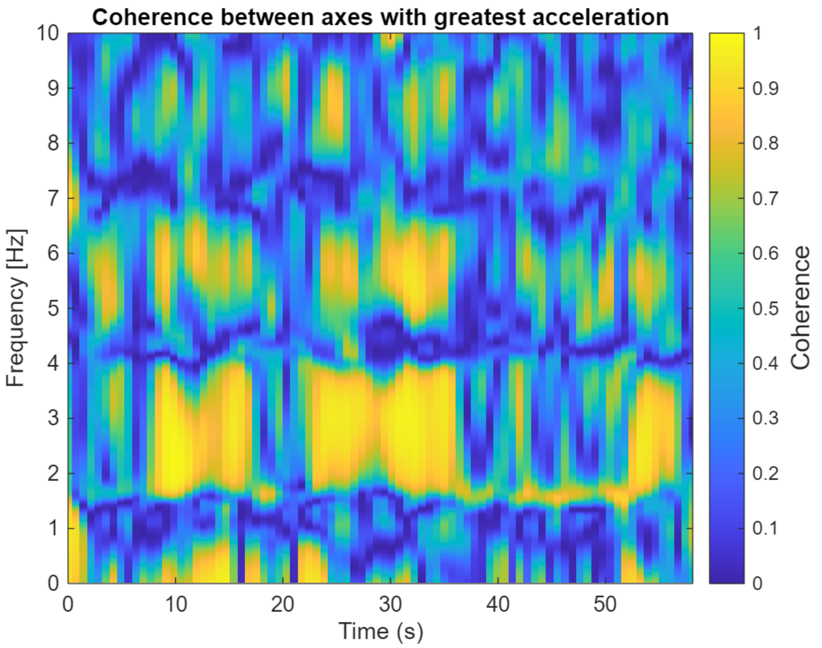


```
IMU #1 and #2 axes with greatest acceleration power: 2 and 3
```

Now compute the time-frequency coherence spectra of x, y, z angular velocity

```
Maximum and minimum coherence between 2 and 4 Hz, over all time:
Maximum coherence: 0.94
Minimum coherence: 0.00
Fraction of time with coherence > 0.8 in the 2–4 Hz band: 0.40
```

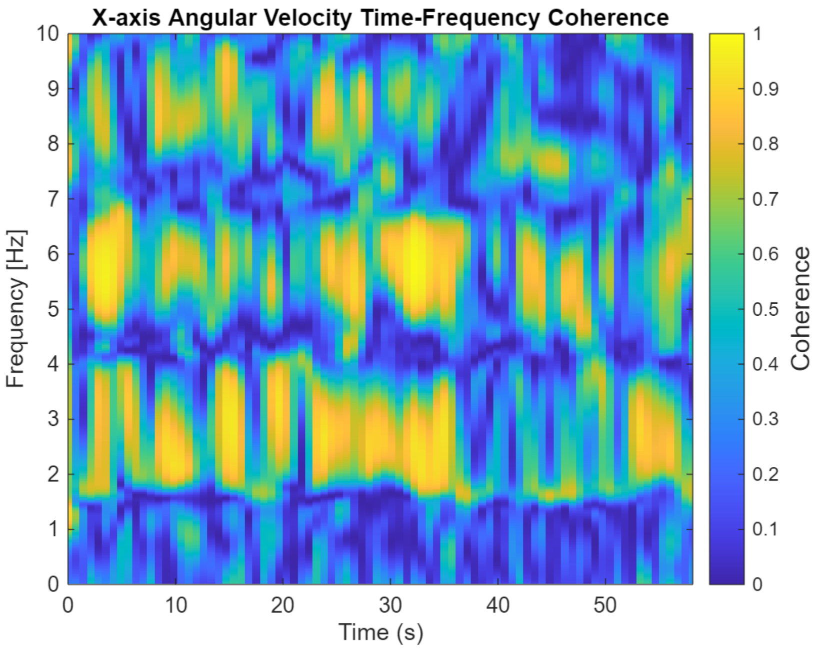


```
Maximum and minimum coherence between 2 and 4 Hz, over all time:
Maximum coherence: 0.98
Minimum coherence: 0.00
Fraction of time with coherence > 0.8 in the 2–4 Hz band: 0.51
```

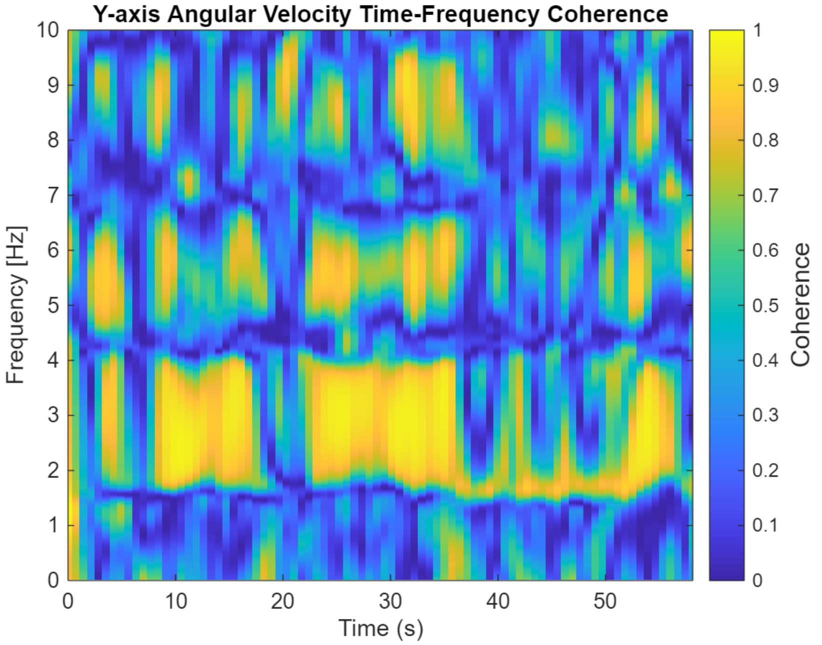


```
Maximum and minimum coherence between 2 and 4 Hz, over all time:
Maximum coherence: 0.99
Minimum coherence: 0.00
Fraction of time with coherence > 0.8 in the 2–4 Hz band: 0.52
```

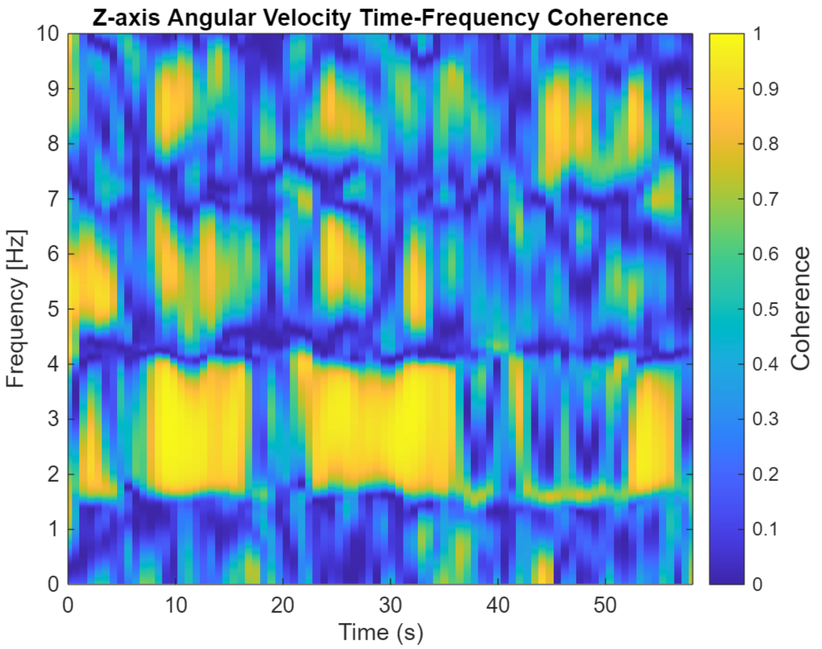


```
Maximum and minimum coherence between 2 and 4 Hz, over all time:
Maximum coherence: 0.98
Minimum coherence: 0.00
Fraction of time with coherence > 0.8 in the 2-4 Hz band: 0.51
```

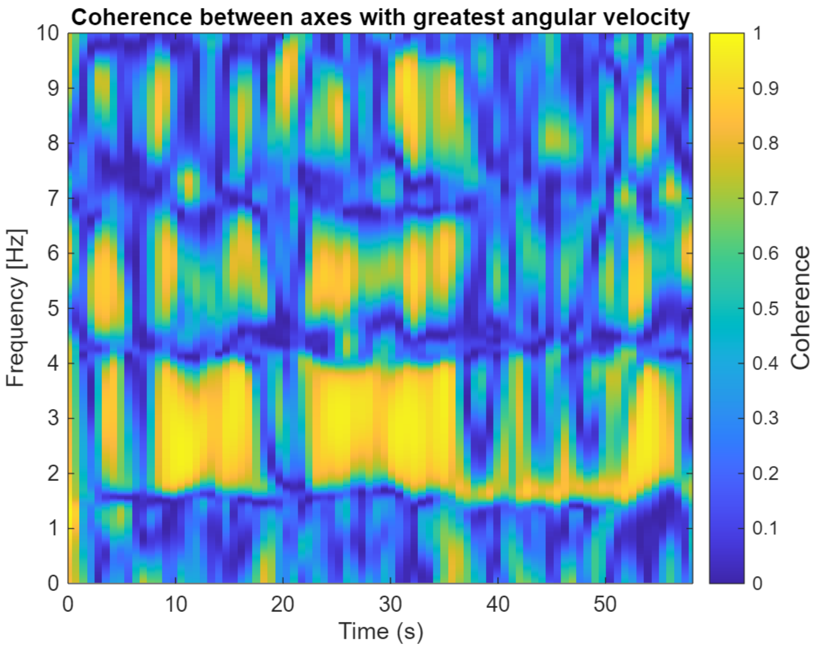


```
IMU #1 and #2 axes with greatest angular velocity power: 2 and 2
```

Now compute the wavelet time-frequency coherence spectra of x, y, z acceleration

```
Maximum and minimum coherence between 2 and 4 Hz, over all time:
Maximum coherence: 0.99
Minimum coherence: 0.00
Fraction of time with coherence > 0.8 in the 2 to 4 Hz frequency band: 0.71
```

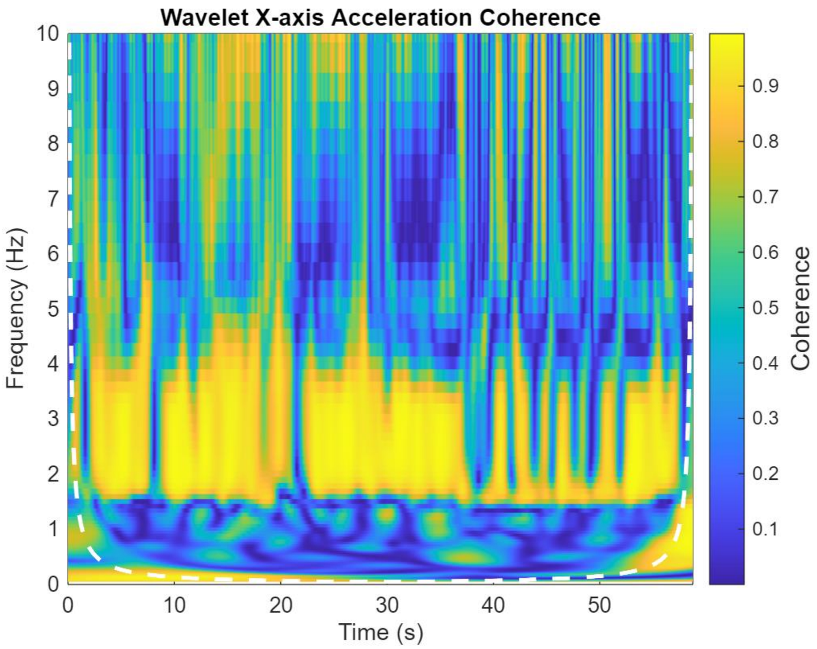


```
Maximum and minimum coherence between 2 and 4 Hz, over all time:
Maximum coherence: 1.00
Minimum coherence: 0.00
Fraction of time with coherence > 0.8 in the 2 to 4 Hz frequency band: 0.62
```

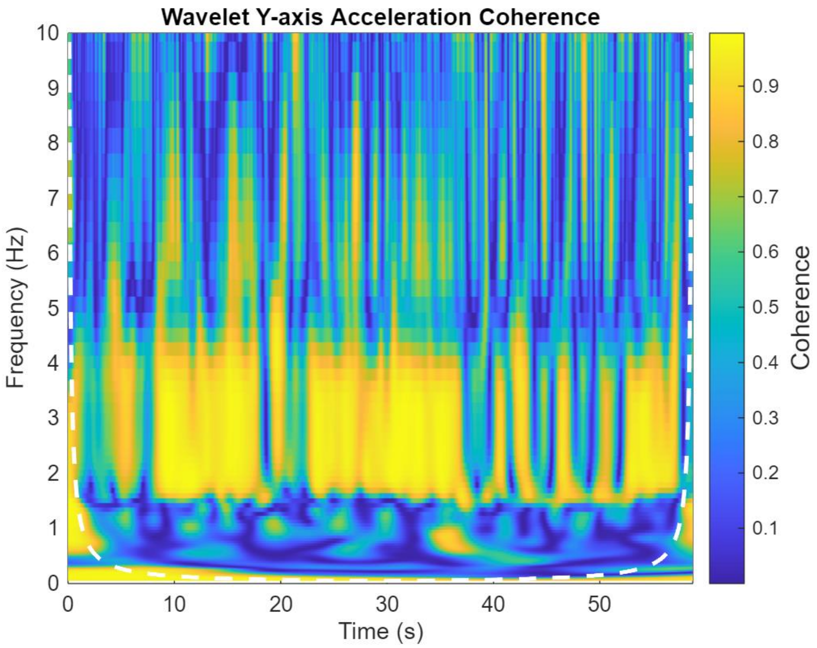


```
Maximum and minimum coherence between 2 and 4 Hz, over all time:
Maximum coherence: 1.00
Minimum coherence: 0.00
Fraction of time with coherence > 0.8 in the 2 to 4 Hz frequency band: 0.59
```

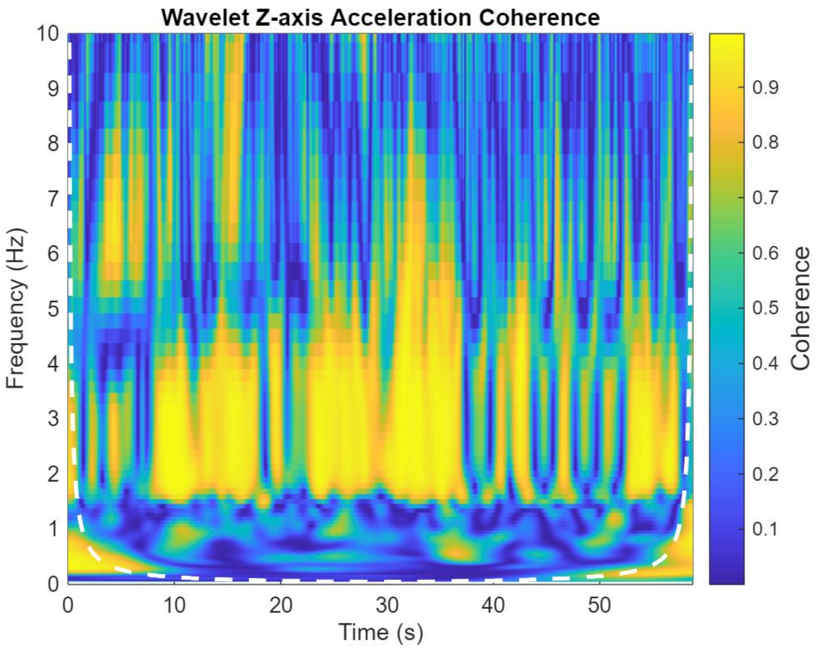


```
Maximum and minimum coherence between 2 and 4 Hz, over all time:
Maximum coherence: 1.00
Minimum coherence: 0.00
Fraction of time with coherence > 0.8 in the 2 to 4 Hz frequency band: 0.62
```

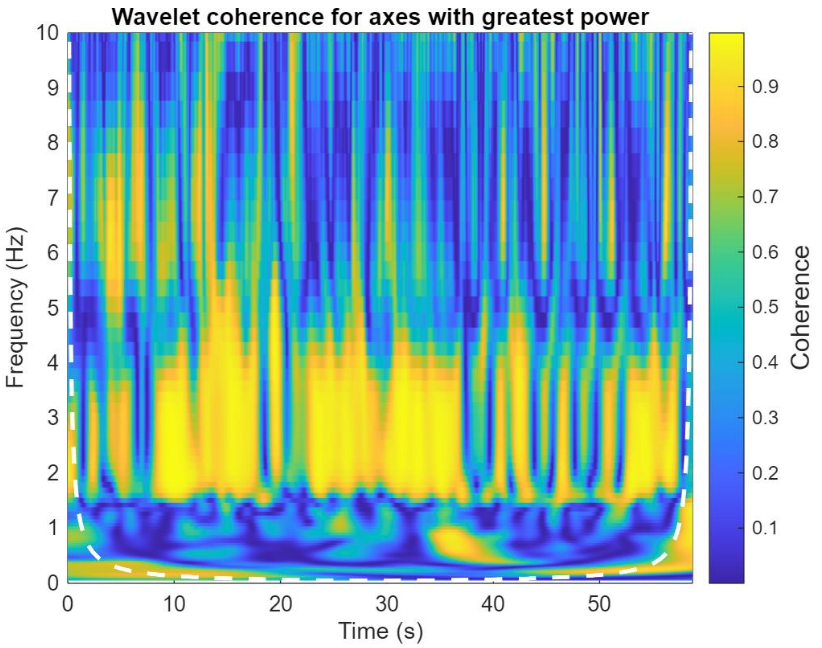


```
IMU #1 and #2 axes with greatest acceleration power: 2 and 3
```

Now compute the wavelet time-frequency coherence spectra of x, y, z angular velocity

```
Maximum and minimum coherence between 2 and 4 Hz, over all time:
Maximum coherence: 0.99
Minimum coherence: 0.00
Fraction of time with coherence > 0.8 in the 2 to 4 Hz frequency band: 0.63
```

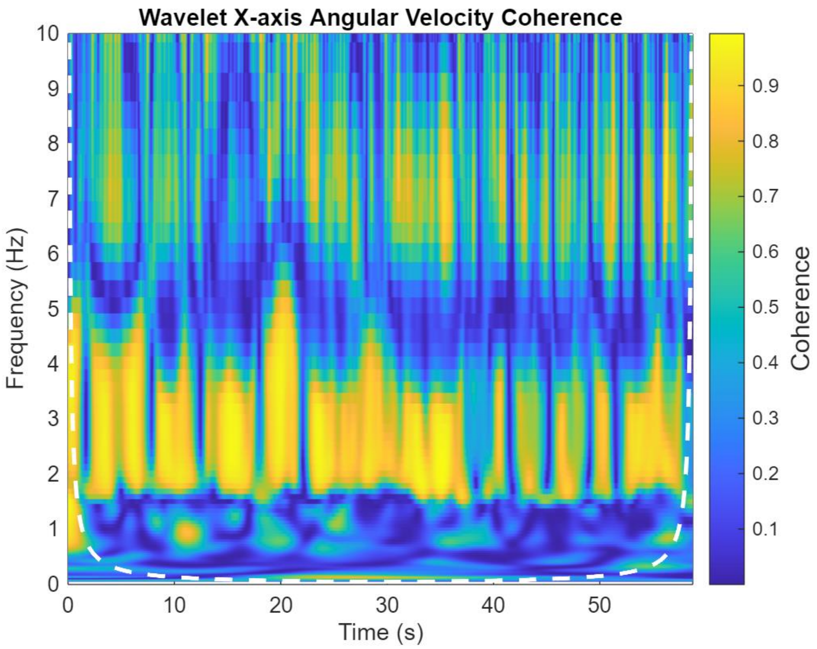


```
Maximum and minimum coherence between 2 and 4 Hz, over all time:
Maximum coherence: 0.99
Minimum coherence: 0.00
Fraction of time with coherence > 0.8 in the 2 to 4 Hz frequency band: 0.67
```

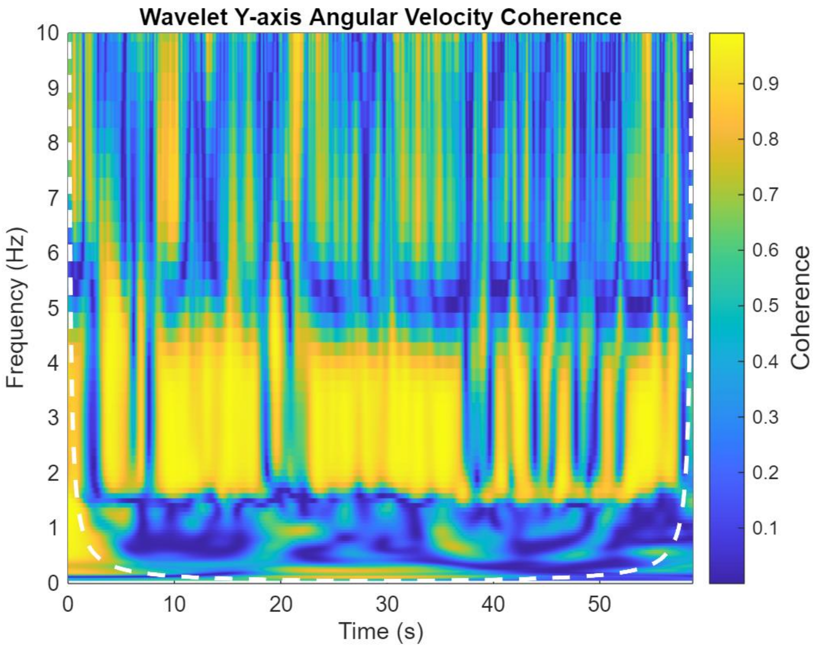


```
Maximum and minimum coherence between 2 and 4 Hz, over all time:
Maximum coherence: 0.99
Minimum coherence: 0.00
Fraction of time with coherence > 0.8 in the 2 to 4 Hz frequency band: 0.67
```

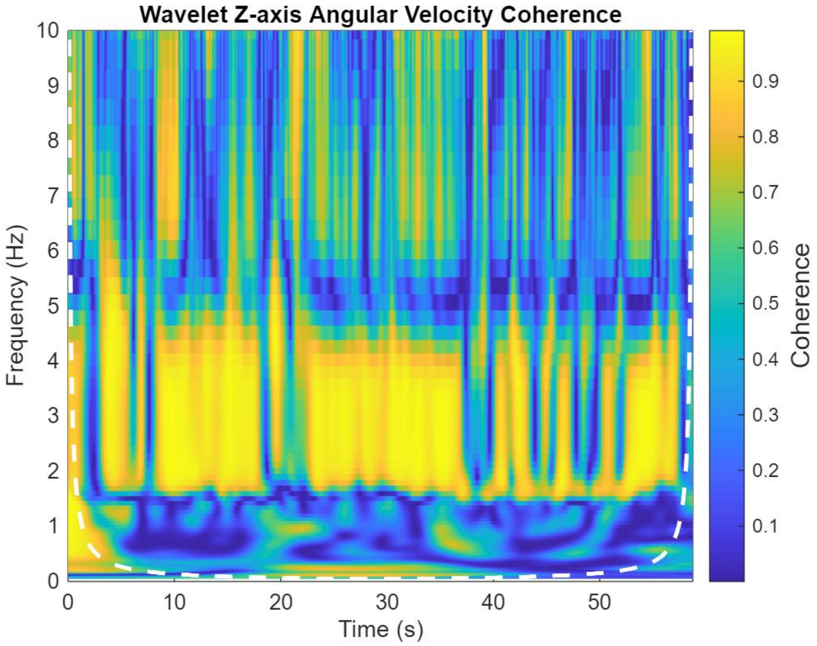


```
Maximum and minimum coherence between 2 and 4 Hz, over all time:
Maximum coherence: 0.99
Minimum coherence: 0.00
Fraction of time with coherence > 0.8 in the 2 to 4 Hz frequency band: 0.67
```

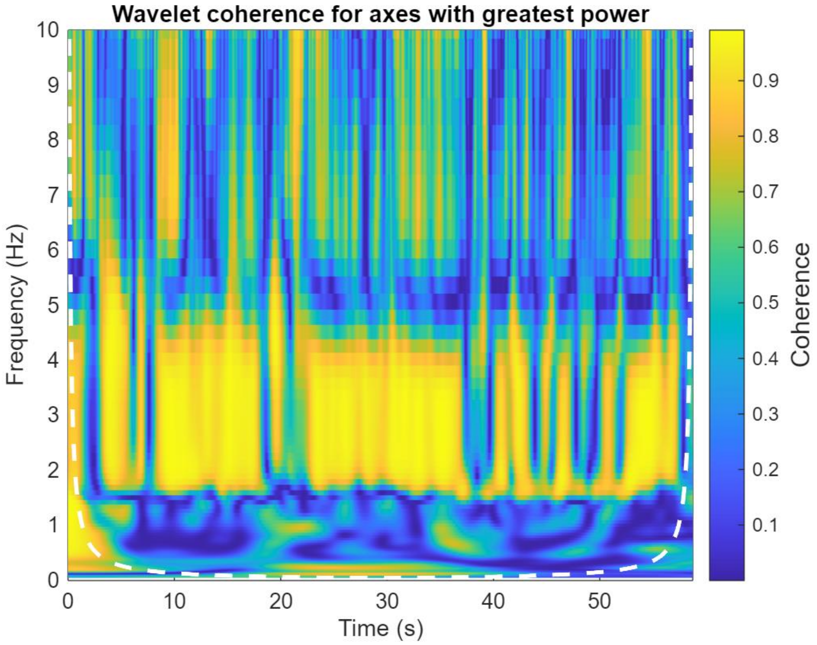


```
IMU #1 and #2 axes with greatest angular velocity power: 2 and 2
```

Functions
